# Resolution of 9,10-Diketo[7]helicene and Its Use in One-Step Preparation of Helicene-Based D–A–D Push–Pull Systems

**DOI:** 10.1021/acs.joc.4c00135

**Published:** 2024-05-28

**Authors:** Martin Kos, Tomáš Beránek, Ivana Císařová, Petra Cuřínová, Jaroslav Žádný, Jan Storch, Vladimír Církva, Martin Jakubec

**Affiliations:** † Research Group of Advanced Materials and Organic Synthesis, Institute of Chemical Process Fundamentals of the Czech Academy of Sciences, v. v. i., Rozvojová 135, 165 00 Prague 6, Czech Republic; ‡ Department of Inorganic Chemistry, Faculty of Science, Charles University in Prague, Hlavova 2030, 128 40 Prague 2, Czech Republic; § Department of Organic Chemistry, University of Chemistry and Technology, Technická 5, 166 28 Prague 6, Czech Republic

## Abstract

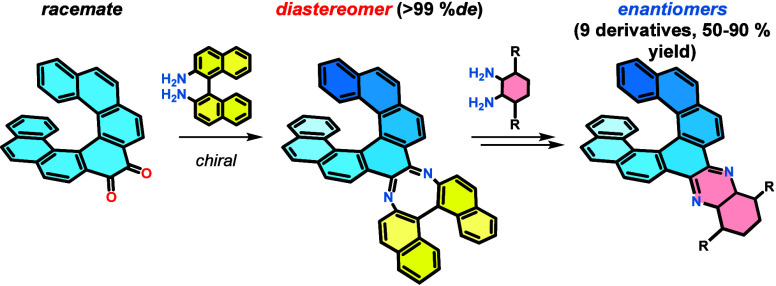

Racemic 9,10-diketo[7]helicene was successfully separated into enantiomers using a reversible and stereoselective reaction with 2,2′-diamino-1,1′-binaphthalene with moderate yields but with remarkable purity (>99% de). The enantiomerically pure diketone was used as a convenient starting material for the preparation of helicene-based push–pull molecules, which incorporated aza-aryl acceptors and diarylaminophenylene donor groups in a single step. A series of six push–pull systems, along with three reference molecules without donors, were prepared and studied using UV/vis and fluorescence measurements, circular dichroism, and DFT calculations.

## Introduction

Nonplanar polycyclic aromatic hydrocarbons (PAHs) have garnered significant interest in recent years as an alternative to planar aromatics and nanographenes. The appeal of these compounds stems from their advantageous properties, such as enhanced solubility, unique optical and electronic behaviors, and altered intermolecular contact compared to their planar counterparts.^1,2^ Among these, helicenes stand out as a particularly well-studied class, offering a rich array of properties not commonly found in planar systems.^3−6^ Despite being known for decades, the unique properties of these molecules are only now being utilized in a variety of applications. For example, helicenes have been used in devices detecting circularly polarized light (CPL)^7^ and have shown promise in the areas of nonlinear optics, spin filtering and plasmonics, and photoswitches.^8−13^ Most notably, the ability of helicenes to emit CPL^14^ offers opportunities for the development of next-generation CPL-responsive OLEDs and other light-emitting devices.^15,16^ However, wider application of helicenes in OLEDs is mainly hindered by their generally low fluorescence quantum yields caused by radiationless intersystem crossing (ISC), which can reach a quantum yield of 0.9 in helicenes.^17^

**Figure 1:**
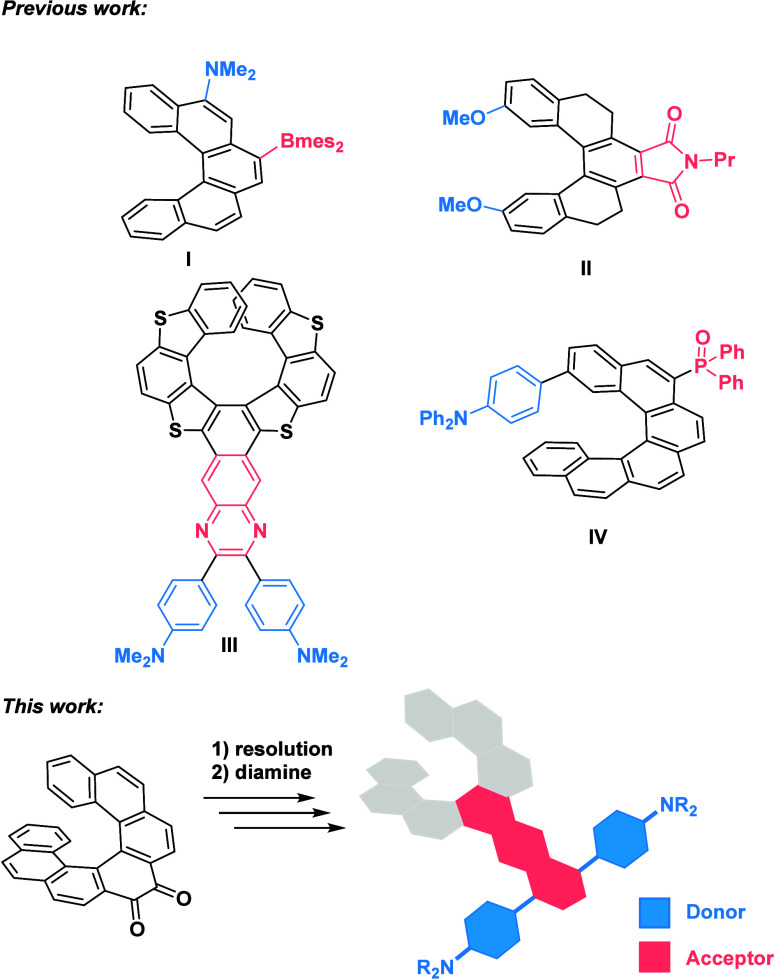
Selected examples of helicene-based push–pull systems and this work.

To optimize the attributes of helicenes for high-performance materials, targeted substitution patterns are often essential. One of the most effective ways to influence the extended π-system of helicenes involves creating a push–pull system by the introduction of both electron donors and electron acceptors interconnected via a π-system. The presence of such a system facilitates intramolecular charge transfer (ICT) interactions, which are responsible for enhancing the absorption of these molecules in the visible range, modifying the HOMO–LUMO energy gap, and altering the redox behavior of the compounds. Helicene-based push–pull systems have found applications in various fields. For example, simple [5]helicene and tetrahydro[5]helicene-based push–pull compounds (Figure 1, **I** and **II**) have displayed significantly improved fluorescence properties compared to unsubstituted helicene.^18−21^ In another study, tetrathia[9]helicene (Figure 1, **III**) was synthesized specifically to manipulate its excited-state dynamics and CPL properties.^22^ Recently, we published an example of 2,6-disubstituted push–pull systems based on [6]helicene phosphine oxides (Figure 1, **IV**) that exhibited significant solvatochromism.^23^ Some of these compounds have been employed in various molecular devices, including organic light-emitting diodes (OLEDs)^24^ and dye-sensitized solar cells (DSSCs).^25^

A significant challenge in the practical use of helicenes lies in obtaining them in their enantiomerically pure forms. The most effective strategy thus far for resolving racemic helicenes, while avoiding the use of chromatography on chiral stationary phases, has been the formation of diastereomers through the use of appropriate chiral agents. This methodology has successfully resolved helicene derivatives containing hydroxy-,^26^ thia-,^27^ and aza-functions,^28^ or carboxylic acids,^29^ among others.^30^ Interestingly, this methodology is typically used for helicenes modified at the terminal rings to ensure sufficient spatial interaction of the helicene’s chiral cavity with the chiral agent. The effectivity of this approach for helicenes substituted on their central rings is, to the best of our knowledge, very limited so far.

In this study, we aim to fill this gap by introducing a novel kinetic resolution technique for a helicene-based diketone, 9,10-diketo[7]helicene. This is followed by a one- step synthetic procedure to construct D-A-D push–pull systems with an appended helicene moiety. The synthesized compounds are subjected to characterization through UV/vis spectroscopy, fluorescence studies, measurements of circular dichroism (CD) spectra and DFT calculations.

## Results and Discussion

9,10-Diketo[7]helicene (**1**) serves as a versatile starting material for the preparation of various helicene-based molecules, particularly nitrogen-containing heterocycles, as previous studies have shown;^31−33^ however, it has not yet been possible to obtain their enantiomers.

Initially, we attempted to resolve **1** using a chiral HPLC. Although the analytical separation was sufficient (δ_RT_ = 5 min, Figure S21), the transition to the semipreparative mode was problematic due to long retention time and low solubility of **1**. The attempt to use a more soluble ethylene glycol diacetal derivative for chromatographic resolution did not lead to higher amounts of enantiopure **1**, as additional synthetic steps were required (Figure S22).

**Scheme 1:**
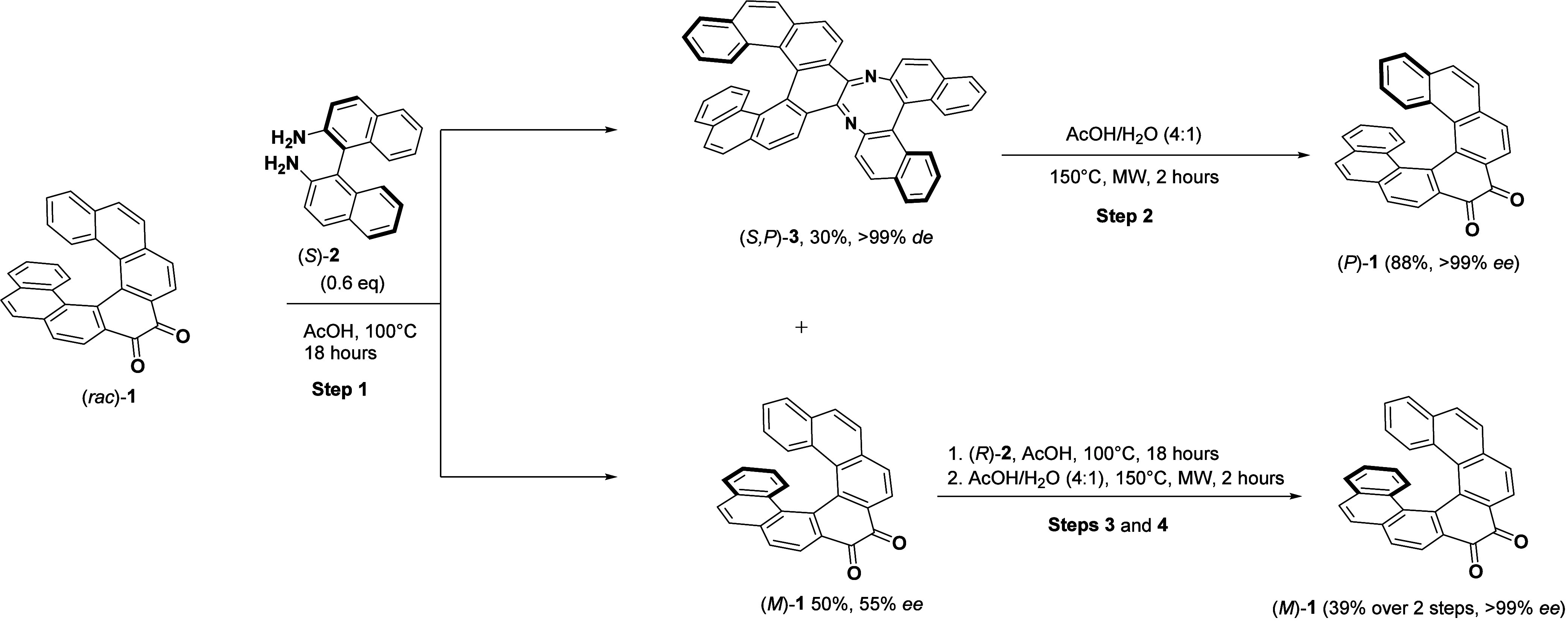
Resolution of *rac*-**1**

Consequently, we shifted our focus to kinetic resolution. Our choice of racemic 1,1′-binaphthyl-2,2′-diamine (*rac*-**2**) initially yielded a product, *rac*-**3**, whose X-ray structure (Figure S31) revealed the presence of only (*S*,*P*)- and (*R*,*M*)-enantiomers. Since the resulting diazacyclooctadiene ring is known to be sufficiently stable in aromatic compounds,^34^ while the axially chiral binaphthalenes retain their handedness at elevated temperatures,^35^ we expected that using only one enantiomer of **2** would yield a single diastereomer of **3**. The reaction of (*rac*)-**1** with one equivalent of (*S*)-**2** led to a relatively low conversion of the diketone, even after prolonged heating. However, the isolated adduct (*S,P*)-**3** was completely enantiomerically pure (>99% *de*). The reaction conditions were optimized to use 0.6 equiv of (*S*)-**2** in acetic acid, heated at 100 °C for 18 h (Scheme 1, Step 1). The product (*S*,*P*)-**3** was isolated by chromatography in a relatively low yield (30%) but with the expected diastereomeric excess (>99% *de*), while the residual enantioenriched diketone (*M*)-**1** was recovered in 50% yield and 55% *ee*. Hydrolysis of the resulting adduct (*S*,*P*)-**3** was achieved in a microwave reactor at 150 °C (Scheme 1, Step 2) and allowed for the recovery of enantiopure (*P*)-**1**, in 88% yield and >99% *ee*. The residual enantioenriched material from the first resolution step ((*M*)-**1**, 55% *ee*) was utilized in another resolution (step 3). Using the opposite enantiomer (*R*)-**2** provided (*R,M*)**-3** in 45% yield and >99% *de*, which upon hydrolysis of the adduct provided (*M*)-**1**, in comparable yield and purity (step 4). Ultimately, we achieved a 53% recovery of (*P*)-**1** and 39% of (*M*)-**1** in their enantiomerically pure forms, relative to their initial masses (26% and 20%, respective to the total starting amount). The absolute configuration of (*M*)-**1** and (*P*)-**1** was confirmed by comparing their ECD spectra with that of the parent [7]helicene. In the case of (*P*)-**1**, it was additionally confirmed by X-ray analysis (Figure S30).

**Scheme 2:**
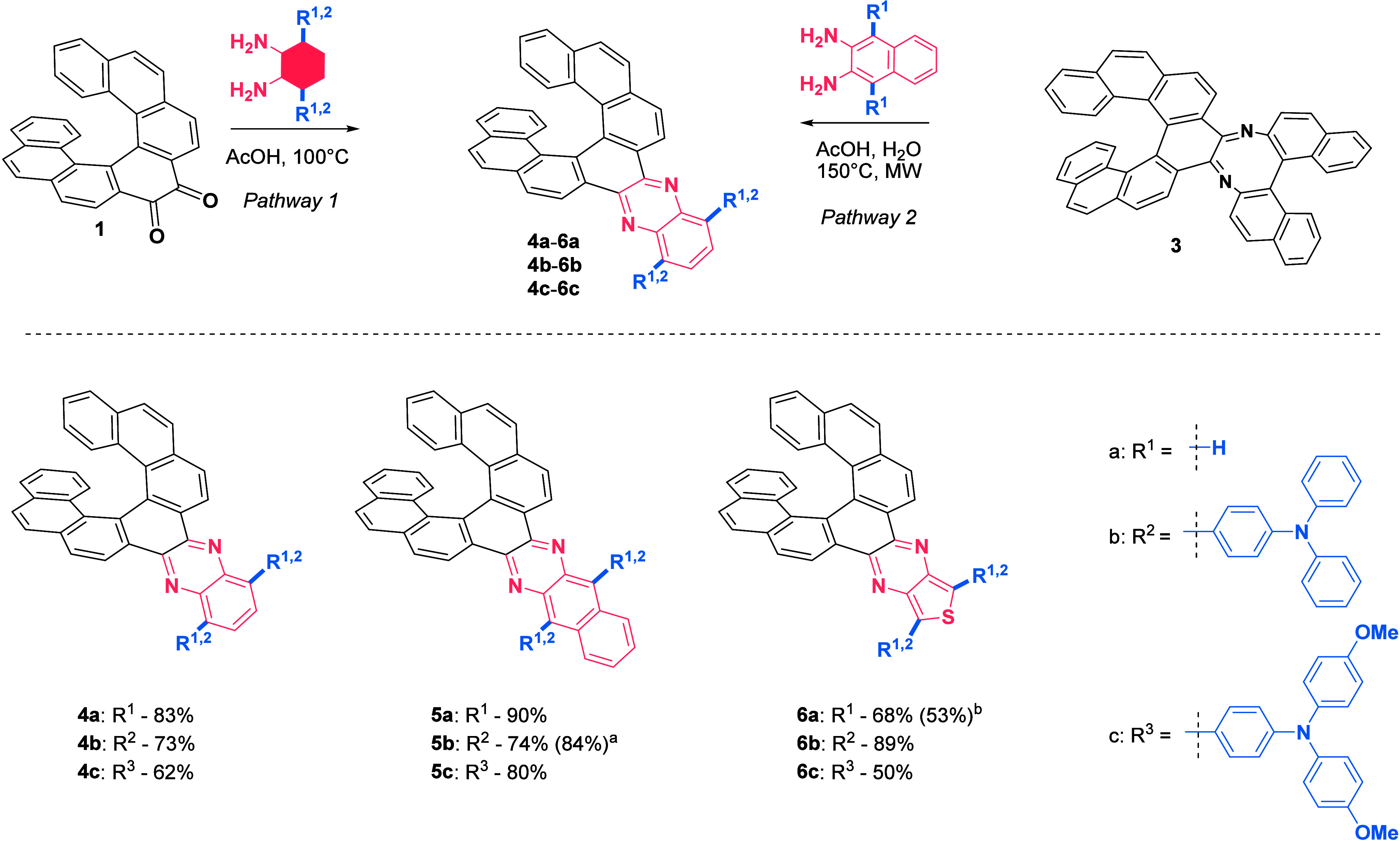
Preparation of Helicene-Based Push–Pull Systems Yield starting directly from **3**. Yield from a 1 mmol scale.

**Figure 2:**
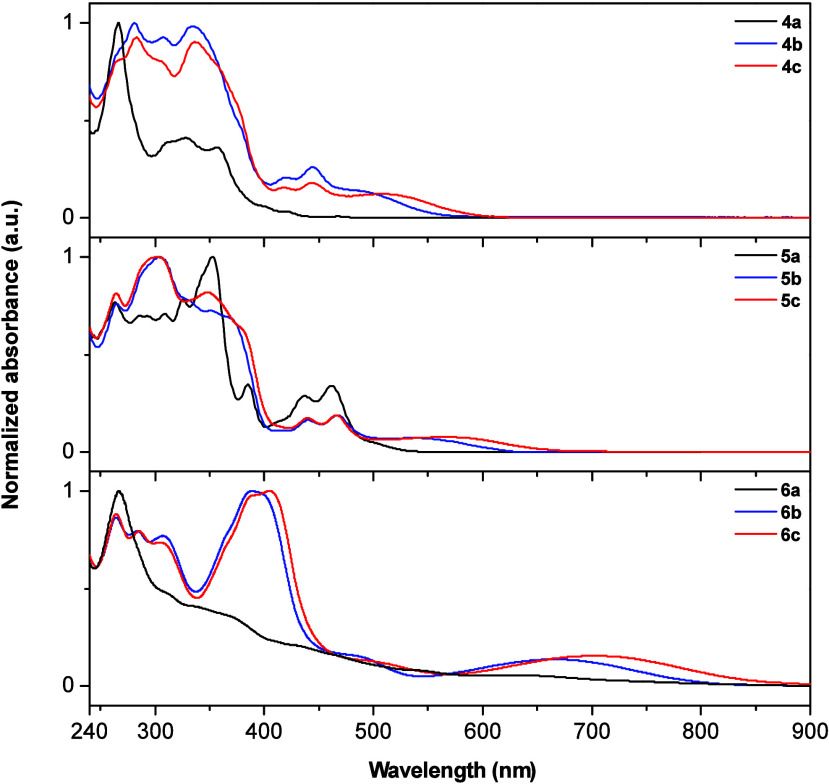
Normalized absorption spectra (5 × 10^–6^ M, CH_2_Cl_2_) of series of compounds **4a**–**c**, **5a**–**c**, and **6a**–**c**.

**Figure 3:**
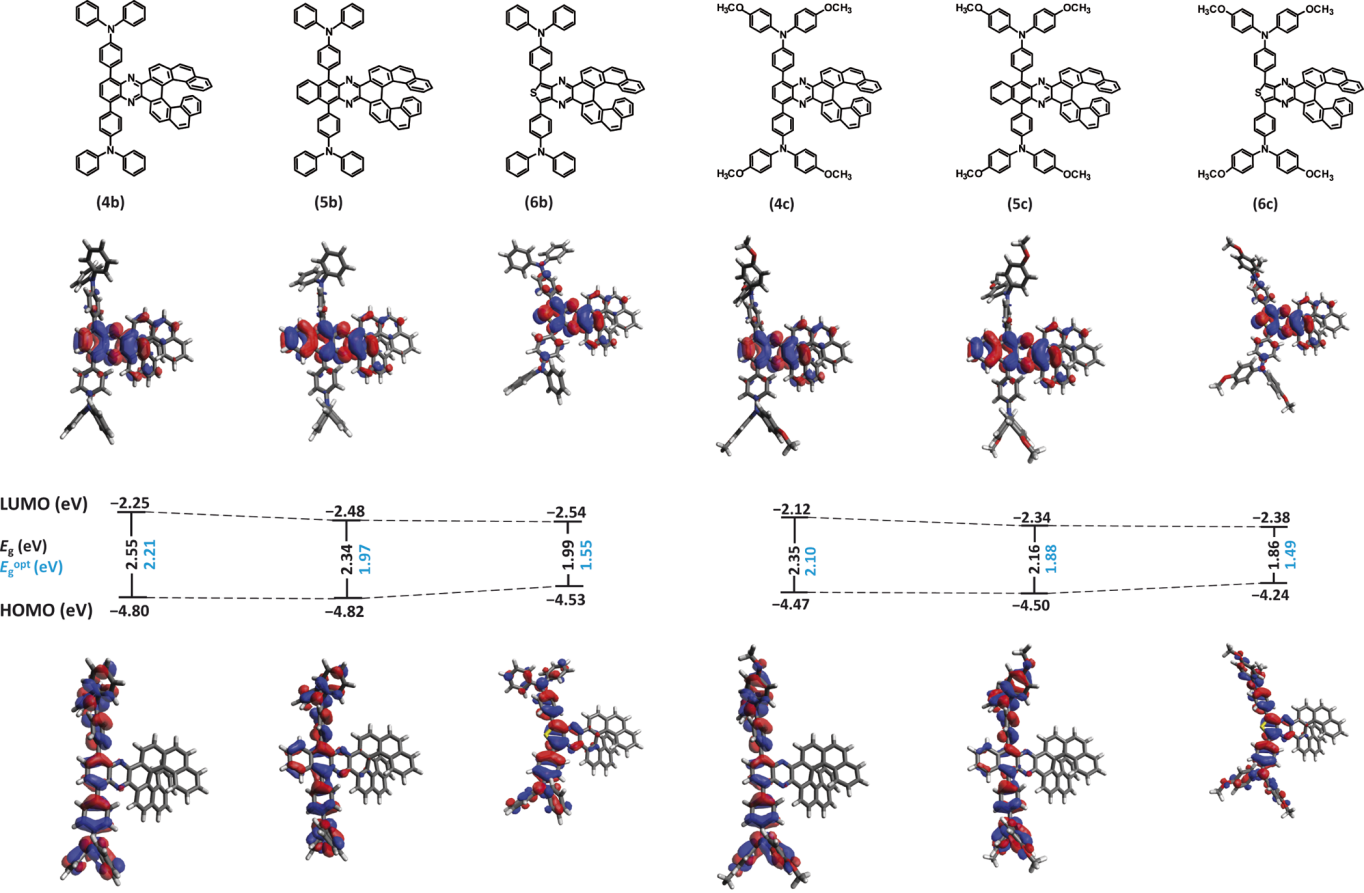
Computational values of HOMO–LUMO energy gaps (DFT method B3LYP/6-31G(d)) and the experimental values of optical HOMO–LUMO energy gaps (in blue, *E*_g_^opt^ = 1240/λ^edge^) for compounds **4b**–**6b** and **4c**–**6c**.

As previously mentioned, diketone **1** is an ideal precursor for the synthesis of nitrogen-containing heterocycles. Since these heterocycles are typically electro-deficient and behave as acceptors, we can create robust donor–acceptor–donor (D-A-D) push–pull systems by strategically introducing electron-donating moieties. Thus, **1** was reacted with suitably presubstituted *ortho*-diamine bearing two strong donors, resulting in the formation of a D-A-D push–pull system in a single step (Scheme 2, pathway 1). The *ortho*-diamines used for the condensation were based on phenyl, thienyl, and naphthyl moieties and were decorated with two different donor groups–diphenylaminophenylene and bis(4-methoxyphenyl)aminophenylene. The diamines were prepared by reduction of the corresponding thiadiaza- or nitro- species, respectively, and were used without further purification (for details see ESI, Schemes S4–S6). Heating the corresponding diamines with diketone **1** in acetic acid provided the products in yields ranging from 50% to 90% (Scheme 2, pathway 1). The same procedures were employed to prepare the enantiomers of the push–pull systems, starting from (*P*)**-1** and (*M*)**-1**, respectively, providing the products in comparable yields. Further practical improvement of the method was demonstrated when **3** was hydrolyzed in the presence of the corresponding diamine (Scheme 2, pathway 2). Under these conditions, the intermediate diketone **1** was directly transformed into the desired push–pull system. This approach reduced the number of steps by one and decreased the need for purification while increasing the overall yield. For instance, compound **5b** was prepared from **3** in a single step with an 84% yield, marking a 19% increase over the conventional step-by-step approach (Scheme 2, pathway 2). For comparative analysis and understanding of the influence of the triarylamine groups, we also synthesized three reference compounds, **4a**–**6a**. These compounds lack the triarylamine units, and their synthesis was achieved in their racemic forms. Their properties provided a basis to elucidate the distinct attributes introduced by the D-A-D system in our primary compounds. The optical properties of the prepared compounds were investigated by measuring their UV/vis and fluorescence spectra (Figure 2 and S33, Table 1). As anticipated, upon the incorporation of triarylamine, a redshift in absorption was observed. This effect was notably enhanced for the methoxy-substituted series **4c**–**6c**. A similar trend was observed for the modification of the acceptor part of the molecules—the most bathochromically shifted absorption maxima for both phenazines **4b** and **4c** were found at 487 and 507 nm, respectively, while for benzophenazines **5b** and **5c**, the maxima are located at 533 and 566 nm, respectively. The incorporation of a thiophene ring, as seen in **6b** and **6c**, induced a large bathochromic shift with absorbance peaks at 663 and 703 nm, both extending into the NIR wavelengths. Compounds **5a**–**c** and **6b,c** respectively exhibited minimal emission intensity, unlike **4a**–**c**, which exhibited a relatively intensive fluorescence maxima at 477, 645, and 699, respectively (Figure S33). Also, the unsubstituted derivative **6a** exhibits transitions resulting in a broadband (white) fluorescence upon excitation at 380 nm, with three intensive emission peaks located at 453, 481, and 566 nm. This feature is lost, however, with the introduction of the donors as described above. Compound **4b** was found to have the largest fluorescence quantum yield of the series (0.235, Table 1), while the other compounds had quantum yields typically below 0.10. The methoxy substituted compounds **4c**, **5c** and **6c**, as well as the series with thienyl core were particularly poor emitters, displaying quantum yields lower than 0.01. Additionally, the circular dichroism spectra (CD) of the enantiomerically pure versions of the prepared compounds were measured. In all cases, the respective enantiomers provided a mirror image of each other. The calculated g_abs_ values were all in the typical range of 10^–3^, reaching up to 3 × 10^–3^ (for details see ESI, Figure S34–S41). Interestingly, the charge-transfer band in all compounds was completely silent in CD spectra, indicating that there is no chirality transfer from helicene to the push–pull part of the molecule. This was confirmed by the DFT calculations performed on the optimized structures (Figure 3). The HOMO is predominantly located on the triarylaminedonors, while the LUMO is mainly situated on the acceptor part of the molecule, with only a small overlap with the helicene itself. Consequently, the CD response is primarily observed in the higher energy electronic transitions, where orbitals located on the helicene participate. The calculations also support the general trends observed experimentally. The introduction of the triarylamine donor increases the HOMO levels significantly, from −5.47 eV in the case of **4a** to −4.80 eV in the case of **4b** (see section 4.2 in the ESI). Further increase is achieved by introduction of methoxy groups in **4c** (−4.47 eV). On the other hand, the changes in LUMO levels are relatively small and all compounds are in the range from −2.54 eV to −2.12 eV. This behavior results in a gradual decrease in the HOMO–LUMO energy gap in the series which is overestimated by the DFT calculation (B3LYP/6-31G(d) level) by approximately 0.2–0.4 eV (vs *E*_*g*_^opt^). Among the series of compounds, **4a**–**c** showed the largest HOMO–LUMO energy gap. The π-extended series **5a**–**c** had relatively lower LUMO levels while maintaining HOMO energy at similar values compared to the series of compounds **4a**–**c**, and, finally, the series **6a**–**c** exhibited even lower energy gaps due to an increase of HOMO levels, compared to the two previous series.

**Table 1:** UV–vis and Fluorescence Data of **4a**–**c**, **5a**–**c**, and **6a**–**c**

Comp.	λ_max_^abs^ (nm)^a^	λ_0–0_^abs^ (nm)^b^	λ^edge^ (nm)^c^	λ_max_^em^ (nm)^d^	Φ_f_^e^
[7]^f^	269, 303	365	396	446	0.013
**4a**	266, 328	421	440	477	0.052
**4b**	281, 336	487	560	645	0.235
**4c**	283, 339	507	590	472, 699	<0.001
**5a**	266, 353	462	525	584	0.014
**5b**	302, 352	533	630	701	0.087
**5c**	303, 349	566	660	663, 836	<0.001
**6a**	264, 333	439	519	453, 566	0.083
**6b**	264, 387	663	800	481	<0.001
**6c**	264, 406	703	830	505	<0.001

a Absorption maxima in CH_2_Cl_2_ solution (5 × 10^–6^ M).

b Absorption 0–0 electronic transition wavelengths (for the ^1^L_*b*_ state).

c Optical absorption edges.

d Emission maxima in CH_2_Cl_2_ solution (5 × 10^–6^ M).

e 10^–6^ M in DCM.

f Ref (36) for [7]helicene.

## Conclusions

In this work, we developed a methodology to separate the enantiomers of 9,10-diketo[7]helicene (**1**) via diastereomer formation using binaphthalene diamine derivatives. The diimine adduct **3** was formed exclusively as (*S,P*)-, or (*R,M*)-pairs (>99% *de*), and its subsequent hydrolysis then provided the enantiomers of **1** in high enantiomeric purity and moderate yield. The adaptability of diketone **1** in forming complex helicene-based architectures was demonstrated by developing a range of push–pull systems, integrating aza-heterocycles as acceptors and diarylaminophenylene as donors. This one-step transformation was found to have a significant impact on the optical properties of the resulting helicenes, as evidenced by UV/vis, fluorescence, and circular dichroism measurements, as well as DFT calculations. The work addresses both the longstanding issue of enantiomeric separation of some helicene derivatives and introduces an easily accessible, enantiomerically pure synthon for functionalizing helicenes, setting the stage for their broader application in advanced materials and devices.

## Experimental Section

### Materials and Methods

Commercially available reagent grade materials were used as obtained from Sigma-Aldrich, Acros Organics, Apollo Scientific, and Fluorochem. (*R*)-(+)- and (*S*)-(−)-1,1′-Binaphthyl-2,2′-diamine (**2**), 4,7-dibromo-benzo[*c*][1,2,5]thiadiazole (**7**), 4-(diphenylamino)phenylboronic acid (**10b**), and 4-methoxy-*N*-(4-metho-xyphenyl)-*N*-(4-(4,4,5,5-tetramethyl-1,3,2-dioxaborolan-2-yl)phenyl)aniline (**10c**) were purchased from Fluorochem. Precursors 4,9-Dibromonaphtho[2,3-*c*][1,2,5]thiadiazole (**8**)^1^ and 2,5-dibromo-3,4-dinitrothiophene (**9**)^2^ were prepared according to the published procedures. Model compounds (*rac*)-diphenanthro[3,4-*a*:4′,3′-*c*]phenazine (**4a**) and (*rac*)-benzo[*i*]diphenanthro[3,4-*a*:4′,3′-*c*]phenazine (**5a**) were prepared by our previously published procedure.^3^ All solvents were of a reagent grade and used without any further purification, except for tetrahydrofuran and toluene, which were freshly distilled from sodium/benzophenone, and dichloromethane, which was freshly distilled from calcium hydride. Melting points were determined with Santiago KB T300 melting point apparatus (Czech Republic) and are uncorrected. TLC was carried out using silica gel 60 F254-coated aluminum sheets, and compounds were visualized with UV light (254 and 366 nm). Column chromatography was performed using Biotage HPFC systems (Isolera One) with prepacked flash silica gel columns. Microwave experiments were performed in a sealed vial (10 mL, borosilicate glass) on synthesis reactor Monowave 300 (Anton Paar GmbH) equipped with simultaneous temperature measurement using IR and a fiber optic sensor. Specific optical rotations ([α]_D_^20^) were measured at 589 and 880 nm in DCM at 20 °C on a JASCO P-2000 polarimeter with a Peltier cell holder and 1 dm path length cell. The values are given in deg cm^3^ g^–1^ dm^–1^ as an average value from 50 measurements. The standard Schlenk technique was used for all reactions. ^1^H and ^13^C{^1^H} NMR spectra were recorded using Bruker Avance spectrometer at 400 MHz (^1^H NMR) and 101 MHz (^13^C NMR). Chemical shifts (δ) are reported in parts per million (ppm) and referenced to residuals of CDCl_3_ (δ = 7.26 and 77.00 ppm, respectively), CD_2_Cl_2_ (δ = 5.30 and 54.00 ppm, respectively) or DMSO-*d*_6_ (δ = 2.50 and 39.52 ppm, respectively). The coupling constants (*J*) are given in hertz (Hz) and the corresponding multiplicity (s = singlet, d = doublet, t = triplet, m = multiplet). For exact mass measurement, the spectra were internally calibrated using Na-formate or APCI-TOF tuning mix. APCI high-resolution mass spectra were measured in a positive mode using a micrOTOF QIII mass spectrometer (Bruker) and were determined by software Compass Data Analysis. Absorption and fluorescence spectra were measured in a quartz cuvette with 1 cm optical path using a JASCO FP-8300 spectrofluorometer, UV–vis Spectrophotometer Varian CARY 50 CONC and Varian Eclipse spectrometer. The ECD and absorption spectra were measured on Jasco 1500 spectropolarimeter. The ECD and absorption spectra were measured over a spectral range of 220 to 600 and 800 nm respectively in DCM (1.0 × 10^–4^ M). Measurements were made in a quartz cell with a 0.2 cm path length using a scanning speed of 20 nm/min, a response time of 4 s, and standard instrument sensitivity. After a baseline correction, spectra were expressed in terms of differential molar extinction (Δε) and molar extinction (ε), respectively.

### Synthetic Procedures

#### (*S*,*P*)-**3**

A Schlenk flask was charged with 250 mg of (*rac*)-**1** (0.612 mmol, 1 equiv), 105 mg of (*S*)-**2** (0.367 mmol, 0.6 equiv), and 45 mL of degassed AcOH (0.014 M). The reaction mixture was stirred for 18 h at 100 °C under an inert atmosphere. After evaporation of the solvent, column chromatography using CHCl_3_/EtOAc (100:0 to 90:10) provided 120 mg of enantiopure (*S*,*P*)-**3** (0.183 mmol, 30%, >99% *de*) as a yellow solid and unreacted 125 mg of enantioenriched (*M*)-**1** (0.306 mmol, 50%, 55% *ee*) as a dark red solid. ^1^H NMR (400 MHz, CDCl_3_) δ 7.94–7.85 (m, 6H), 7.78 (d, *J* = 8.0 Hz, 2H), 7.55 (d, *J* = 8.7 Hz, 2H), 7.49–7.38 (m, 6H), 7.30–7.24 (m, 4H), 7.14 (d, *J* = 8.5 Hz, 2H), 6.95 (ddd, *J* = 8.0, 6.9, 1.1 Hz, 2H), 6.83 (d, *J* = 7.4 Hz, 2H), 6.38 (ddd, *J* = 8.4, 7.0, 1.4 Hz, 2H). ^13^C {^1^H} NMR (101 MHz, CDCl_3_) δ 166.79, 150.48, 135.30, 134.29, 133.23, 132.24, 131.77, 131.02, 129.92, 129.28, 129.16, 129.10, 128.44, 128.16, 127.27, 126.97, 126.66, 126.28, 126.13, 125.86, 125.30, 123.59, 122.94, 122.62, 120.74. R_f_ = 0.56 (CHCl_3_). HRMS (APCI/QTOF) *m*/*z* [M ]^+^ calculated for [C_50_H_28_N_2_]^+^ 656.2247 ; found 656.2252 (100%). Melting point: 279–286 °C. Optical rotation values: (*R,M*)-: [α]_589_^20^ = −2119 (DCM, 1.8 × 10^–3^ M); (*S,P*)-: [α]_589_^20^ = +2204 (DCM, 1.8 × 10^–3^ M)

#### (*P*)-9,10-Diketo[7]helicene (**1**)

A microwave vial was charged with 120 mg of (*S*,*P*)-**3** (0.183 mmol, >99% *de*). The vial was capped with PTFE septa and a 1.8 mL mixture of AcOH and H_2_O (4:1) was added. The reaction mixture was reacted in a microwave reactor for 2 h at 150 °C. After completion of the reaction, the mixture was extracted with DCM, dried over anhydrous MgSO_4_, filtered, and the solvent was evaporated under reduced pressure. The column chromatography using CHCl_3_/EtOAc (100:0 to 90:10) provided 63 mg of enantiopure (*P*)-**1** (0.154 mmol, 88%, >99% *ee*) as a dark red solid. ^1^H NMR (400 MHz, CD_2_Cl_2_) δ 8.27 (d, *J* = 8.0 Hz, 2H), 7.93 (d, *J* = 8.0 Hz, 2H), 7.59 (d, *J* = 8.7 Hz, 2H), 7.50 (d, *J* = 8.7 Hz, 2H), 7.28 (dd, *J* = 8.1, 1.3 Hz, 2H), 7.00 (ddd, *J* = 8.1, 7.0, 1.2 Hz, 2H), 6.66 (d, *J* = 8.4 Hz, 2H), 6.42 (ddd, *J* = 8.4, 6.9, 1.4 Hz, 2H). NMR spectra is in accordance with published data. Optical rotation values: (*M*)-: [α]_880_^20^ = −1072 (DCM, 5 × 10^–3^ M); (*P*)-: [α]_880_^20^ = +990 (DCM, 5 × 10^–3^ M)

#### General Procedure for the Reaction of Diketone **1** with Diamines

A Schlenk flask was charged with diketone **1** (1 equiv), diamine **12b,c**, **14b,c**, or **16b,c**, (2 equiv), and degassed AcOH (0.01–0.02M). The reaction mixture was stirred at 100 °C overnight under an inert atmosphere. After completion of the reaction (TLC), the product was purified by column chromatography or by crystallization.

#### (*rac*)-**4b**

The compound was prepared according to the general procedure using 100 mg of *rac*-**1**. Column chromatography using PE/CHCl_3_ (90:10 to 50:50) provided 168 mg (73%) of (*rac*)-**4b** as an orange-red solid. ^1^H NMR (400 MHz, CDCl_3_) δ 9.31 (d, *J* = 8.2 Hz, 2H), 8.09 (d, *J* = 8.3 Hz, 2H), 8.05 (s, 2H), 8.02–7.98 (m, 4H), 7.72 (d, *J* = 8.6 Hz, 2H), 7.48 (d, *J* = 8.6 Hz, 2H), 7.41–7.31 (m, 20H), 7.30–7.27 (m, 2H), 7.14–7.09 (m, 4H), 6.98–6.90 (m, 4H), 6.426 (ddd, *J* = 8.4, 7.0, 1.4 Hz, 2H). ^13^C {^1^H} NMR (101 MHz, CDCl_3_) δ 147.8 (4C), 147.4 (2C), 141.4 (2C), 140.2 (2C), 139.0 (2C), 133.4 (2C), 132.8 (2C), 132.0 (4C), 131.7 (2C), 130.1 (2C), 129.4 (2C), 129.3 (8C), 192.2 (4C), 128.6 (2C), 128.5 (2C), 128.1 (2C), 126.6 (2C), 125.7 (2C), 125.4 (2C), 124.9 (2C), 124.7 (8C), 123.6 (2C), 123.1 (2C), 123.02 (4C), 122.99 (4C). R_f_ = 0.21 (PE/CHCl_3_ 9:1). HRMS (APCI/QTOF) *m*/*z* [M + H]^+^ calculated for [C_72_H_47_N_4_]^+^ 967.3795 ; found 967.3791 (100%). Melting point: > 300 °C.

#### (*rac*)-**4c**

The compound was prepared according to the general procedure using 28 mg of *rac*-**1**. Reaction mixture was diluted with EtOH and filtered, which provided 45.2 mg (62%) of (*rac*)-**4c** as a dark orange solid. ^1^H NMR (400 MHz, CD_2_Cl_2_) δ 9.27 (d, *J* = 8.3 Hz, 2H), 8.09 (d, *J* = 8.2 Hz, 2H), 7.98 (s, 2H), 7.95–7.89 (m, 4H), 7.72 (d, *J* = 8.6 Hz, 2H), 7.47 (d, *J* = 8.6 Hz, 2H), 7.30–7.22 (m, 10H), 7.22–7.16 (m, 4H), 7.02–6.89 (m, 12H), 6.46–6.39 (m, 2H), 3.84 (s, 12H). ^13^C {^1^H} NMR (101 MHz, CD_2_Cl_2_) δ 156.22 (4C), 148.42 (2C), 141.24 (2C), 140.87 (4C), 140.27 (2C), 138.72 (2C), 133.39 (2C), 131.75 (2C), 131.73 (4C), 130.62 (2C), 130.10 (2C), 129.39 (2C), 129.13 (2C), 129.10 (2C), 128.51 (2C), 128.47 (2C), 128.00 (2C), 127.00 (8C), 126.57 (2C), 125.72 (2C), 125.33 (2C), 124.82 (2C), 123.63 (2C), 122.94 (2C), 119.43 (4C), 114.74 (8C), 55.49 (4C). R_f_ = 0.46 (CHCl_3_). HRMS (APCI/QTOF) *m*/*z* [M + H]^+^ calculated for [C_76_H_55_N_4_O_4_]^+^ 1087.4217; found 1087.4153 (100%). Melting point: 195–198 °C.

#### (*rac*)-**5b**

The compound was prepared according to the general procedure using 21 mg of *rac*-**1**. Column chromatography using PE/CHCl_3_ (50:50) provided 38 mg (74%) of (*rac*)-**5b** as a red solid. ^1^H NMR (400 MHz, CDCl_3_) δ 9.12 (d, *J* = 8.2 Hz, 2H), 8.38 (dd, *J* = 6.8, 3.3 Hz, 2H), 8.04 (d, *J* = 8.3 Hz, 2H), 7.80–7.72 (m, 2H), 7.68 (dd, *J* = 11.2, 8.4 Hz, 4H), 7.63–7.54 (m, 2H), 7.54–7.37 (m, 22H), 7.29–7.25 (m, 2H), 7.20–7.08 (m, 4H), 6.99–6.85 (m, 4H), 6.41 (ddd, *J* = 8.3, 6.9, 1.3 Hz, 2H). ^13^C {^1^H} NMR (101 MHz, CDCl_3_) δ 148.21 (4C), 147.28 (2C), 142.88 (2C), 137.79 (2C), 136.99 (2C), 133.97 (2C), 133.79 (2C), 133.73 (2C), 132.34 (2C), 131.93 (2C), 131.87 (2C), 130.51 (2C), 129.63 (2C), 129.58 (4C), 129.53 (8C), 128.61 (2C), 128.33 (2C), 127.78 (2C), 126.75 (2C), 126.27 (2C), 125.84 (2C), 125.57 (2C), 125.19 (2C), 124.58 (8C), 123.71 (2C), 123.46 (4C), 123.37 (2C), 123.03 (4C). R_f_ = 0.75 (PE/CHCl_3_ 33:67). HRMS (APCI/QTOF) *m*/*z* [M + H]^+^ calculated for [C_76_H_49_N_4_]^+^ 1017.3952 ; found 1017.3930 (100%). Melting point: 235–242 °C.

#### (*rac*)-**5c**

The compound was prepared according to the general procedure using 19 mg of *rac*-**1**. Column chromatography using PE/CHCl_3_/EtOAc (48:48:4) provided 43 mg (80%) of (*rac*)-**5c** as a dark brown solid.^1^H NMR (400 MHz, CDCl_3_) δ 9.14 (d, *J* = 8.2 Hz, 2H), 8.38 (dd, *J* = 6.9, 3.3 Hz, 2H), 8.04 (d, *J* = 8.3 Hz, 2H), 7.74–7.65 (m, 4H), 7.59 (d, *J* = 7.9 Hz, 2H), 7.55 (dd, *J* = 6.9, 3.2 Hz, 2H), 7.45 (d, *J* = 8.6 Hz, 2H), 7.40–7.27 (m, 14H), 7.04–6.87 (m, 12H), 6.41 (ddd, *J* = 8.3, 7.0, 1.4 Hz, 2H), 3.88 (s, 12H). ^13^C {^1^H} NMR (101 MHz, CDCl_3_) δ 155.98 (4C), 148.16 (2C), 142.77 (2C), 141.52 (4C), 137.83 (2C), 137.04 (2C), 133.92 (2C), 133.63 (2C), 133.56 (2C), 132.46 (2C), 131.87 (2C), 130.60 (2C), 129.78 (2C), 129.59 (2C), 129.57 (2C), 129.54 (2C), 128.58 (2C), 128.27 (2C), 127.89 (2C), 126.75 (8C), 126.09 (2C), 125.85 (2C), 125.54 (2C), 125.21 (2C), 123.69 (2C), 123.48 (2C), 120.27 (4C), 114.95 (8C), 55.72 (4C). We were unable to appropriately assign one of the carbon signals corresponding to 4 carbons, instead of 2. The total number of individual signals is however correct. R_f_ = 0.54 (CHCl_3_). HRMS (APCI/QTOF) *m*/*z* [M + H]^+^ calculated for [C_80_H_57_N_4_O_4_]^+^ 1137.4374; found 1137.4345 (100%). Melting point: 166–170 °C.

#### (*rac*)-**6b**

The compound was prepared according to the general procedure using 20 mg of *rac*-1. Reaction mixture was diluted with EtOH and filtered, the crude product was then purified by crystallization from DCM/MeCN (1:1) and provided 42 mg (89%) of (*rac*)-6b as a green powder. ^1^H NMR (400 MHz, CD_2_Cl_2_) δ 9.28 (d, *J* = 8.2 Hz, 1H), 8.47 (s, 2H), 8.07 (d, *J* = 8.3 Hz, 1H), 7.70 (d, *J* = 8.6 Hz, 1H), 7.48 (d, *J* = 8.7 Hz, 1H), 7.41–7.09 (m, 13H), 7.00–6.91 (m, 2H), 6.44 (t, *J* = 7.7 Hz, 1H). ^13^C NMR spectrum was not obtained due to low solubility of the compound. R_f_ = 0.62 (PE/EtOAc 1:1). HRMS (APCI/QTOF) *m*/*z* [M + H]^+^ calculated for [C_70_H_45_N_4_S]^+^ 973.3359 ; found 973.3349 (100%). Melting point: > 300 °C.

#### (*rac*)-**6c**

The compound was prepared according to the general procedure using 10 mg of *rac*-**1**. Reaction mixture was diluted with EtOH and filtered, the crude product was then purified by crystallization from DCM/MeCN (1:1) and provided 13 mg (50%) of (*rac*)-**6b** as a green powder. ^1^H NMR (400 MHz, DMSO-*d*_6_) δ 9.10 (s, 2H), 8.31 (s, 4H), 8.15 (s, 2H), 7.78 (s, 2H), 7.59 (d, *J* = 8.5 Hz, 2H), 7.41–7.35 (m, 2H), 7.21–7.15 (m, 8H), 7.04–6.95 (m, 14H), 6.81 (d, *J* = 8.0 Hz, 2H), 6.41 (s, 2H), 3.80 (s, 12H). ^13^C NMR spectrum was not obtained due to low solubility of the compound. R_f_ = 0.67 (PE/EtOAc 2:1). HRMS (APCI/QTOF) *m*/*z* [M + H]^+^ calculated for [C_74_H_53_N_4_O_4_S]^+^ 1093.3782 ; found 1093.3868 (100%). Melting point: >300 °C.

## Data Availability

The data underlying this study are available in the published article and its Supporting Information

## Supporting Information Available

Synthetic procedures, NMR spectra, X-ray crystallographic data, DFT calculation details, overview of physicochemical properties of chosen compounds, and chiral HPLC analyses (PDF)
FAIR data, including the primary NMR FID files, for compounds **1**, **3**, **4b**, **4c**, **5b**, **5c**, **6b**, **6c**, **11c**, **13b**, **13c**, **15b**, **15c**, and **17** (ZIP)
