# Supplementary material for: Resolution of 9,10-Diketo[7]helicene and Its Use in One-Step Preparation of Helicene-Based D–A–D Push–Pull Systems
Source: J Org Chem. 2024 May 28;89(11):7495–502. doi: 10.1021/acs.joc.4c00135 (PMC11165575; doi:10.1021/acs.joc.4c00135)
Supplement: Supplementary file 1 — jo4c00135_si_001.pdf [file jo4c00135_si_001.pdf]

Supporting Information

Resolution of 9,10-Diketo[7]helicene and Its Use in One-Step Preparation of Helicene-Based D-A-D Push-Pull Systems

Martin Kos[1], Tomáš Beránek[1], Ivana Císařová[2], Petra Cuřínová[1,3], Jaroslav Žádný[1], Jan Storch[1], Vladimír Církva[1], Martin Jakubec[1,*]

[1]Research Group of Advanced Materials and Organic Synthesis, Institute of Chemical Process Fundamentals of the Czech Academy of Sciences, v. v. i., Rozvojová 135, 165 00 Prague 6, Czech Republic

[2]Department of Inorganic Chemistry, Faculty of Science, Charles University in Prague, Hlavova 2030, 128 40 Prague 2, Czech Republic

[3] Department of Organic Chemistry, University of Chemistry and Technology, Technicka 5, 166 28, Prague 6, Czech Republic

Corresponding author: *) Martin Jakubec (jakubecm@icpf.cas.cz)

Content

1. General Information S-3
   1.1 Materials and Methods S-3
   1.2 Mass spectrometry S-3
   1.3 Luminescence S-3
   1.4 CD spectroscopy S-4
2. Synthetic Procedures S-5
3. NMR spectra S-20
4. DFT Calculation Details of **3**, **4**– **6** S-32
   4.1 Cartesian coordinates of optimized structures of **3**-**6** S-32
   4.2 Frontier MOs of compounds **4**-**6** S-57
5. Chiral-HPLC Analysis S-60
6. X-ray crystallographic data S-65
7. UV/Vis spectra, Fluorescence, Circular Dichroism and g_{abs} spectra S-68
8. References S-77

1. General Information

1.1 Materials and Methods

Commercially available reagent grade materials were used as obtained from Sigma-Aldrich, Acros Organics, Apollo Scientific, and Fluorochem. (*R*)-(+)- and (*S*)-(−)-1,1′-Binaphthyl-2,2′-diamine (**2**), 4,7-dibromo-benzo[*c*][1,2,5]thiadiazole (**7**), 4-(diphenylamino)phenylboronic acid (**10b**), and 4-methoxy-*N*-(4-metho-xyphenyl)-*N*-(4-(4,4,5,5-tetramethyl-1,3,2-dioxaborolan-2-yl)phenyl)aniline (**10c**) were purchased from Fluorochem. Precursors 4,9-Dibromonaphtho[2,3-*c*][1,2,5]thiadiazole (**8**)[1] and 2,5-dibromo-3,4-dinitrothiophene (**9**)[2] were prepared according to the published procedures. Model compounds (*rac*)-diphenanthro[3,4-*a*:4',3'-*c*]phenazine (**4a**) and (*rac*)-benzo[*i*]diphenanthro[3,4-*a*:4',3'-*c*]phenazine (**5a**) were prepared by our previously published procedure[3]. All solvents were of a reagent grade and used without any further purification, except for tetrahydrofuran and toluene, which were freshly distilled from sodium/benzophenone, and dichloromethane, which was freshly distilled from calcium hydride. Melting points were determined with Santiago KB T300 melting point apparatus (Czech Republic) and are uncorrected. TLC was carried out using silica gel 60 F254-coated aluminum sheets, and compounds were visualized with UV light (254 and 366 nm). Column chromatography was performed using Biotage HPFC systems (Isolera One) with prepacked flash silica gel columns. Microwave experiments were performed in a sealed vial (10 mL, borosilicate glass) on synthesis reactor Monowave 300 (Anton Paar GmbH) equipped with simultaneous temperature measurement using IR and a fiber optic sensor. Specific optical rotations ($[\alpha]_{D}^{20}$) were measured at 589 and 880 nm in DCM at 20 °C on a JASCO P-2000 polarimeter with a Peltier cell holder and 1 dm path length cell. The values are given in deg cm^3 g^{-1} dm^{-1} as an average value from 50 measurements. The standard Schlenk technique was used for all reactions.

^1H and $^{13}C\{^1H\}$ NMR spectra were recorded using Bruker Avance spectrometer at 400 MHz (^1H NMR) and 101 MHz (^{13}C NMR). Chemical shifts (δ) are reported in parts per million (ppm) and referenced to residuals of $CDCl_3$ (δ = 7.26 and 77.00 ppm, respectively), CD_2Cl_2 (δ = 5.30 and 54.00 ppm, respectively) or DMSO-d_6 (δ = 2.50 and 39.52 ppm, respectively). The coupling constants (*J*) are given in hertz (Hz) and the corresponding multiplicity (s = singlet, d = doublet, t = triplet, m = multiplet).

1.2 Mass spectrometry

For exact mass measurement, the spectra were internally calibrated using Na-formate or APCI-TOF tuning mix. APCI high-resolution mass spectra were measured in a positive mode using a micrOTOF QIII mass spectrometer (Bruker) and were determined by software Compass Data Analysis.

1.3 Luminescence

Absorption and fluorescence spectra were measured in a quartz cuvette with 1 cm optical path using a JASCO FP-8300 spectrofluorometer, UV-VIS Spectrophotometer Varian CARY 50 CONC and Varian Eclipse spectrometer. Absolute quantum yields (QYs) were measured using a Jasco spectrofluorometer FP-8300 equipped with an ILF-835 100 mm diameter Integrating Sphere accessory. All measurements were conducted at room temperature employing standard 10 mm quartz cuvette. To calibrate the instrument, calibrated light sources (Jasco ESC-842, ESC-843) were utilized. Data acquisition was performed under a nitrogen (5.0) atmosphere using the following measurement conditions: excitation and emission bandwidth of 5 nm, scanning speed set at 100 nm/min, and a data interval of 0.2 nm. All samples were measured within a concentration range of 10^{-6} M in degassed dichloromethane (samples were purged by argon bubbling for 15 minutes prior to measurement). For each sample, both the incident light intensity and fluorescence intensity (under both direct and indirect excitation) were recorded. Samples **4a**, **5a**, and **6a** were excited at 375 nm, 460 nm, and 385 nm, respectively. Samples **4b**, **5b** and **6b** were excited at 450 nm, 365 nm, and 380 nm respectively. The Quantum Yield Calculation

Program (FWQE-880) by Jasco was employed for the calculation of QYs. All QY values have been corrected for indirect excitation.

1.4 CD spectroscopy

The ECD and absorption spectra were measured on Jasco 1500 spectropolarimeter. The ECD and absorption spectra were measured over a spectral range of 220 nm to 600 nm and 800 nm respectively in DCM (1.0×10^{-4} M). Measurements were made in a quartz cell with a 0.2 cm path length using a scanning speed of 20 nm/min, a response time of 4 seconds, and standard instrument sensitivity. After a baseline correction, spectra were expressed in terms of differential molar extinction ($\Delta\varepsilon$) and molar extinction (ε), respectively.

2. Synthetic Procedures

General procedure 1 – Suzuki-Miyaura coupling

Conditions A:

A Schlenk flask was charged with dibromo arene **7** or **9** (1 equiv), boronic acid **10b** or boronic ester **10c** (2.5 equiv), *t*-BuONa (4 equiv), $Pd(dba)_2$ (0.1 equiv), XPhos (0.2 equiv), and dry PhMe (0.1M). The reaction mixture was immersed in an oil bath and stirred at 100°C overnight under an inert atmosphere. After completion of the reaction (TLC), the mixture was filtered, evaporated, and purified by column chromatography.

Conditions B:

A Schlenk flask was charged with dibromo arene **8** or **9** (1 equiv), boronic acid **10b** or boronic ester **10c** (2.5 equiv), Na_2CO_3 (4 equiv), and $PdCl_2(Ph_3P)_2$ (0.2 equiv). PhMe and distilled water (7:1, 0.1M) were added. The reaction mixture was immersed in an oil bath and stirred at 100° C overnight under an inert atmosphere[I]. After completion of the reaction (TLC), the mixture was extracted by DCM, dried over anhydrous Na_2SO_4, and purified by column chromatography.

General procedure 2 – Reduction by $LiAlH_4$

A Schlenk flask was charged with thiadiazole **11c** or **13b,c** (1 equiv), and dry Et_2O (0.02M). The reaction mixture was cooled to -10 °C, and a solution of $LiAlH_4$ in THF (1M, 10 equiv) was added dropwise. Then, the mixture was let slowly warm to room temperature. The reaction progress was monitored by change in color, which went from dark red/purple to transparent. After completion of the reaction (approximately 1 hour), the excess of $LiAlH_4$ was quenched by the addition of 4% NaOH. The mixture was extracted by DCM and dried over anhydrous Na_2SO_4. After evaporation of solvents, the diamines **12c** or **14b,c** were used without further purification in another step.

General procedure 3 – Reduction by Zn

Thiadiazole **11b** or dinitrothiophene **15b,c** (1 equiv) and zinc powder (61 equiv) were suspended in a mixture of $CHCl_3$ and EtOH (1:1, 0.1M), and heated to 70 °C using an oil bath. Then, 5 mL concentrated HCl was added dropwise through condenser while hydrogen gas violently evolved. Once the addition was complete, the reaction mixture was heated for an additional 1 hour. After cooling, the reaction mixture was quenched with a 1M solution of NaOH until the pH reached approximately 8. The resulting reaction mixture was extracted with DCM and organic phase was dried over $MgSO_4$, then evaporated to obtain brown amorphous solid. Due to the low stability of the product, it was used directly for condensation with diketone **1**.

General procedure 4 – Reaction of diketone **1** with diamines

A Schlenk flask was charged with diketone **1** (1 equiv), diamine **12b,c**, **14b,c**, or **16b,c**, (2 equiv), and degassed AcOH (0.01-0.02M). The reaction mixture was immersed in an oil bath and stirred at 100 °C overnight under an inert atmosphere. After completion of the reaction (TLC), the product was purified by column chromatography or by crystallization.

[I] Except for the preparation of thiophene derivative **15c**, which was stirred at 40°C due to its lower stability, the reaction conducted at a lower temperature resulted in a higher yield.

Protection of 9,10-diketo[7]helicene

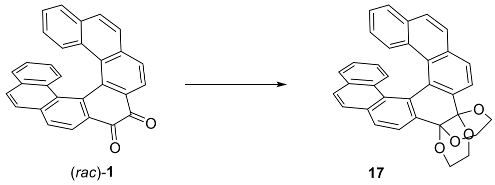

(*rac*)-**1** **17**

Scheme S1 *Protection of 9,10-diketo[7]helicene (**1**)*

Diketone (*rac)*-**1** (50 mg, 0.12 mmol) was suspended in 10 mL of toluene, followed by 1 mL of ethylene glycol. To the solution was added *p*-toluenesulfonic acid monohydrate (30 mg, 0.16 mmol) and the flask was equipped with a Dean-Stark apparatus. The reaction mixture was immersed in an oil bath and refluxed for 24 hours until full conversion was observed on TLC. The reaction mixture was then evaporated and the solid residue was filtered through a pad of silica gel, eluting with DCM. This provided 33 mg of protected **17** (0.067 mmol, 55%), as a yellow amorphous solid.

^{1}H NMR (400 MHz, $CDCl_3$) δ 8.02 (d, *J* = 8.0 Hz, 2H), 7.91 (d, *J* = 8.1 Hz, 2H), 7.56 (d, *J* = 8.7 Hz, 2H), 7.36 (d, *J* = 8.7 Hz, 2H), 7.22 (dd, *J* = 8.0, 1.3 Hz, 2H), 6.90 (ddd, *J* = 8.0, 6.9, 1.2 Hz, 2H), 6.63 (d, *J* = 8.4 Hz, 2H), 6.32 (ddd, *J* = 8.4, 6.9, 1.4 Hz, 2H), 4.61 (ddd, *J* = 12.2, 11.1, 3.2 Hz, 2H), 3.95 (dd, *J* = 12.0, 3.2 Hz, 2H), 3.81 (td, *J* = 12.2, 3.2 Hz, 2H), 3.43 (dd, *J* = 11.1, 3.1 Hz, 2H).

^{13}C {^{1}H} NMR (101 MHz, $CDCl_3$) δ 133.9 (2C), 132.7 (2C), 132.0 (2C), 130.8 (2C), 129.7 (2C), 129.5 (2C), 128.8 (2C), 127.6 (2C), 126.6 (2C), 126.3 (2C), 125.7 (2C), 125.7 (2C), 123.6 (2C), 123.4 (2C), 93.2 (2C), 63.7 (2C), 59.2 (2C).

R_f = 0.60 ($CHCl_3$/EtOAc 95: 5).

HRMS (APCI/QTOF) *m/z* $[M]^+$ calculated for $[C_{34}H_{24}O_4]^+$ 496.1669 ; found 496.1668 (100%).

Deprotection of 9,10-diketo[7]helicene

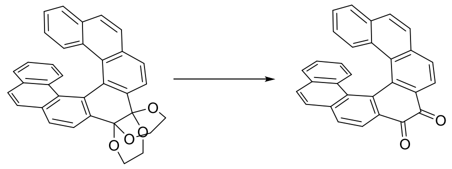

17 (*rac*)-**1**

Scheme S2 *Deprotection of 9,10-diketo[7]helicene (**1**)*

The protected diketone **17** (25 mg) was dissolved in 1:1 mixture of THF and 10% HCl (1:1, 5 ml) and heated to reflux using an oil bath. After 6 days the conversion was still incomplete, therefore making this approach inconvenient and it was not further optimized.

Optical resolution of 9,10-diketo[7]helicene (1)

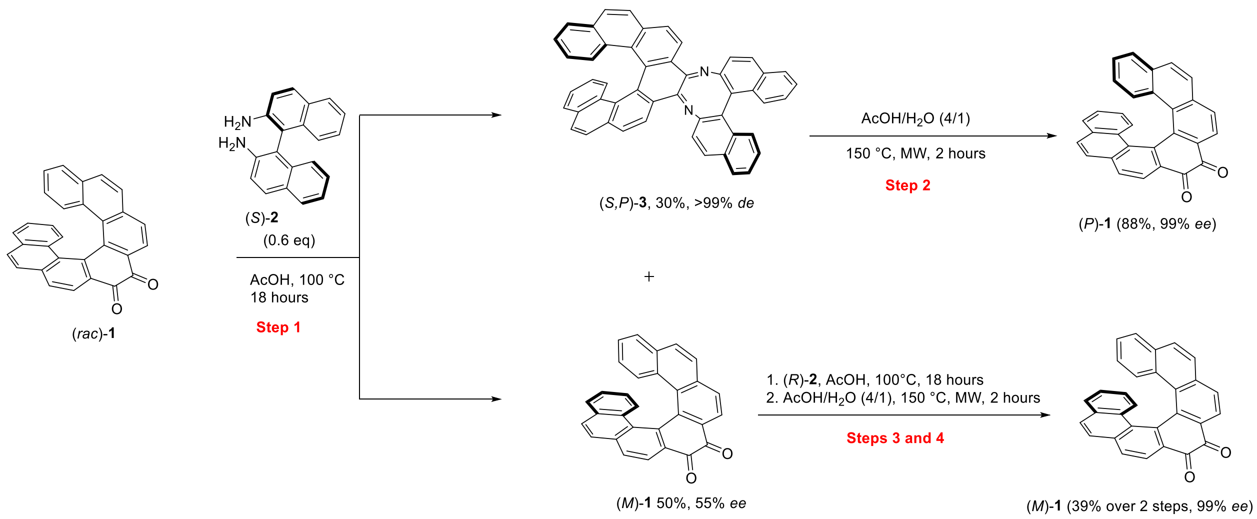

***Scheme S3** Optical resolution of 9,10-diketo[7]helicene (**1**)*

Step 1

A Schlenk flask was charged with 250 mg of (*rac*)-**1** (0.612 mmol, 1 equiv), 105 mg of (*S*)-**2** (0.367 mmol, 0.6 equiv), and 45 mL of degassed AcOH (0.014 M). The reaction mixture was immersed in an oil bath and stirred for 18 hours at 100 °C under an inert atmosphere. After evaporation of the solvent, column chromatography using $CHCl_3$/EtOAc (100:0 to 90:10) provided 120 mg of enantiopure (*S*,*P*)-**3** (0.183 mmol, 30%, >99% *de*) as a yellow solid and unreacted 125 mg of enantioenriched (*M*)-**1** (0.306 mmol, 50%, 55% *ee*) as a dark red solid.

Step 2

A microwave vial was charged with 120 mg of (*S*,*P*)-**3** (0.183 mmol, >99% *de*). The vial was capped with PTFE septa and a 1.8 mL mixture of AcOH and H_2O (4:1) was added. The reaction mixture was reacted in a microwave reactor for 2 hours at 150 °C. After completion of the reaction, the mixture was extracted with DCM, dried over anhydrous $MgSO_4$, filtered, and the solvent was evaporated under reduced pressure. The column chromatography using $CHCl_3$/EtOAc (100:0 to 90:10) provided 63.0 mg of enantiopure (*P*)-**1** (0.154 mmol, 84%, >99% *ee*) as a dark red solid.

Step 3

A Schlenk was charged with 125 mg of enantioenriched (*M*)-**1** (0.306 mmol, 55% *ee,* 1 equiv), 79 mg of (*R*)-**2** (0.275 mmol, 0.9 equiv), and 22 mL of degassed AcOH (0.014 M). The reaction mixture was immersed in an oil bath and stirred for 18 hours at 100 °C under an inert atmosphere. After evaporation of solvent, column chromatography using $CHCl_3$/EtOAc (100:0 to 90:10) provided 90 mg of enantiopure (*R*,*M*)-**3** (0.138 mmol, 45%, >99% *de*) as a yellow solid and unreacted 46 mg of enantioenriched (*P*)-**1** (0.113 mmol, 37%, 39% *ee*) as a dark red solid.

Step 4

A microwave vial was charged with 90.0 mg of (*R*,*M*)-**3** (0.138 mmol, >99% *de*). The vial was capped with PTFE septa and 1.1 mL mixture of AcOH/H_2O (4:1) was added. The reaction mixture was reacted in a microwave reactor for 2 hours at 150 °C. After the reaction, the mixture was extracted with DCM, dried over anhydrous $MgSO_4$, filtered and the solvent was evaporated at the reduced pressure. The column

chromatography using $CHCl_3$/EtOAc (100:0 to 90:10) provided 49.0 mg of enantiopure (*M*)-**1** (0.121 mmol, 88%, >99% *ee*) as a dark red solid.

(*R*,*M*)- or (*S*,*P*)-**3**

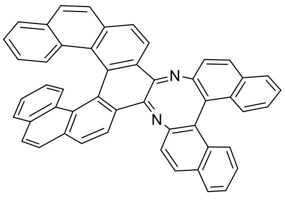

(*S*,*P*)- or (*R*,*M*)-(**3**)

^{1}H NMR (400 MHz, $CDCl_3$) δ 7.94 – 7.85 (m, 6H), 7.78 (d, *J* = 8.0 Hz, 2H), 7.55 (d, *J* = 8.7 Hz, 2H), 7.49 – 7.38 (m, 6H), 7.30 – 7.24 (m, 4H), 7.14 (d, *J* = 8.5 Hz, 2H), 6.95 (ddd, *J* = 8.0, 6.9, 1.1 Hz, 2H), 6.83 (d, *J* = 7.4 Hz, 2H), 6.38 (ddd, *J* = 8.4, 7.0, 1.4 Hz, 2H).

^{13}C {^{1}H} NMR (101 MHz, $CDCl_3$) δ 166.8, 150.5, 135.3, 134.3, 133.2, 132.2, 131.8, 131.0, 129.9, 129.3, 129.2, 129.1, 128.4, 128.2, 127.3, 127.0, 126.7, 126.3, 126.1, 125.9, 125.3, 123.6, 122.9, 122.6, 120.7.

R_f = 0.56 ($CHCl_3$).

HRMS (APCI/QTOF) *m/z* $[M]^+$ calculated for $[C_{50}H_{28}N_2]^+$ 656.2247 ; found 656.2252 (100%).

Melting point: 279.0-286.0 °C.

Optical rotation values:

(*R,M*)-: $[\alpha]_{589}^{20}$ = − 2119 (DCM, 1.8×10^{-3} M)

(*S,P*)-: $[\alpha]_{589}^{20}$ = +2204 (DCM, 1.8×10^{-3} M)

(*P*)- or (*M*)-9,10-diketo[7]helicene (1)

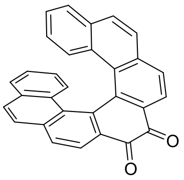

(rac)-**1**

^{1}H NMR (400 MHz, CD_2Cl_2) δ 8.27 (d, *J* = 8.0 Hz, 2H), 7.93 (d, *J* = 8.0 Hz, 2H), 7.59 (d, *J* = 8.7 Hz, 2H), 7.50 (d, *J* = 8.7 Hz, 2H), 7.28 (dd, *J* = 8.1, 1.3 Hz, 2H), 7.00 (ddd, *J* = 8.1, 7.0, 1.2 Hz, 2H), 6.66 (d, *J* = 8.4 Hz, 2H), 6.42 (ddd, *J* = 8.4, 6.9, 1.4 Hz, 2H).

NMR spectra is in accordance with published data[3].

Optical rotation values:

(*M*)-: $[\alpha]_{880}^{20}$ = − 1072 (DCM, 5×10^{-3} M)

(*P*)-: $[\alpha]_{880}^{20}$ = +990 (DCM, 5×10^{-3} M)

Synthesis of phenazine derivatives 4b,c

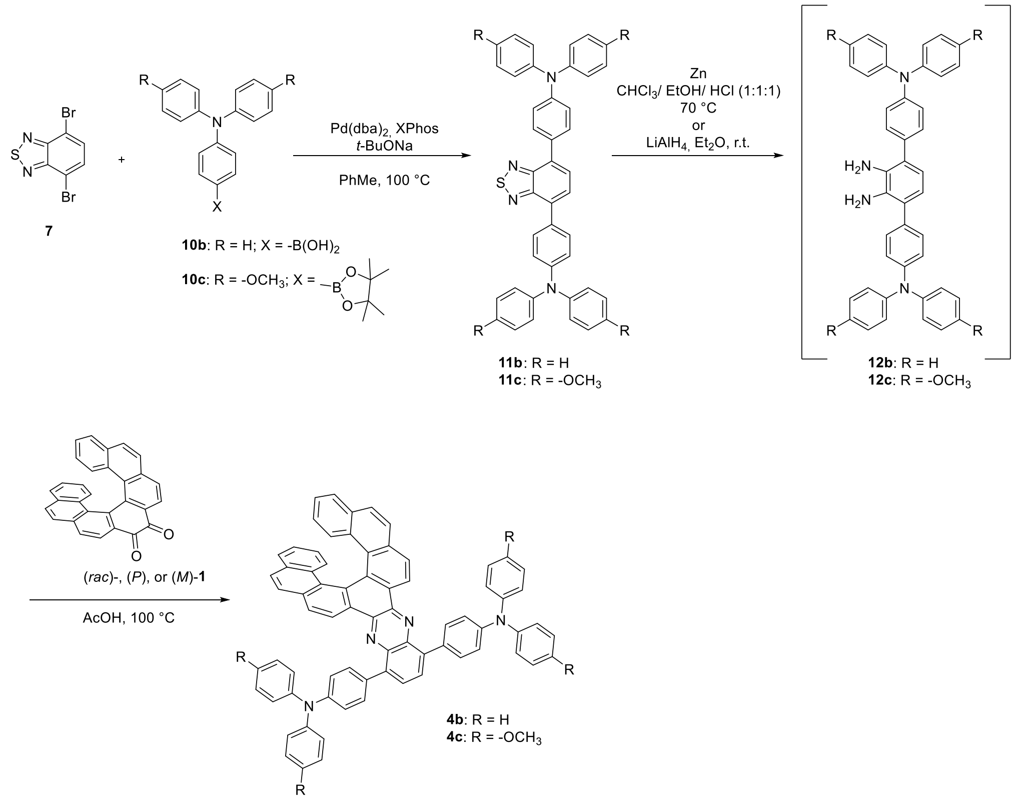

Scheme S4 *Synthesis of phenazine derivatives* ***4b,c***

4,4'-(Benzo[*c*][1,2,5]thiadiazole-4,7-diyl)bis(*N*,*N*-diphenylaniline) (11b)

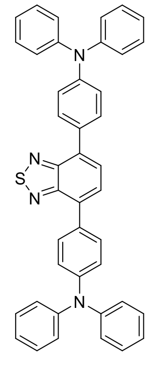

11b

The *GP1A* was followed with 100 mg (0.345 mmol) of **7**, 249 mg (0.862 mmol) of **10b**, 83.0 mg (0.862 mmol) of *t*-BuONa, 33.0 mg (0.069 mmol) of XPhos, and 20.0 mg (0.0345 mmol) of Pd(dba)$_2$. Column chromatography using PE/EtOAc (9:1) provided 109 mg (0.175 mmol, 51%) of **11b** as a bright orange powder.

^{1}H NMR (400 MHz, CDCl$_3$) δ 7.88 (d, *J* = 8 Hz, 4H), 7.74 (s, 2H), 7.29 (t, *J* = 8Hz, 8H), 7.20 (t, *J* = 8 Hz, 12H), 7.06 (t, *J* = 8 Hz, 4H).

R_f = 0.19 (PE/EtOAc 30:1).

NMR spectrum is in accordance with published data[4].

4,4'-(Benzo[*c*][1,2,5]thiadiazole-4,7-diyl)bis(*N*,*N*-bis(4-methoxyphenyl)aniline) (11c)

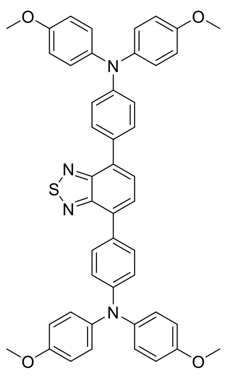

11c

The *GP1A* was followed with 500 mg (1.70 mmol) of **7**, 1.48 mg (4.25 mmol) of **10c**, 489 mg (5.10 mmol) of *t*-BuONa, 81 mg (0.17 mmol) of XPhos, and 49 mg (0.085 mmol) of Pd(dba)$_2$. Column chromatography using PE/EtOAc (9:1) provided 354 mg (0.477 mmol, 28%) of **11c** as a dark orange solid.

^{1}H NMR (400 MHz, CDCl$_3$) δ 7.81 (d, *J* = 8.4 Hz, 4H), 7.69 (s, 2H), 7.14 (d, *J* = 8.4 Hz, 8H), 7.05 (d, *J* = 8.3 Hz, 4-H), 6.86 (d, *J* = 8.4 Hz, 8H), 3.81 (s, 12H).

R_f = 0.32 (PE/EtOAc 4:1).

NMR spectrum is in accordance with published data[5].

(*rac*)-4,4'-(Diphenanthro[3,4-*a*:4',3'-*c*]phenazine-16,21-diyl)bis(*N*,*N*-diphenylaniline) (4b)

At first, crude diamine **12b** was obtained by following GP3 with 233 mg (0.375 mmol) of **11b** and 1 000 mg (15.38 mmol) of zinc powder. Then, the GP4 was followed with crude **12b** (0.374 mmol), 100 mg (0.245 mmol) of (*rac*)-**1**, and 25 mL of degassed AcOH (0.01M). Column chromatography using PE/$CHCl_3$ (90:10 to 50:50) provided 168 mg (0.174 mmol, 73%) of (*rac*)-**4b** as an orange-red solid.

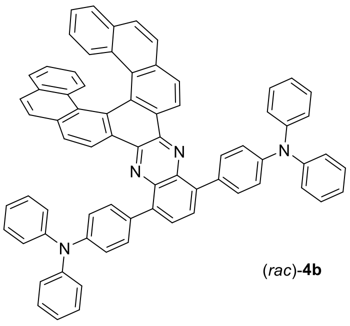

(*rac*)-**4b**

(*P*)-4,4'-(Diphenanthro[3,4-*a*:4',3'-*c*]phenazine-16,21-diyl)bis(*N*,*N*-diphenylaniline) (4b)

At first, crude diamine **12b** was obtained by following GP3 with 23 mg (0.037 mmol) of **11b** and 99.0 mg (1.52 mmol) of zinc powder. Then, the GP4 was followed with crude **12b** (0.037 mmol), 10 mg (0.024 mmol) of (*P*)-**1**, and 2 mL of degassed AcOH (0.01M). Column chromatography using PE/$CHCl_3$ (90:10 to 50:50) provided 16 mg (0.017 mmol, 70%) of (*P*)-**4b** as an orange-red solid.

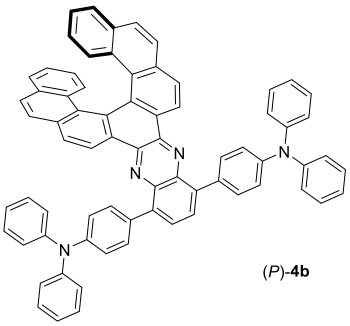

(*P*)-**4b**

(*M*)-4,4'-(Diphenanthro[3,4-*a*:4',3'-*c*]phenazine-16,21-diyl)bis(*N*,*N*-diphenylaniline) (4b)

At first, crude diamine **12b** was obtained by following GP3 with 23 mg (0.037 mmol) of **11b** and 99.0 mg (1.52 mmol) of zinc powder. Then, the GP4 was followed with crude **12b** (0.037 mmol), 10 mg (0.024 mmol) of (*M*)-**1**, and 2 mL of degassed AcOH (0.01M). Column chromatography using PE/$CHCl_3$ (90:10 to 50:50) provided 20 mg (0.021 mmol, 87%) of (*M*)-**4b** as an orange-red solid.

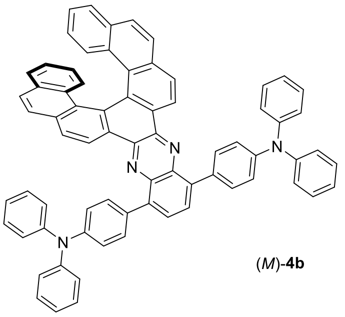

(*M*)-**4b**

^{1}H NMR (400 MHz, $CDCl_3$) δ 9.31 (d, *J* = 8.2 Hz, 2H), 8.09 (d, *J* = 8.3 Hz, 2H), 8.05 (s, 2H), 8.02 – 7.98 (m, 4H), 7.72 (d, *J* = 8.6 Hz, 2H), 7.48 (d, *J* = 8.6 Hz, 2H), 7.41 – 7.31 (m, 20H), 7.30 – 7.27 (m, 2H), 7.14 – 7.09 (m, 4H), 6.98 – 6.90 (m, 4H), 6.43 (ddd, *J* = 8.4, 7.0, 1.4 Hz, 2H).

^{13}C {^{1}H} NMR (101 MHz, $CDCl_3$) δ 147.8 (4C), 147.4 (2C), 141.4 (2C), 140.2 (2C), 139.0 (2C), 133.4 (2C), 132.8 (2C), 132.0 (4C), 131.7 (2C), 130.1 (2C), 129.4 (2C), 129.3 (8C), 192.2 (4C), 128.6 (2C), 128.5 (2C), 128.1 (2C), 126.6 (2C), 125.7 (2C), 125.4 (2C), 124.9 (2C), 124.7 (8C), 123.6 (2C), 123.1 (2C), 123.02 (4C), 122.99 (4C).

R_f = 0.21 (PE/$CHCl_3$ 9:1).

HRMS (APCI/QTOF) *m/z* [M + H]$^+$ calculated for $[C_{72}H_{47}N_4]^+$ 967.3795 ; found 967.3791 (100%).

Melting point: > 300.0 °C.

(*rac*)-4,4'-(Diphenanthro[3,4-*a*:4',3'-*c*]phenazine-16,21-diyl)bis(*N*,*N*-bis(4-methoxyphenyl)aniline) (4c)

At first, crude diamine **12c** was obtained by following GP2 with 100 mg (0.135 mmol) of **11c** and 1.35 mL of solution of $LiAlH_4$ in THF (1M, 1.35 mmol). Then, the GP4 was followed with crude **12c** (0.0674 mmol), 27.5 mg (0.0674 mmol) of (*rac*)-**1**, and 7 mL of degassed AcOH (0.01M). Reaction mixture was diluted with EtOH and filtered, which provided 45.2 mg (0.0416 mmol, 62%) of (*rac*)-**4c** as a dark orange solid.

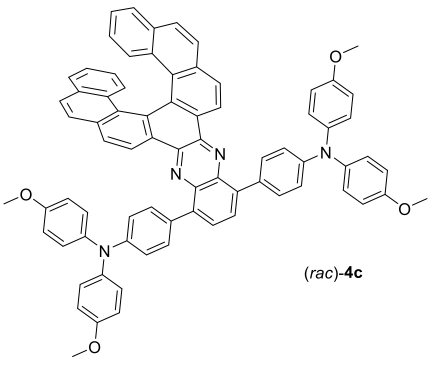

(*rac*)-**4c**

(*P*)-4,4'-(Diphenanthro[3,4-*a*:4',3'-*c*]phenazine-16,21-diyl)bis(*N*,*N*-bis(4-methoxyphenyl)aniline) (4c)

At first, crude diamine **12c** was obtained by following GP2 with 36 mg (0.049 mmol) of **11c** and 0.49 mL of solution of $LiAlH_4$ in THF (1M, 0.49 mmol). Then, the GP4 was followed with crude **12c** (0.049 mmol), 10 mg (0.024 mmol) of (*P*)-**1**, and 2 mL of degassed AcOH (0.01M). Column chromatography using PE/$CHCl_3$ (100:0 to 0:100) provided 24 mg (0.022 mmol, 90%) of (*P*)-**4c** as a red solid.

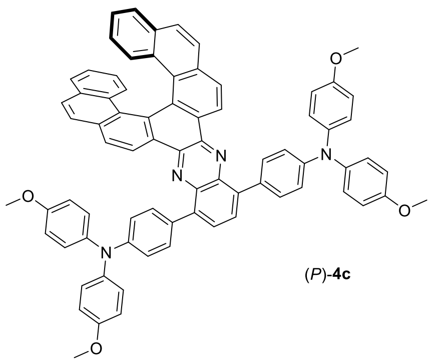

(*P*)-**4c**

(*M*)-4,4'-(Diphenanthro[3,4-*a*:4',3'-*c*]phenazine-16,21-diyl)bis(*N*,*N*-bis(4-methoxyphenyl)aniline) (4c)

At first, crude diamine **12c** was obtained by following GP2 with 36 mg (0.049 mmol) of **11c** and 0.49 mL of solution of $LiAlH_4$ in THF (1M, 0.49 mmol). Then, the GP4 was followed with crude **12c** (0.049 mmol), 10 mg (0.024 mmol) of (*M*)-**1**, and 2 mL of degassed AcOH (0.01M). Column chromatography using PE/$CHCl_3$ (100:0 to 0:100) provided 23 mg (0.021 mmol, 86%) of (*M*)-**4c** as a red solid.

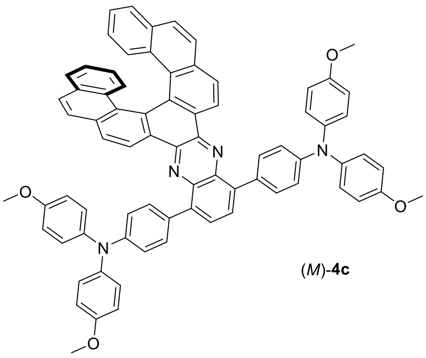

(*M*)-**4c**

^{1}H NMR (400 MHz, CD_2Cl_2) δ 9.27 (d, *J* = 8.3 Hz, 2H), 8.09 (d, *J* = 8.2 Hz, 2H), 7.98 (s, 2H), 7.95 – 7.89 (m, 4H), 7.72 (d, *J* = 8.6 Hz, 2H), 7.47 (d, *J* = 8.6 Hz, 2H), 7.30 – 7.22 (m, 10H), 7.22 – 7.16 (m, 4H), 7.02 – 6.89 (m, 12H), 6.46 – 6.39 (m, 2H), 3.84 (s, 12H).

^{13}C {^{1}H} NMR (101 MHz, CD_2Cl_2) δ 156.8 (4C), 149.0 (2C), 141.8 (2C), 141.4 (4C), 140.8 (2C), 139.3 (2C), 134.0 (2C), 132.31 (2C), 132.30 (4C), 131.2 (2C), 130.7 (2C), 130.0 (2C), 129.70 (2C), 129.67 (2C), 129.08 (2C), 129.04 (2C), 128.6 (2C), 127.6 (8C), 127.1 (2C), 126.3 (2C), 125.9 (2C), 125.4 (2C), 124.2 (2C), 123.5 (2C), 120.0 (4C), 115.3 (8C), 56.1 (4C).

R_f = 0.46 ($CHCl_3$).

HRMS (APCI/QTOF) *m/z* [M + H]$^+$ calculated for $[C_{76}H_{55}N_4O_4]^+$ 1087.4217; found 1087.4207 (100%).

Melting point: 195.0-198.0 °C.

Synthesis of benzo[*b*]phenazine derivatives 5b,c

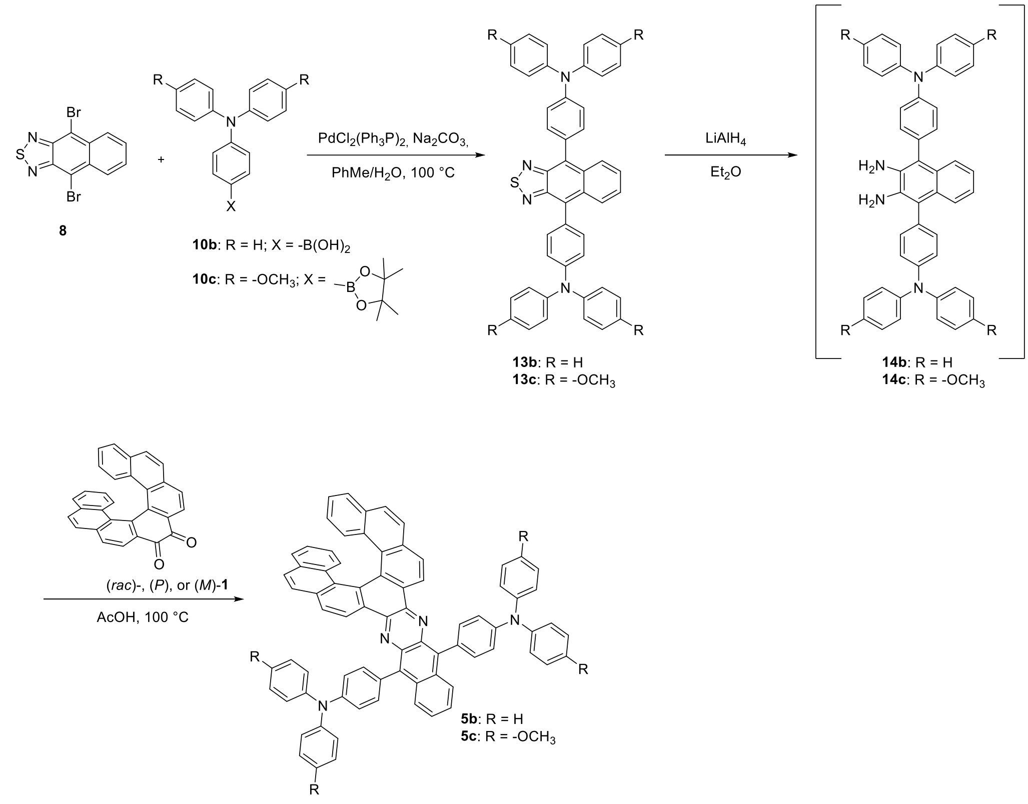

Scheme S5 *Synthesis of benzo[b]phenazine derivatives* ***5b,c***

4,4'-(Naphtho[2,3-*c*][1,2,5]thiadiazole-4,9-diyl)bis(*N*,*N*-diphenylaniline) (13b)

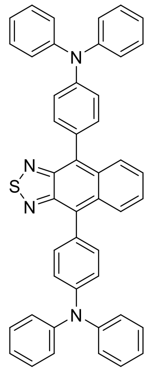

13b

The *GP1B* was followed with 200 mg (0.581 mmol) of **8**, 420 mg (1.453 mmol) of **10b**, 246 mg (2.325 mmol) of Na_2CO_3, and 82 mg (0.116 mmol) of $PdCl_2(Ph_3P)_2$. Column chromatography using $CHCl_3$ provided 290 mg (0.431 mmol, 74%) of **13b** as a purple solid.

^{1}H NMR (400 MHz, CD_2Cl_2) δ 8.13 (dd, *J* = 7.0, 3.2 Hz, 2H), 7.54 (d, *J* = 8.6 Hz, 4H), 7.42 – 7.30 (m, 10H), 7.30 – 7.22 (m, 12H), 7.11 (tt, *J* = 7.2, 1.2 Hz, 4H).

R_f = 0.40 ($CHCl_3$).

NMR spectrum is in accordance with published data[6].

4,4'-(Naphtho[2,3-*c*][1,2,5]thiadiazole-4,9-diyl)bis(*N*,*N*-bis(4-methoxyphenylaniline) (13c)

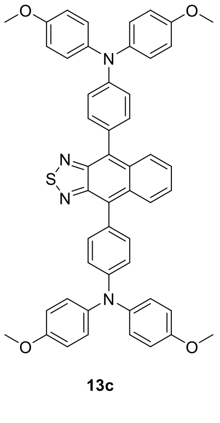

13c

The *GP1B* was followed with 300 mg (0.872 mmol) of **8**, 940 mg (2.180 mmol) of **10c**, 370 mg (3.49 mmol) of Na_2CO_3, and 122 mg (0.174 mmol) of $PdCl_2(Ph_3P)_2$. Column chromatography using $CHCl_3$ provided 627 mg (0.791 mmol, 91%) of **13c** as a purple solid.

^{1}H NMR (400 MHz, $CDCl_3$) δ 8.13 (dd, *J* = 7.1, 3.3 Hz, 2H), 7.47 (d, *J* = 8.7 Hz, 4H), 7.34 (dd, *J* = 7.0, 3.2 Hz, 2H), 7.27 – 7.19 (m, 10H), 7.12 (d, *J* = 8.7 Hz, 4H), 6.90 (d, *J* = 8.9 Hz, 6H), 3.82 (s, 12H).

^{13}C {^{1}H} NMR (101 MHz, $CDCl_3$) δ 156.4 (4C), 151.8 (2C), 148.8 (2C), 140.7 (4C), 132.2 (2C), 132.2 (4C), 129.92 (2C), 129.89 (2C), 127.9 (2C), 127.5 (8C), 126.1 (2C), 119.1 (4C), 115.0 (8C), 55.7 (4C).

R_f = 0.29 ($CHCl_3$).

HRMS (APCI/QTOF) *m/z* [M + H]$^+$ calculated for [$C_{50}H_{41}N_4O_4S$]$^+$ 793.2843 ; found 793.2819 (100%).

Melting point: 153.4-155.3 °C (EtOH).

(*rac*)-4,4'-(Benzo[*i*]diphenanthro[3,4-*a*:4',3'-*c*]phenazine-16,21-diyl)bis(*N*,*N*-diphenylaniline) (5b)

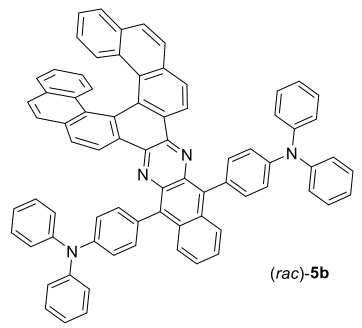

(*rac*)-**5b**

Pathway 1: At first, crude diamine **14b** was obtained by following GP2 with 68 mg (0.101 mmol) of **13b** and 1 mL of solution of $LiAlH_4$ in THF (1M, 1.01 mmol). Then, the GP4 was followed with crude **14b** (0.101 mmol), 21 mg (0.051 mmol) of (*rac*)-**1**, and 2 mL of degassed AcOH (0.02M). Column chromatography using PE/$CHCl_3$ (50:50) provided 38 mg (0.037 mmol, 74%) of (*rac*)-**5b** as a red solid.

Pathway 2: A microwave vial was charged with 20 mg of (*rac*)-**3** (0.03 mmol) and 41 mg of (*S*,*P*)/(*M*,*R*)-**3**. The vial was capped with PTFE septa and a 1.8 mL mixture of AcOH and H_2O (4:1) was added (0.2M). The reaction mixture was reacted in a microwave reactor for 2 hours at 150° C. After completion of the reaction, the mixture was extracted with DCM, dried over anhydrous $MgSO_4$, filtered, and the solvent was evaporated under reduced pressure. The column chromatography using PE/$CHCl_3$(50:50) provided 26 mg (*rac*)-**5b** (0.026 mmol, 84%) as a red solid.

(*P*)-4,4'-(Benzo[*i*]diphenanthro[3,4-*a*:4',3'-*c*]phenazine-16,21-diyl)bis(*N*,*N*-diphenylaniline) (5b)

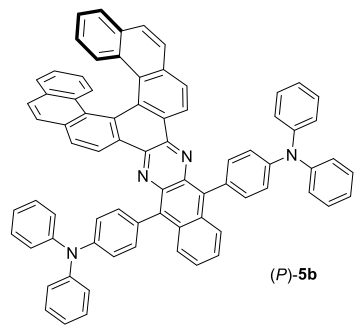

(*P*)-**5b**

At first, crude diamine **14b** was obtained by following GP2 with 49 mg (0.073 mmol) of **13b** and 0.73 mL of solution of $LiAlH_4$ in THF (1M, 0.734 mmol). Then, the GP4 was followed with crude **14b** (0.073 mmol), 15 mg (0.037 mmol) of (*P*)-**1**, and 2 mL of degassed AcOH (0.02M). Column chromatography using PE/$CHCl_3$ (50:50) provided 30 mg (0.029 mmol, 80%) of (*P*)-**5b** as a red solid.

(*M*)-4,4'-(Benzo[*i*]diphenanthro[3,4-*a*:4',3'-*c*]phenazine-16,21-diyl)bis(*N*,*N*-diphenylaniline) (5b)

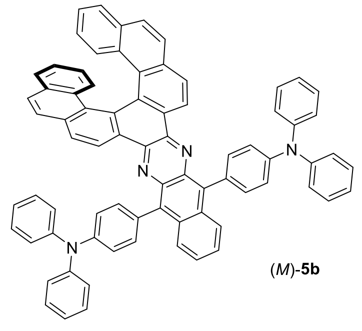

(*M*)-**5b**

At first, crude diamine **14b** was obtained by following GP2 with 66 mg (0.098 mmol) of **13b** and 1 mL of solution of $LiAlH_4$ in THF (1M, 1.01 mmol). Then, the GP4 was followed with crude **14b** (0.098 mmol), 20 mg (0.049 mmol) of (*M*)-**1**, and 2 mL of degassed AcOH (0.01M). Column chromatography using PE/$CHCl_3$ (50:50) provided 45 mg (0.044 mmol, 90%) of (*M*)-**5b** as a red solid.

^{1}H NMR (400 MHz, $CDCl_3$) δ 9.12 (d, *J* = 8.2 Hz, 2H), 8.38 (dd, *J* = 6.8, 3.3 Hz, 2H), 8.04 (d, *J* = 8.3 Hz, 2H), 7.80 – 7.72 (m, 2H), 7.68 (dd, *J* = 11.2, 8.4 Hz, 4H), 7.63 – 7.54 (m, 2H), 7.54 – 7.37 (m, 22H), 7.29 – 7.25 (m, 2H), 7.20 – 7.08 (m, 4H), 6.99 – 6.85 (m, 4H), 6.41 (ddd, *J* = 8.3, 6.9, 1.3 Hz, 2H).

^{13}C {^{1}H} NMR (101 MHz, $CDCl_3$) δ 148.2 (4C), 147.3 (2C), 142.9 (2C), 137.8 (2C), 137.0 (2C), 134.0 (2C), 133.8 (2C), 133.7 (2C), 132.3 (2C), 131.93 (2C), 131.87 (2C), 130.5 (2C), 129.63 (2C), 129.58 (4C), 129.53 (8C), 128.6 (2C), 128.3 (2C), 127.8 (2C), 126.8 (2C), 126.3 (2C), 125.8 (2C), 125.6 (2C), 125.2 (2C), 124.6 (8C), 123.7 (2C), 123.5 (4C), 123.4 (2C), 123.0 (4C).

R_f = 0.75 (PE/$CHCl_3$ 33:67).

HRMS (APCI/QTOF) *m/z* [M + H]$^+$ calculated for $[C_{76}H_{49}N_4]^+$ 1017.3952 ; found 1017.3930 (100%).

Melting point: 235.0-242.0 °C.

(*rac*)-4,4'-(benzo[*i*]diphenanthro[3,4-*a*:4',3'-*c*]phenazine-16,21-diyl)bis(*N*,*N*-bis(4-methoxyphenyl)aniline) (5c)

At first, crude diamine **14c** was obtained by following GP2 with 75 mg (0.095 mmol) of **13c** and 0.95 mL of solution of $LiAlH_4$ in THF (1M, 0.946 mmol). Then, the GP4 was followed with crude **14c** (0.095 mmol), 19 mg (0.047 mmol) of (*rac*)-**1**, and 2 mL of degassed AcOH (0.02M). Column chromatography using PE/$CHCl_3$/EtOAc (48:48:4) provided 43 mg (0.038 mmol, 80%) of (*rac*)-**5c** as a dark brown solid.

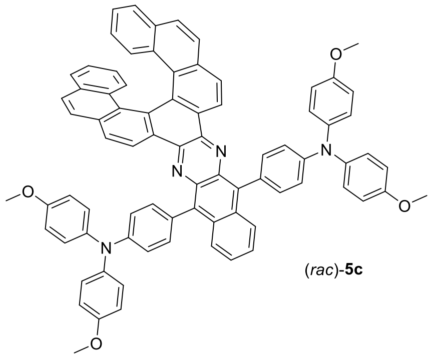

(*rac*)-**5c**

(*P*)-4,4'-(Benzo[*i*]diphenanthro[3,4-*a*:4',3'-*c*]phenazine-16,21-diyl)bis(*N*,*N*-bis(4-methoxyphenyl)aniline) (5c)

At first, crude diamine **14c** was obtained by following GP2 with 47 mg (0.059 mmol) of **13c** and 0.58 mL of solution of $LiAlH_4$ in THF (1M, 0.588 mmol). Then, the GP4 was followed with crude **14c** (0.059 mmol), 12 mg (0.029 mmol) of (*P*)-**1**, and 2 mL of degassed AcOH (0.02M). Column chromatography using PE/$CHCl_3$/EtOAc (48:48:4) provided 28 mg (0.025 mmol, 84%) of (*P*)-**5c** as a dark brown solid.

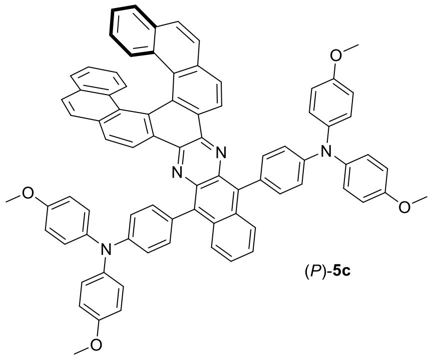

(*P*)-**5c**

(*M*)-4,4'-(Benzo[*i*]diphenanthro[3,4-*a*:4',3'-*c*]phenazine-16,21-diyl)bis(*N*,*N*-bis(4-methoxyphenyl)aniline) (5c)

At first, crude diamine **14c** was obtained by following GP2 with 78 mg (0.098 mmol) of **13c** and 0.98 mL of solution of $LiAlH_4$ in THF (1M, 0.979 mmol). Then, the GP4 was followed with crude **14c** (0.098 mmol), 20 mg (0.049 mmol) of (*M*)-**1**, and 2 mL of degassed AcOH (0.02M). Column chromatography using PE/$CHCl_3$/EtOAc (48:48:4) provided 43 mg (0.038 mmol, 77%) of (*M*)-**5c** as a dark brown solid.

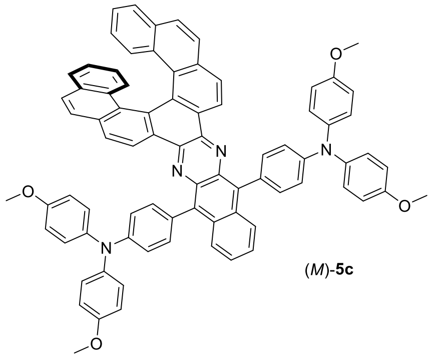

(*M*)-**5c**

^{1}H NMR (400 MHz, $CDCl_3$) δ 9.14 (d, *J* = 8.2 Hz, 2H), 8.38 (dd, *J* = 6.9, 3.3 Hz, 2H), 8.04 (d, *J* = 8.3 Hz, 2H), 7.74 – 7.65 (m, 4H), 7.59 (d, *J* = 7.9 Hz, 2H), 7.55 (dd, *J* = 6.9, 3.2 Hz, 2H), 7.45 (d, *J* = 8.6 Hz, 2H), 7.40 – 7.27 (m, 14H), 7.04 – 6.87 (m, 12H), 6.41 (ddd, *J* = 8.3, 7.0, 1.4 Hz, 2H), 3.88 (s, 12H).

^{13}C {^{1}H} NMR (101 MHz, $CDCl_3$) δ 156.0 (4C), 148.2 (2C), 142.8 (2C), 141.5 (4C), 137.8 (2C), 137.0 (2C), 133.9 (2C), 133.63 (2C), 133.56 (2C), 132.5 (2C), 131.9 (2C), 130.6 (2C), 129.8 (2C), 129.59 (2C), 129.57 (2C), 129.54 (2C), 128.6 (2C), 128.3 (2C), 127.9 (2C), 126.8 (8C), 126.1 (2C), 125.9 (2C), 125.5 (2C), 125.2 (2C), 123.7 (2C), 123.5 (2C), 120.3 (4C), 115.0 (8C), 55.7 (4C).

We were unable to appropriately assign one of the carbon signals corresponding to 4 carbons, instead of 2. The total number of individual signals is however correct.

R_f = 0.54 ($CHCl_3$).

HRMS (APCI/QTOF) *m/z* $[M + H]^+$ calculated for $[C_{80}H_{57}N_4O_4]^+$ 1137.4374 ; found 1137.4363 (100%).

Melting point: 166.0-170.0 °C.

Synthesis of thieno[3,4-*b*]quinoxaline derivatives 6b,c

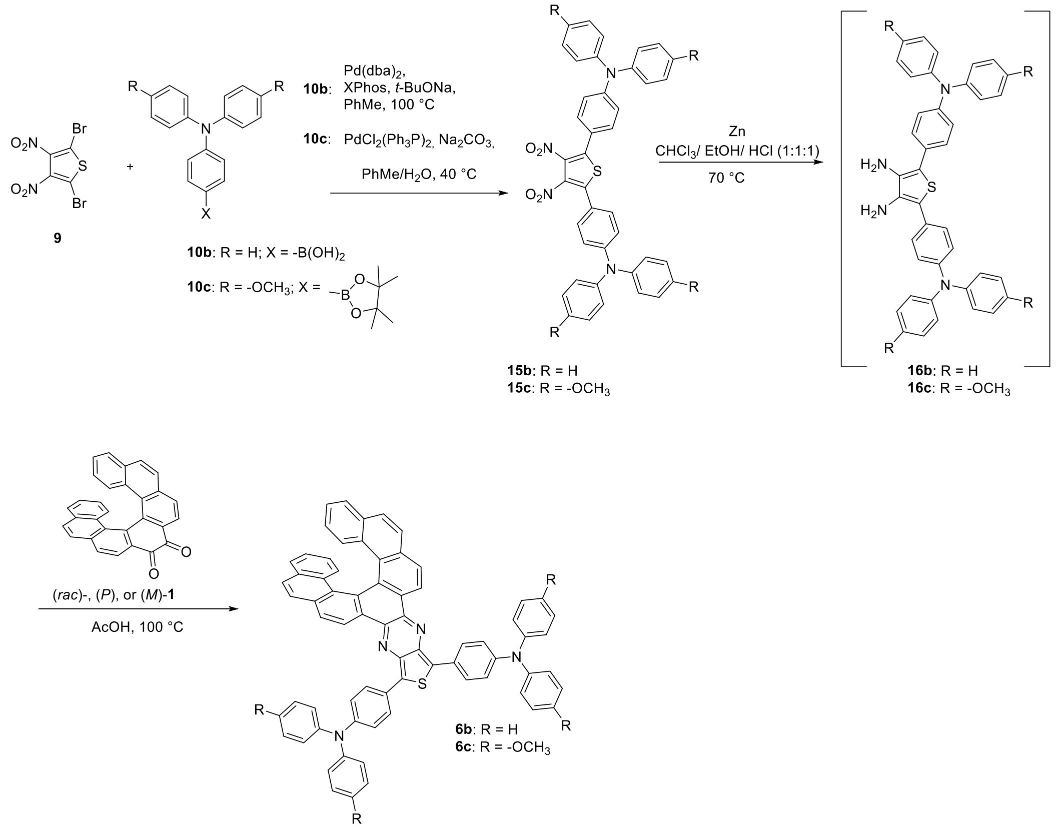

Scheme S6 *Synthesis of thieno[3,4-b]quinoxaline derivatives* ***6b,c***

4,4'-(3,4-Dinitrothiophene-2,5-diyl)bis(*N*,*N*-diphenylaniline) (15b)

The *GP1A* was followed with 50 mg (0.152 mmol) of **9**, 109 mg (0.337 mmol) of **10b**, 58 mg (0.603 mmol) of *t*-BuONa, 8.7 mg (0.015 mmol) of $Pd(dba)_2$ and 14 mg (0.03 mmol) of XPhos. Column chromatography using PE/EtOAc (90:10) provided 41 mg (0.062 mmol, 41%) of **15b** as a bright orange powder.

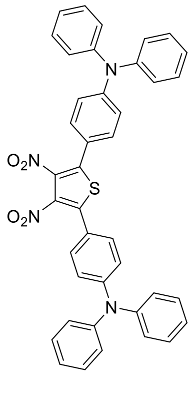

15b

^{1}H NMR (400 MHz, $CDCl_3$)δ 7.36 – 7.29 (m, 12H), 7.19 – 7.10 (m, 12H), 7.07 – 7.02 (m, 4H).

R_f = 0.57 (PE/EtOAc, 8:1).

NMR spectrum is in accordance with published data[7].

4,4'-(3,4-Dinitrothiophene-2,5-diyl)bis(*N*,*N*-bis(4-methoxyphenyl)aniline) (15c)

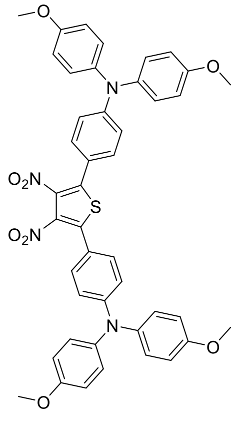

15c

The *GP1B* was followed with 50 mg (0.151 mmol) of **9**, 163 mg (0.38 mmol) of **10c**, 63 mg (0.60 mmol) of Na_2CO_3, and 21 mg (0.03 mmol) of $PdCl_2(Ph_3P)_2$. Column chromatography using PE/EtOAc (5:1) provided 75 mg (0.096 mmol, 64%) of **15c** as an orange solid.

^{1}H NMR (400 MHz, $CDCl_3$) δ 7.29 – 7.26 (m, 4H), 7.14 – 7.09 (m, 8H), 6.90 – 6.85 (m, 12H), 3.81 (s, 12H).

^{13}C {^{1}H} NMR (101 MHz, $CDCl_3$) δ 157.1 (4C), 151.0 (2C), 140.6 (2C), 139.5 (4C), 136.0 (2C), 130.0 (4C), 127.9 (8C), 118.4 (2C), 118.1 (4C), 115.1 (8C), 55.7 (4C).

R_f = 0.23 (PE/EtOAc 5:1).

HRMS (APCI/QTOF) *m/z* [M + H]$^+$ calculated for $[C_{44}H_{37}N_4O_8S]^+$ 781.2327; found 781.2322 (100%).

Melting point: 224.3-229.4 °C.

(*rac*)-Diphenanthro[3,4-*f*:4',3'-*h*]thieno[3,4-*b*]quinoxaline (6a)

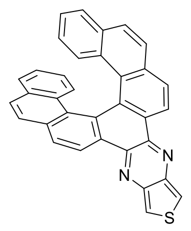

(*rac*)-**6a**

GP4 was followed with 50 mg (0.27 mmol) of 3,4-dinitrothiophene, 55 mg (0.13 mmol) of (*rac*)-**1**, and 5 mL of degassed AcOH (0.02M). Crystallization from DCM/MeCN (1:1) provided 44 mg (0.09 mmol, 68%) of (*rac*)-**6a** as a black powder.

NMR analysis of **6a** was attempted according to standard protocols. However, the compound exhibited insufficient solubility in the selected NMR solvents (e.g., $CDCl_3$, DMSO-d_6).

1 mmol scale reaction

GP4 was followed with 372 mg (2.00 mmol) of 3,4-dinitrothiophene, 408 mg (1.00 mmol) of (*rac*)-**1**, and 40 mL of degassed AcOH (0.03M). Crystallization from DCM/MeCN (1:1) provided 255 mg (0.52 mmol, 53%) of (*rac*)-**6a** as a black powder.

HRMS (APCI/QTOF) *m/z* [M]$^+$ calculated for $[C_{34}H_{18}N_2S]^+$ 486.1185; found 486.1189 (100%).

Melting point: > 300 °C.

(*rac*)-4,4'-(Diphenanthro[3,4-*f*:4',3'-*h*]thieno[3,4-*b*]quinoxaline-16,18-diyl)bis(*N*,*N*-diphenylaniline) (6b)

At first, crude diamine **16b** was obtained by following GP3 with 60 mg (0.10 mmol) of **15b** and 363 mg (5.56 mmol) of zinc powder. Then, the GP4 was followed with crude **16b** (0.1 mmol), 20 mg (0.05 mmol) of (*rac*)-**1**, and 10 mL of degassed AcOH (0.01M). Crystallization from DCM/MeCN (1:1) provided 42 mg (0.04 mmol, 89%) of (*rac*)-**6b** as a green powder.

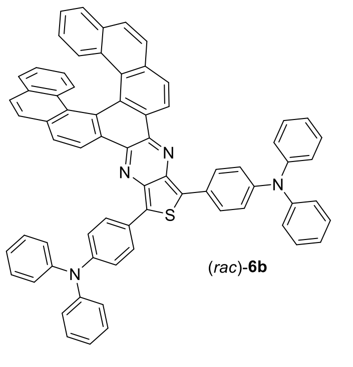

(*rac*)-**6b**

(*P*)-4,4'-(Diphenanthro[3,4-*f*:4',3'-*h*]thieno[3,4-*b*]quinoxaline-16,18-diyl)bis(*N*,*N*-diphenylaniline) (6b)

At first, crude diamine **16b** was obtained by following GP3 with 29 mg (0.044 mmol) of **15b** and 175 mg (2.68 mmol) of zinc powder. Then, the GP4 was followed with crude **16b** (0.044 mmol), 9 mg (0.022 mmol) of (*P*)-**1**, and 5 mL of degassed AcOH (0.01M). Crystallization from DCM/MeCN (1:1) provided 12 mg (0.012 mmol, 92%) of (*P*)-**6b** as a green powder.

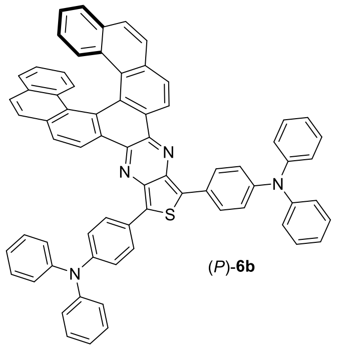

(*P*)-**6b**

(*M*)-4,4'-(Diphenanthro[3,4-*f*:4',3'-*h*]thieno[3,4-*b*]quinoxaline-16,18-diyl)bis(*N*,*N*-diphenylaniline) (6b)

At first, crude diamine **16b** was obtained by following GP3 with 29 mg (0.044 mmol) of **15b** and 175 mg (2.68 mmol) of zinc powder. Then, the GP4 was followed with crude **16b** (0.044 mmol), 9 mg (0.022 mmol) of (*M*)-**1**, and 5 mL of degassed AcOH (0.01M). Crystallization from DCM/MeCN (1:1) provided 9 mg (0.009 mmol, 69%) of (*M*)-**6b** as a green powder.

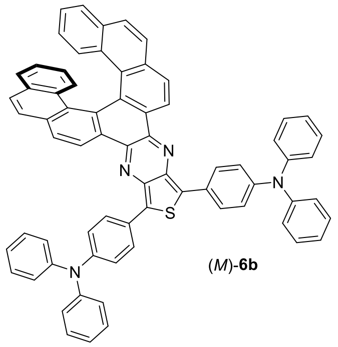

(*M*)-**6b**

^{1}H NMR (400 MHz, CD_2Cl_2) δ 9.28 (d, *J* = 8.2 Hz, 1H), 8.47 (s, 2H), 8.07 (d, *J* = 8.3 Hz, 1H), 7.70 (d, *J* = 8.6 Hz, 1H), 7.48 (d, *J* = 8.7 Hz, 1H), 7.41 – 7.09 (m, 13H), 7.00 – 6.91 (m, 2H), 6.44 (t, *J* = 7.7 Hz, 1H).
^{13}C {^{1}H} NMR spectrum was not obtained due to low solubility of the compound.

R_f = 0.62 (PE/EtOAc 1:1).

HRMS (APCI/QTOF) *m/z* [M + H]$^+$ calculated for $[C_{70}H_{45}N_4S]^+$ 973.3359 ; found 973.3349 (100%).

Melting point: > 300.0 °C.

(*rac*)-4,4'-(Diphenanthro[3,4-*f*:4',3'-*h*]thieno[3,4-*b*]quinoxaline-16,18-diyl)bis(*N*,*N*-(4-methoxy-phenyl)aniline) (6c)

At first, crude diamine **16c** was obtained by following GP3 with 38 mg (0.049 mmol) of **15c** and 195 mg (2.99 mmol) of zinc powder. Then, the GP4 was followed with crude **16c** (0.049 mmol), 10 mg (0.025 mmol) of (*rac*)-**1**, and 5 mL of degassed AcOH (0.01M). Crystallization from DCM/MeCN (1:1) provided 13 mg (0.012 mmol, 50%) of (*rac*)-**6c** as a dark green powder.

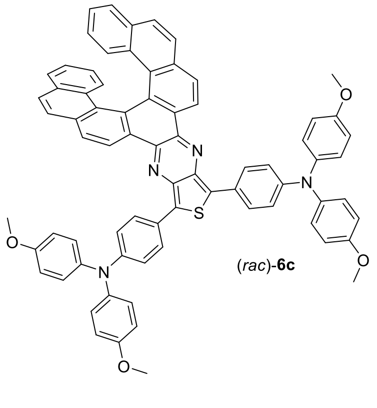

(*rac*)-**6c**

(*P*)-4,4'-(Diphenanthro[3,4-*f*:4',3'-*h*]thieno[3,4-*b*]quinoxaline-16,18-diyl)bis(*N*,*N*-(4-methoxyphenyl) aniline) (6c)

At first, crude diamine **16c** was obtained by following GP3 with 35 mg (0.045 mmol) of **15c** and 179 mg (2.74 mmol) of zinc powder. Then, the GP4 was followed with crude **16c** (0.045 mmol), 9 mg (0.023 mmol) of (*P*)-**1**, and 5 mL of degassed AcOH (0.01M). Crystallization from DCM/MeCN (1:1) provided 16 mg (0.015 mmol, 67%) of (*P*)-**6c** as a dark green powder.

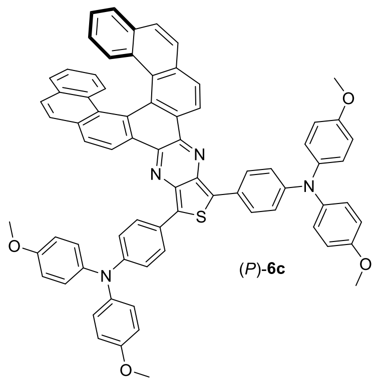

(*P*)-**6c**

(*M*)-4,4'-(Diphenanthro[3,4-*f*:4',3'-*h*]thieno[3,4-*b*]quinoxaline-16,18-diyl)bis(*N*,*N*-(4-methoxyphenyl)aniline) (6c)

At first, crude diamine **16c** was obtained by following GP3 with 35 mg (0.045 mmol) of **15c** and 179 mg (2.74 mmol) of zinc powder. Then, the GP4 was followed with crude **16c** (0.045 mmol), 9 mg (0.02 mmol) of (*M*)-**1**, and 5 mL of degassed AcOH (0.01M). Crystallization from DCM/MeCN (1:1) provided 18 mg (0.016 mmol, 75%) of (*M*)-**6c** as a dark green powder.

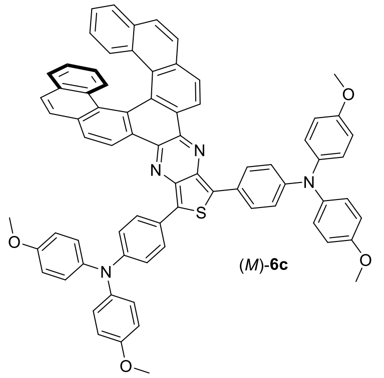

(*M*)-**6c**

^{1}H NMR (400 MHz, DMSO-d_6) δ 9.10 (s, 2H), 8.31 (s, 4H), 8.15 (s, 2H), 7.78 (s, 2H), 7.59 (d, *J* = 8.5 Hz, 2H), 7.41 – 7.35 (m, 2H), 7.21 – 7.15 (m, 8H), 7.04 – 6.95 (m, 14H), 6.81 (d, *J* = 8.0 Hz, 2H), 6.41 (s, 2H), 3.80 (s, 12H).

^{13}C {^{1}H} NMR spectrum was not obtained due to low solubility of the compound.

R_f = 0.67 (PE/EtOAc 2:1).

HRMS (APCI/QTOF) *m/z* $[M + H]^+$ calculated for $[C_{74}H_{53}N_4O_4S]^+$ 1093.3782 ; found 1093.3783 (100%).

Melting point: > 300.0 °C.

3. NMR spectra

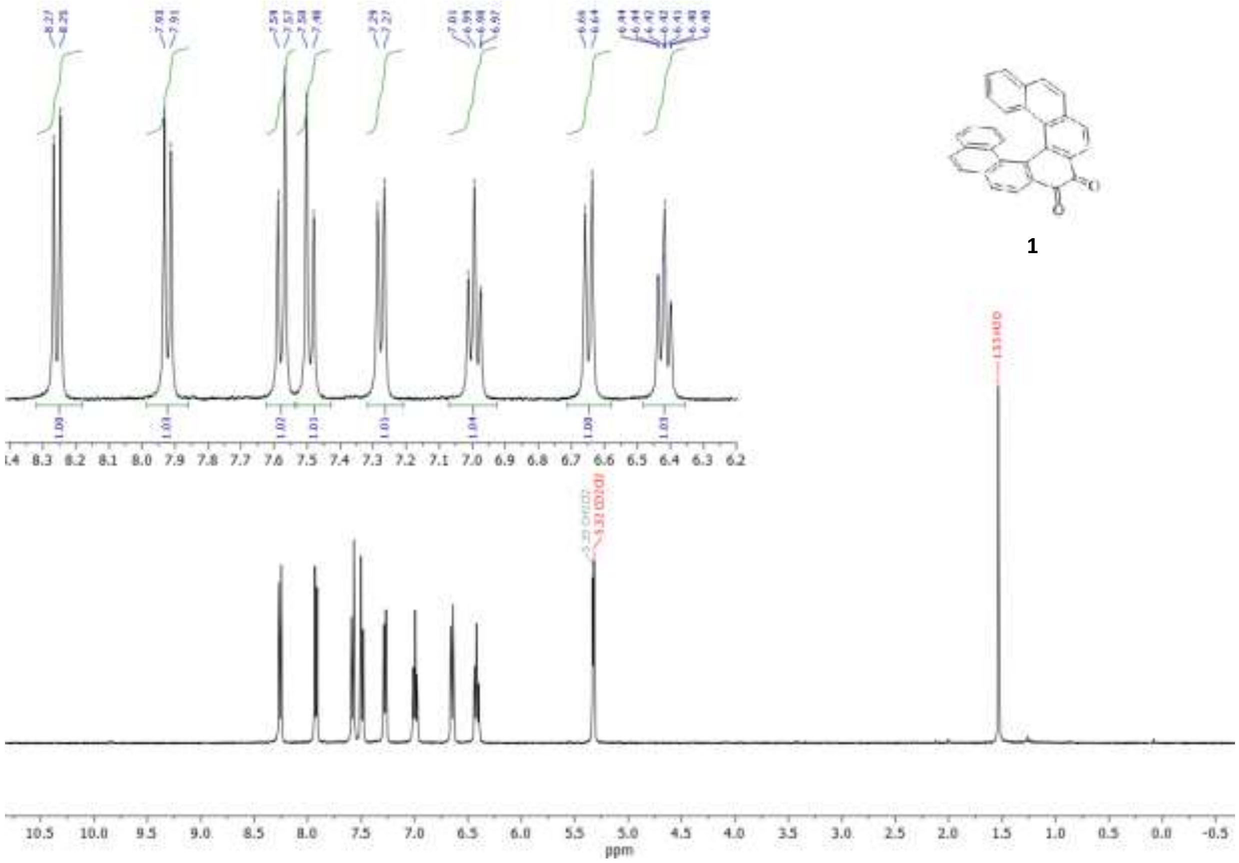

1

***Figure S1** ^{1}H NMR spectrum of **1** (400 MHz, CD_2Cl_2).*

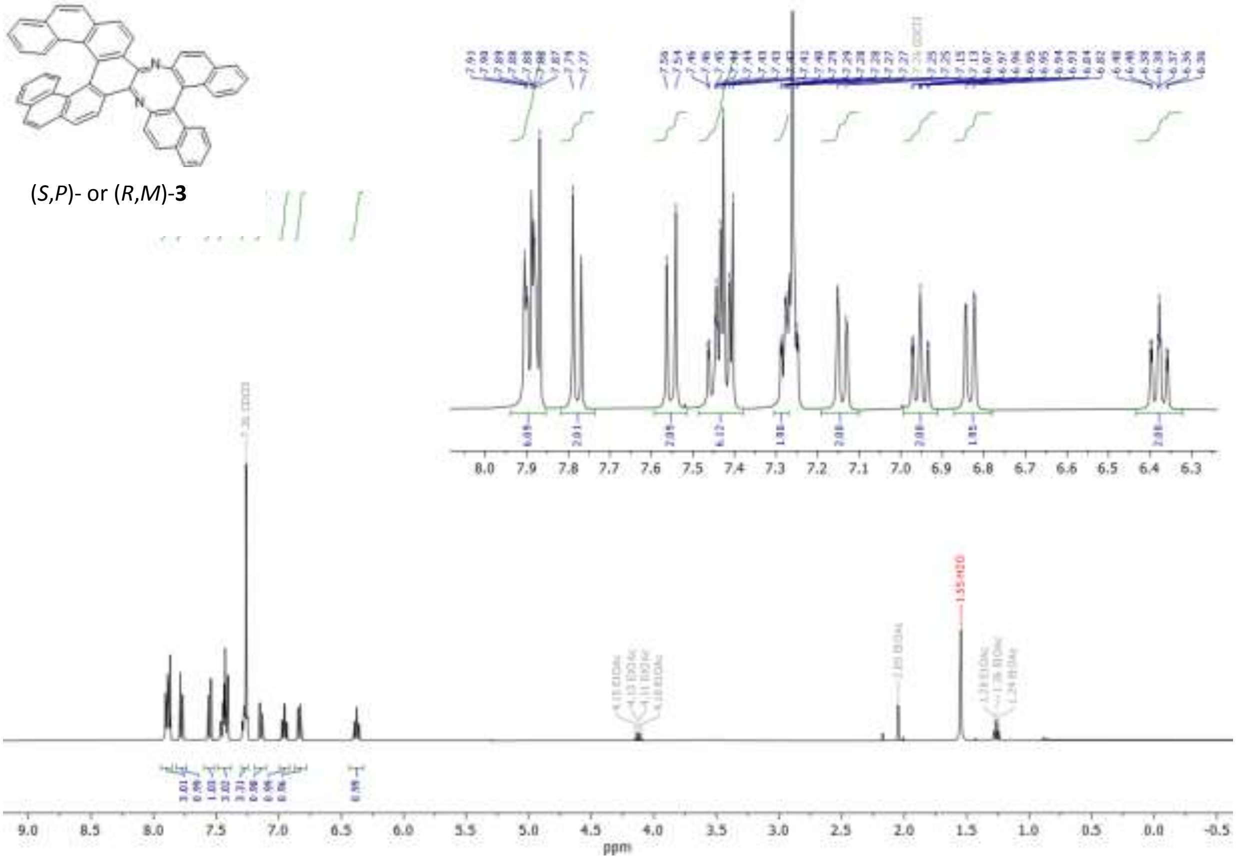

(*S*,*P*)- or (*R*,*M*)-**3**

Figure S2 ^{1}H *NMR spectrum of (S,P)/(R,M)-(**3**) (400 MHz,* $CDCl_3$*)*

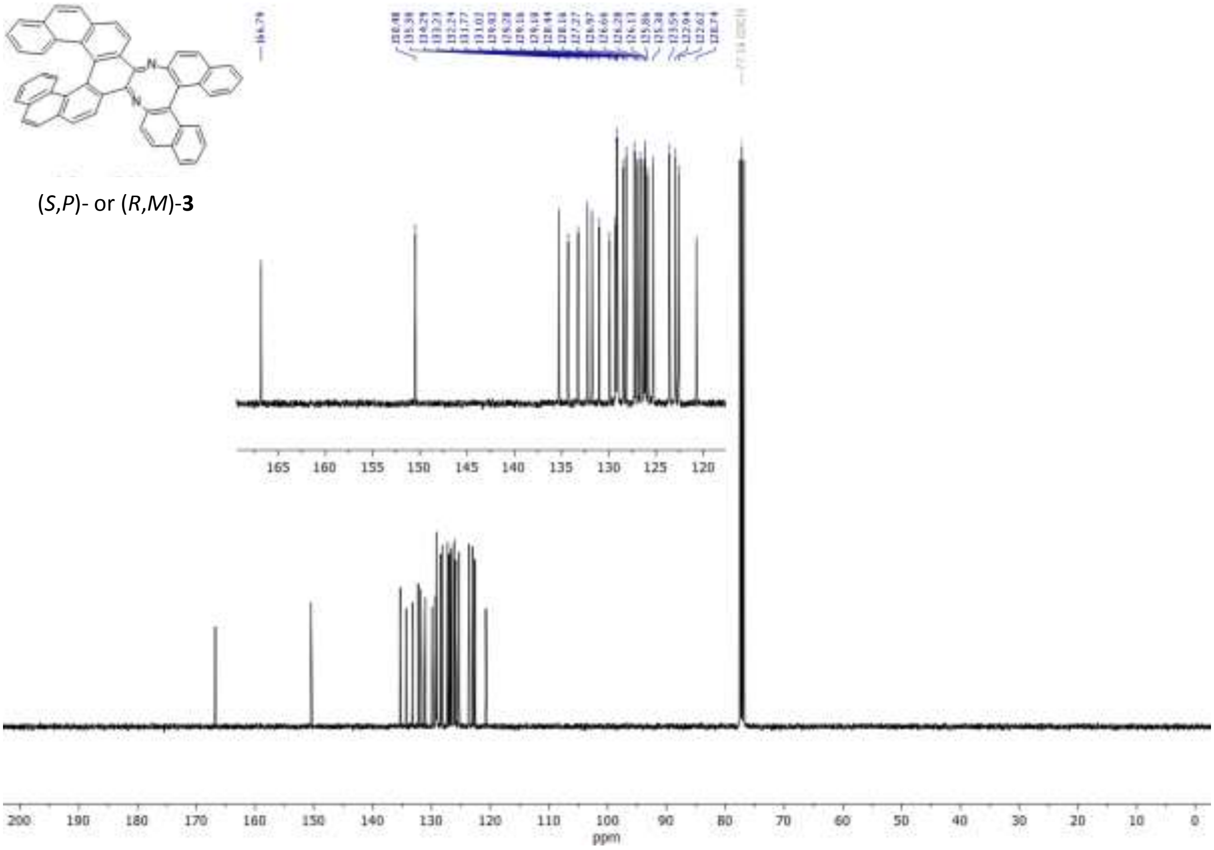

(*S*,*P*)- or (*R*,*M*)-**3**

Figure S3 $^{13}C\{^{1}H\}$ *NMR spectrum of (S,P)/(R,M)-(**3**) (101 MHz,* $CDCl_3$*)*

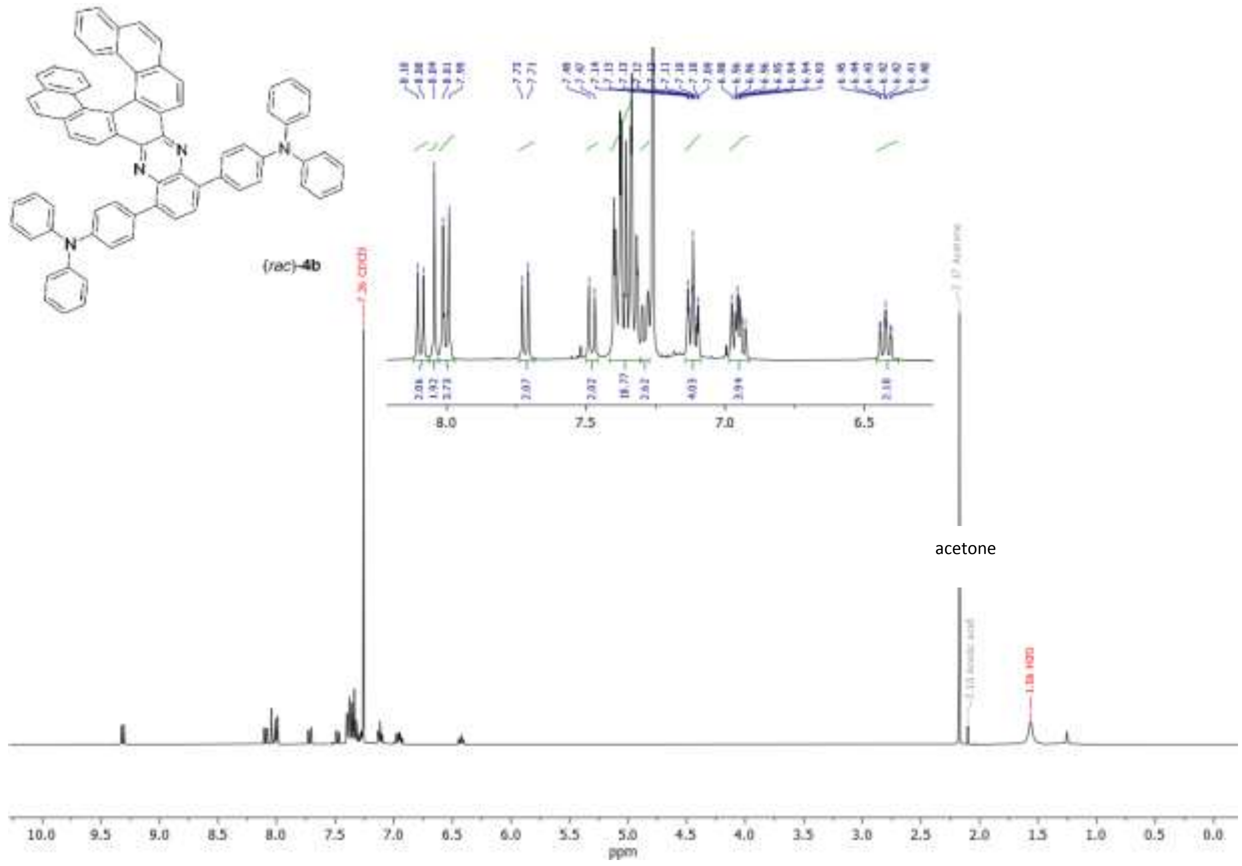

Figure S4 *^{1}H NMR spectrum of (**4b**) (400 MHz, $CDCl_3$)*

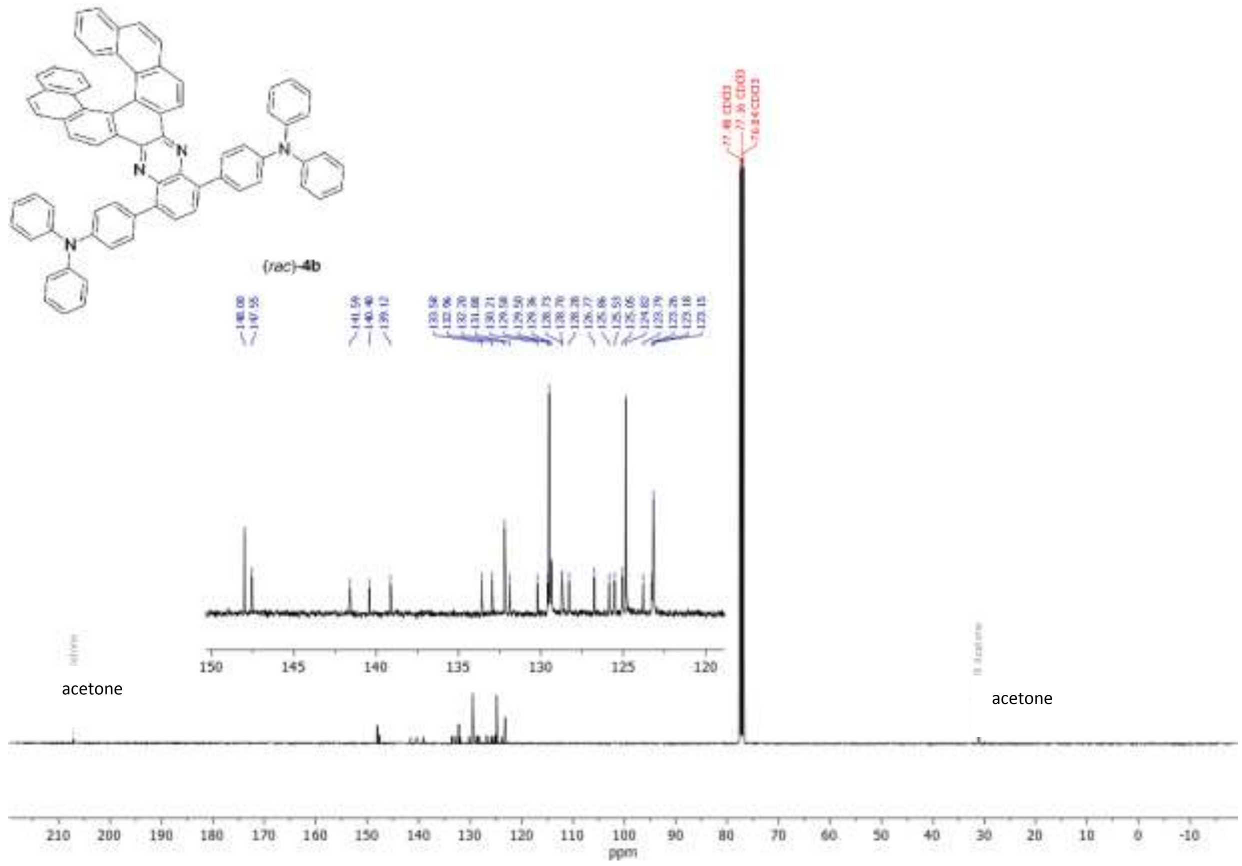

Figure S5 *^{13}C {^{1}H} NMR spectrum of (**4b**) (101 MHz, $CDCl_3$*

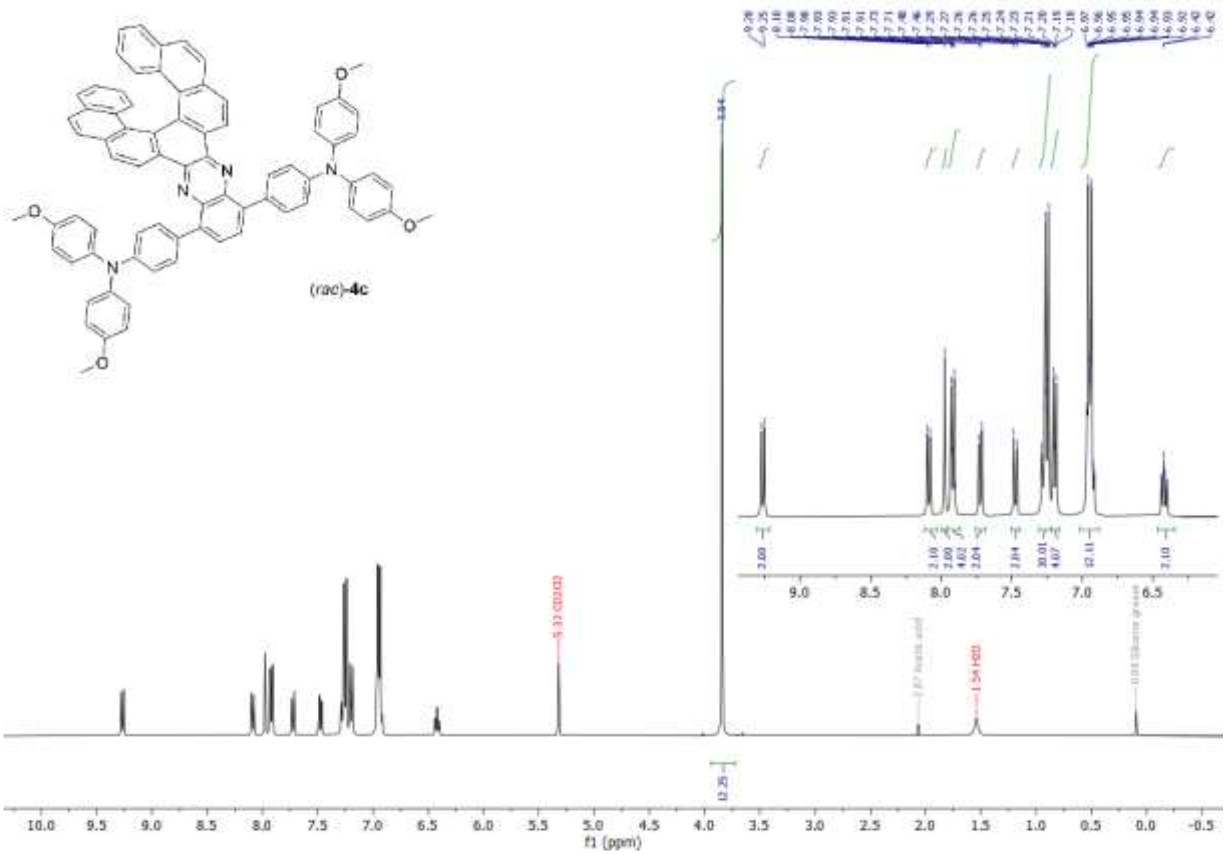

Figure S6 ^{1}H *NMR spectrum of* ***4c*** *(400 MHz,* CD_2Cl_2*).*

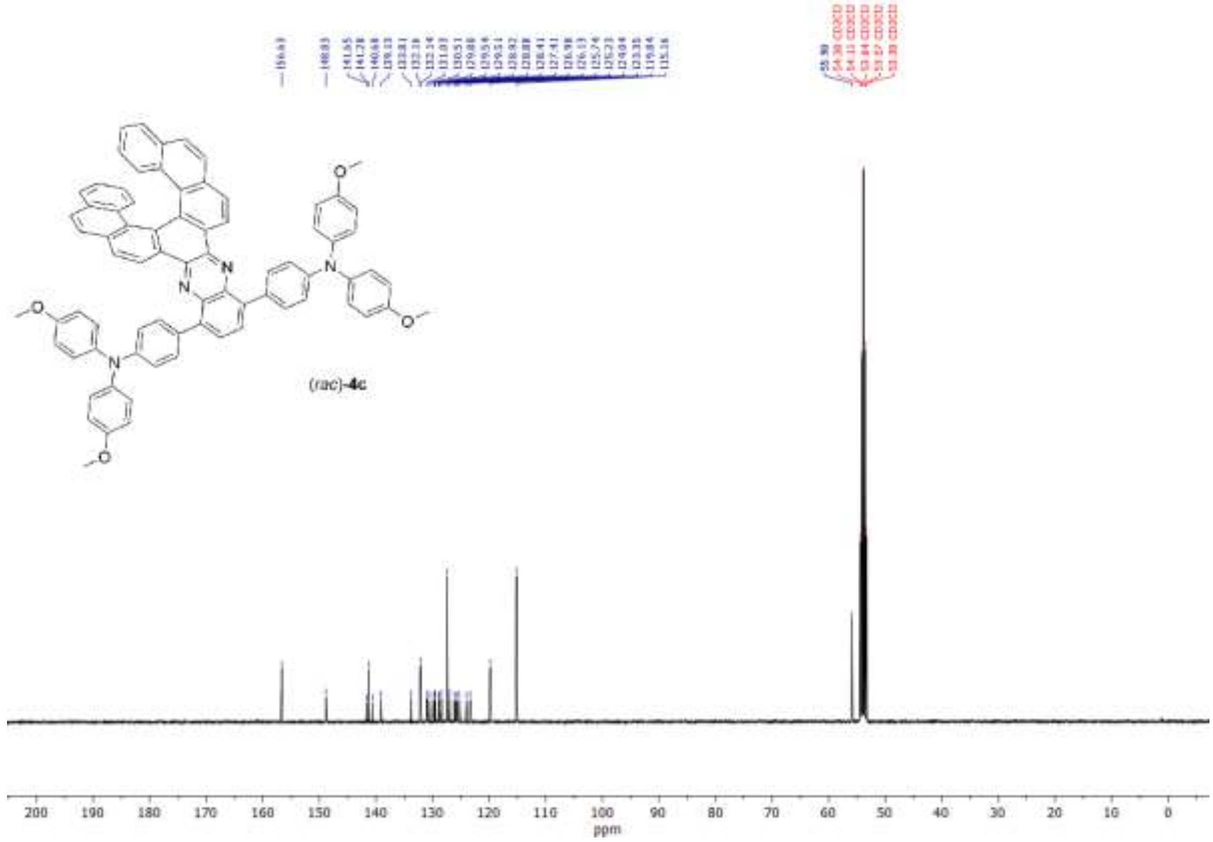

Figure S7 $^{13}C\{^{1}H\}$ *NMR spectrum of* ***4c*** *(101 MHz,* CD_2Cl_2*).*

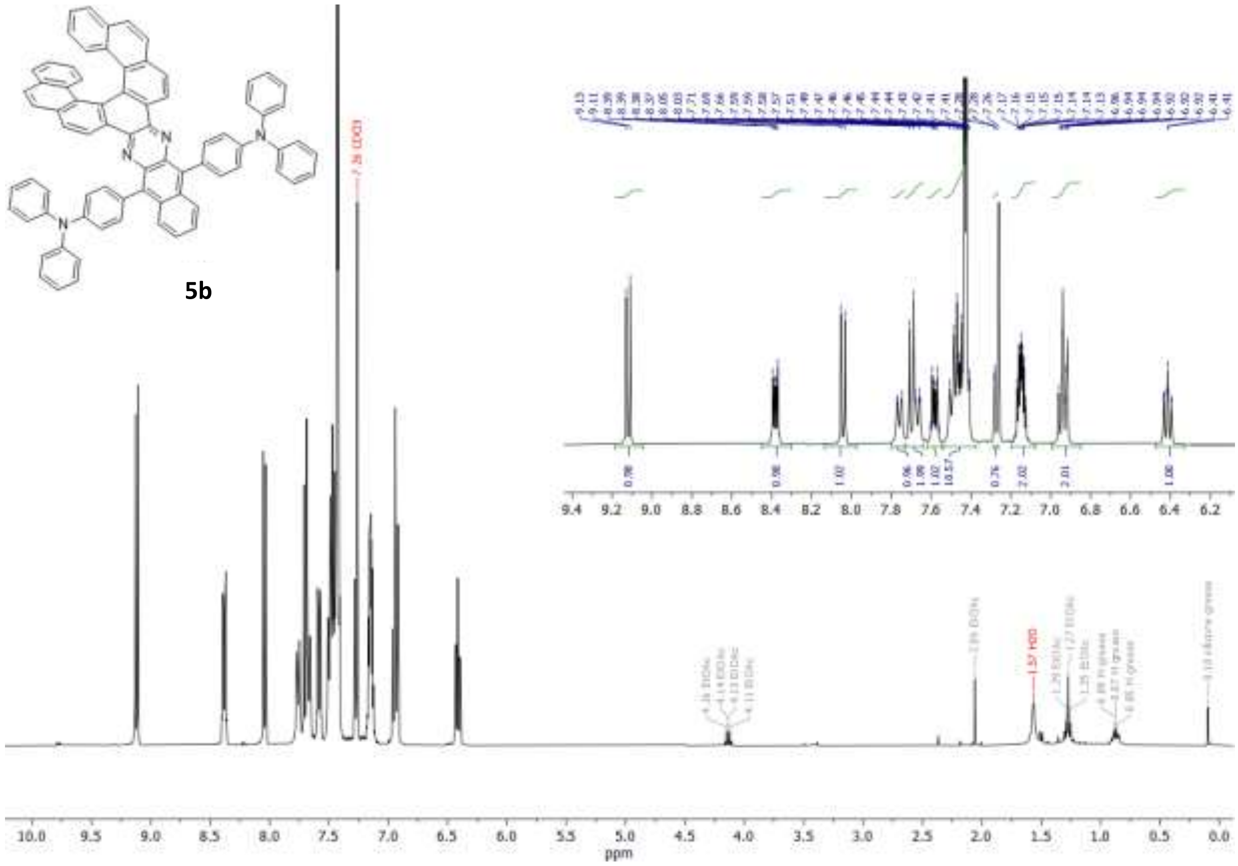

Figure S8 *^{1}H NMR spectrum of **5b** (400 MHz, $CDCl_3$).*

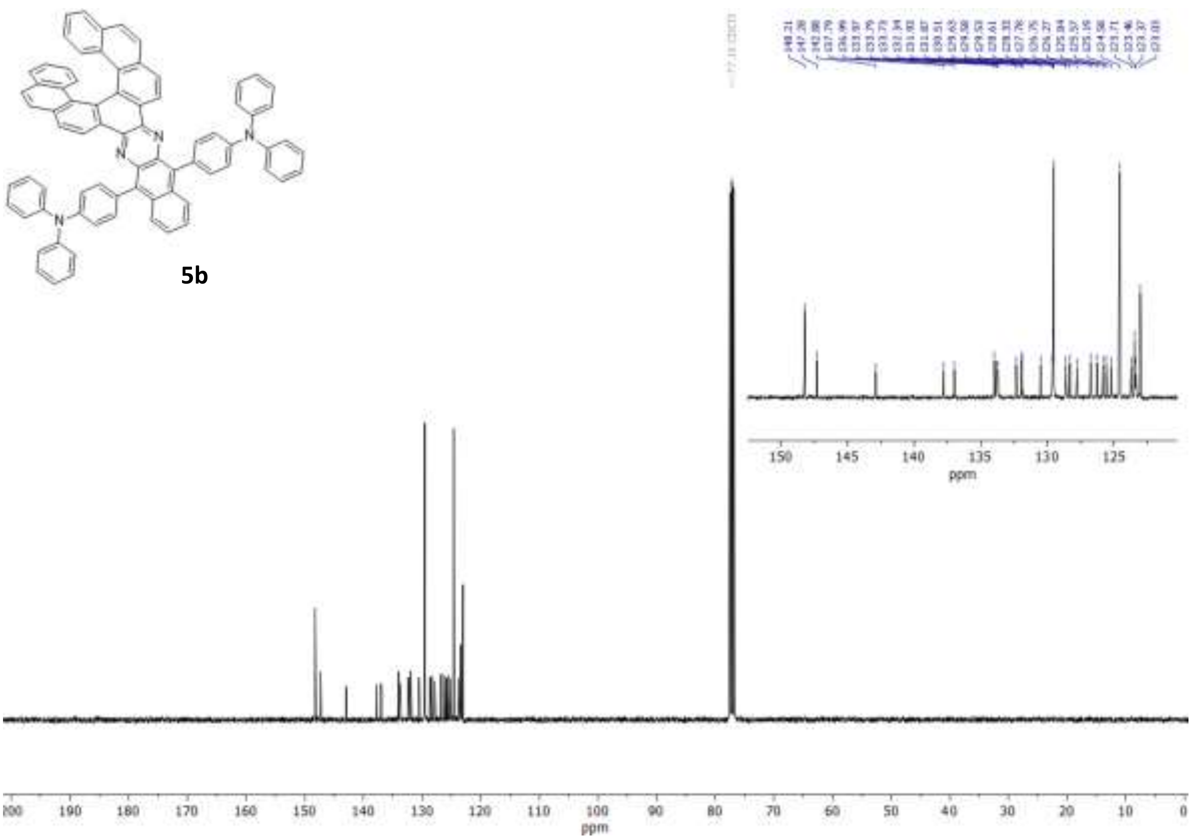

Figure S9 *^{13}C {^{1}H} NMR spectrum of **5b** (101 MHz, $CDCl_3$).*

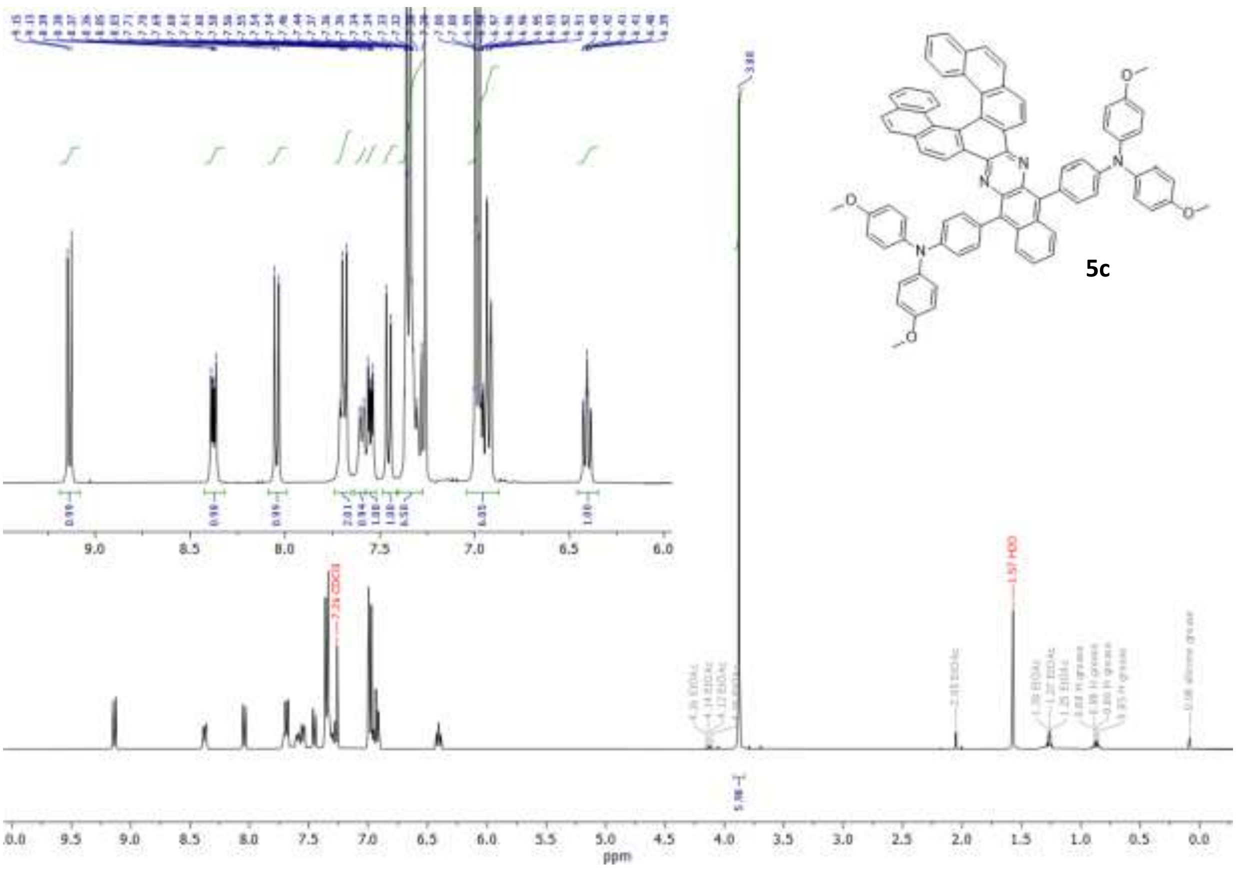

Figure S10 *^{1}H NMR spectrum of **5c** (400 MHz, $CDCl_3$)*

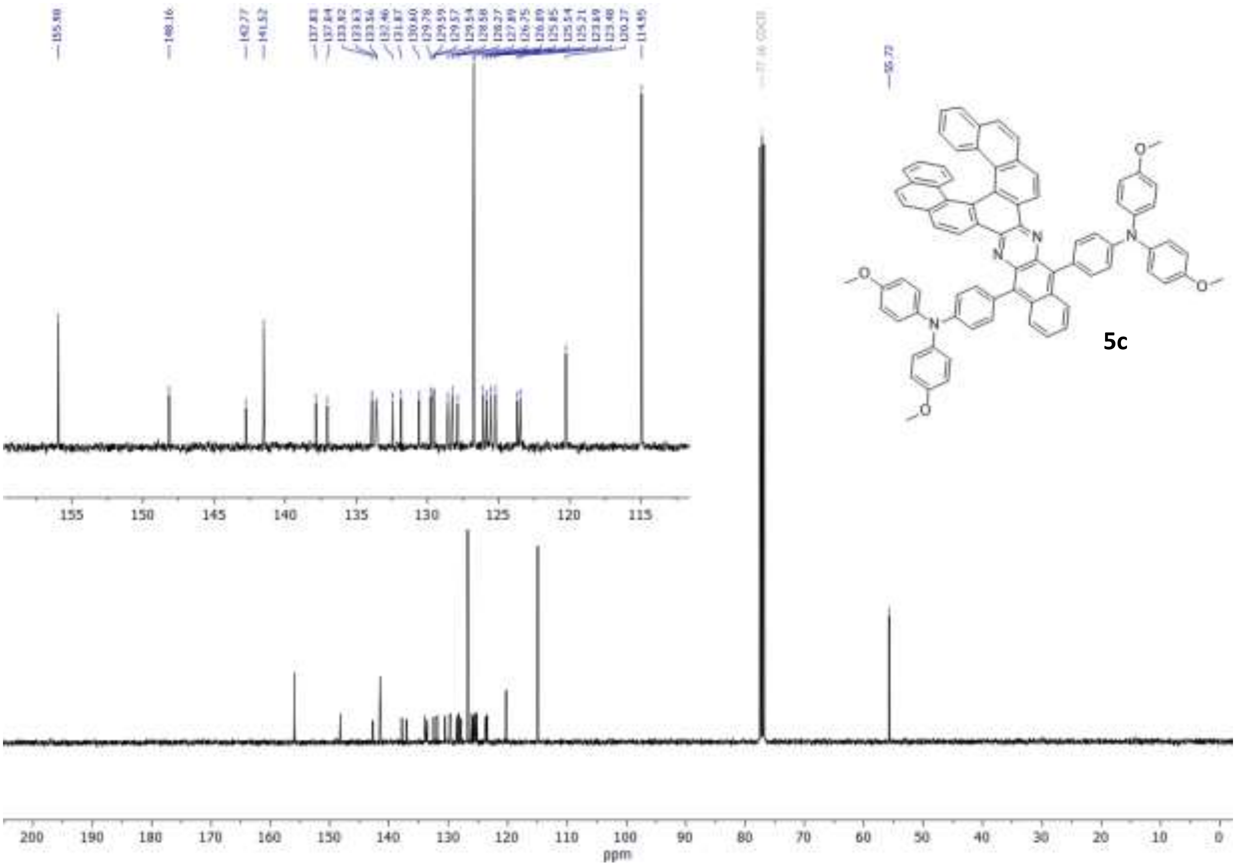

Figure S11 *^{13}C {^{1}H} NMR spectrum of **5c** (101 MHz, $CDCl_3$)*

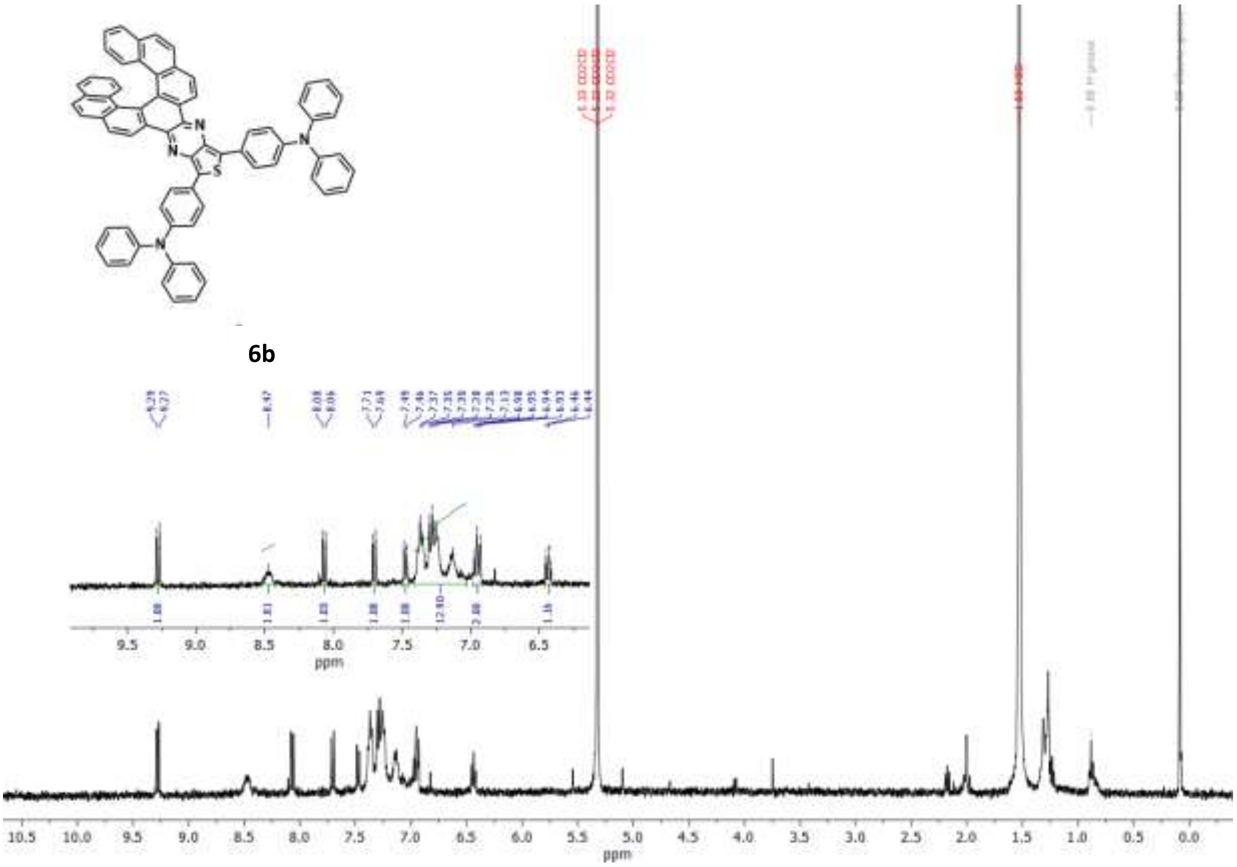

Figure S12 *^{1}H NMR spectrum of **6b** (400 MHz, $CDCl_3$).*

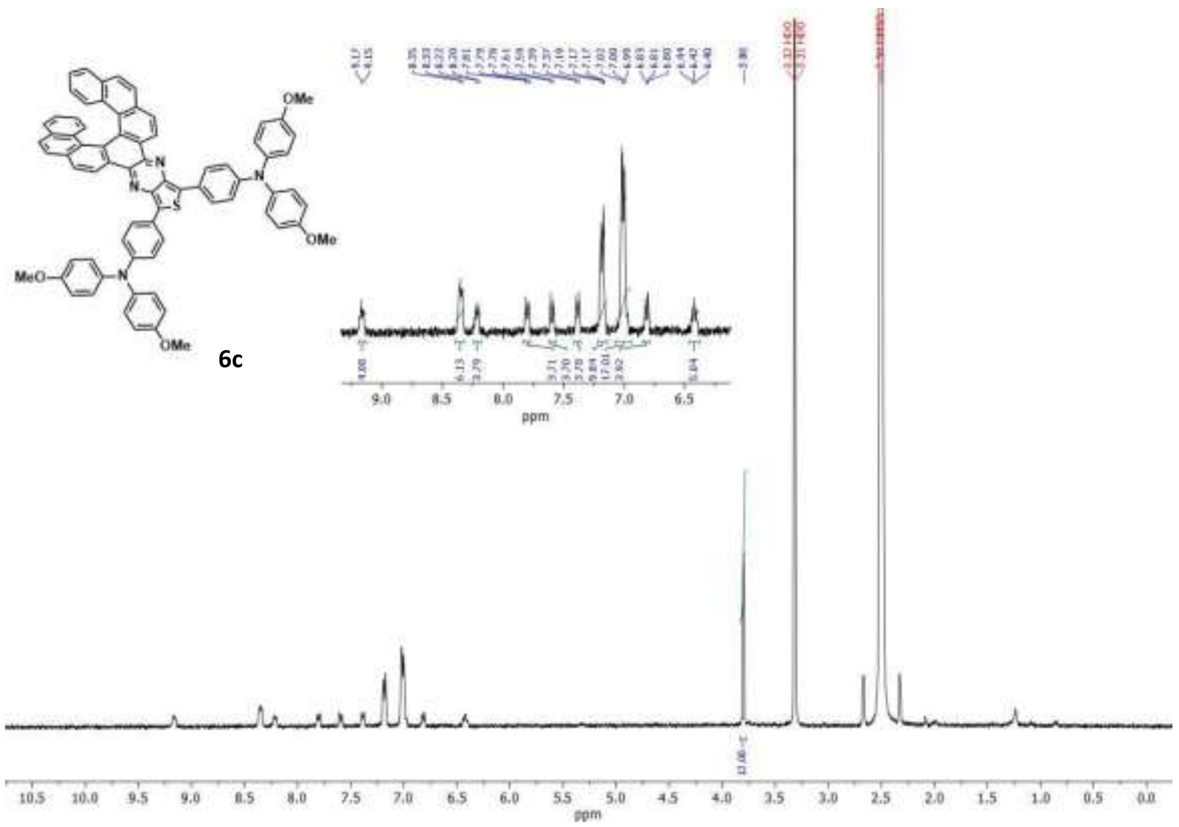

6c

Figure S13 ^{1}H NMR spectrum of **6c** (400 MHz, DMSO-d_6).

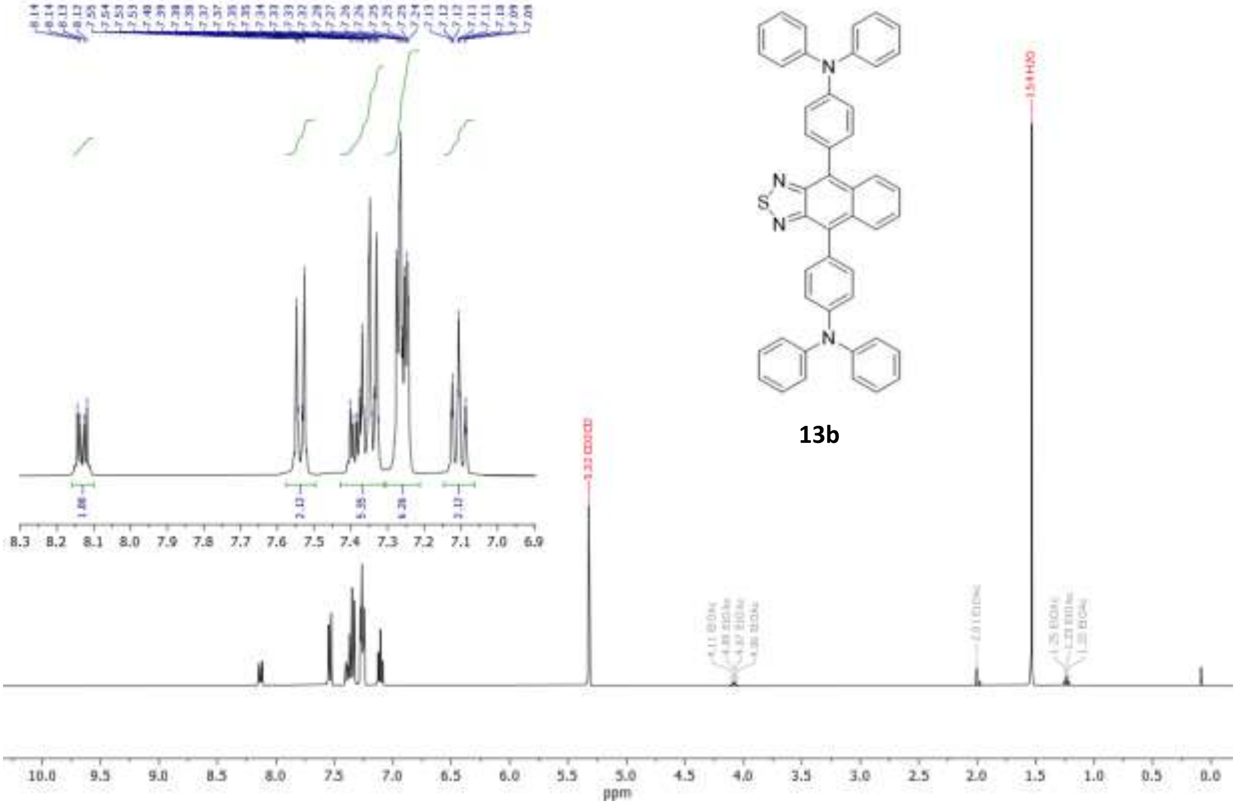

13b

Figure S14 ^{1}H NMR spectrum of **13b** (400 MHz, CD_2Cl_2)

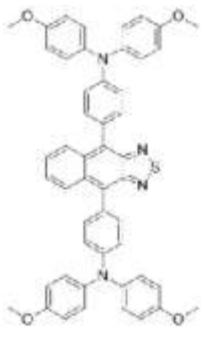

13c

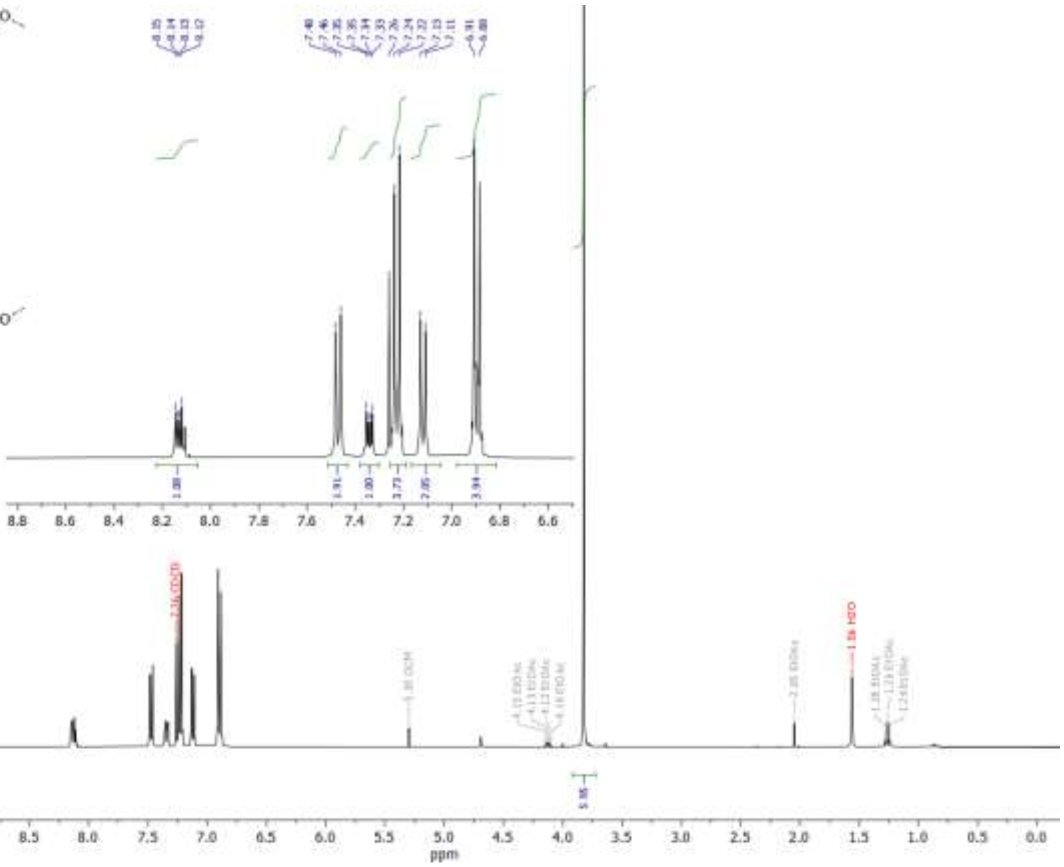

Figure S15 ^{1}H NMR spectrum of **13c** (400 MHz, $CDCl_3$)

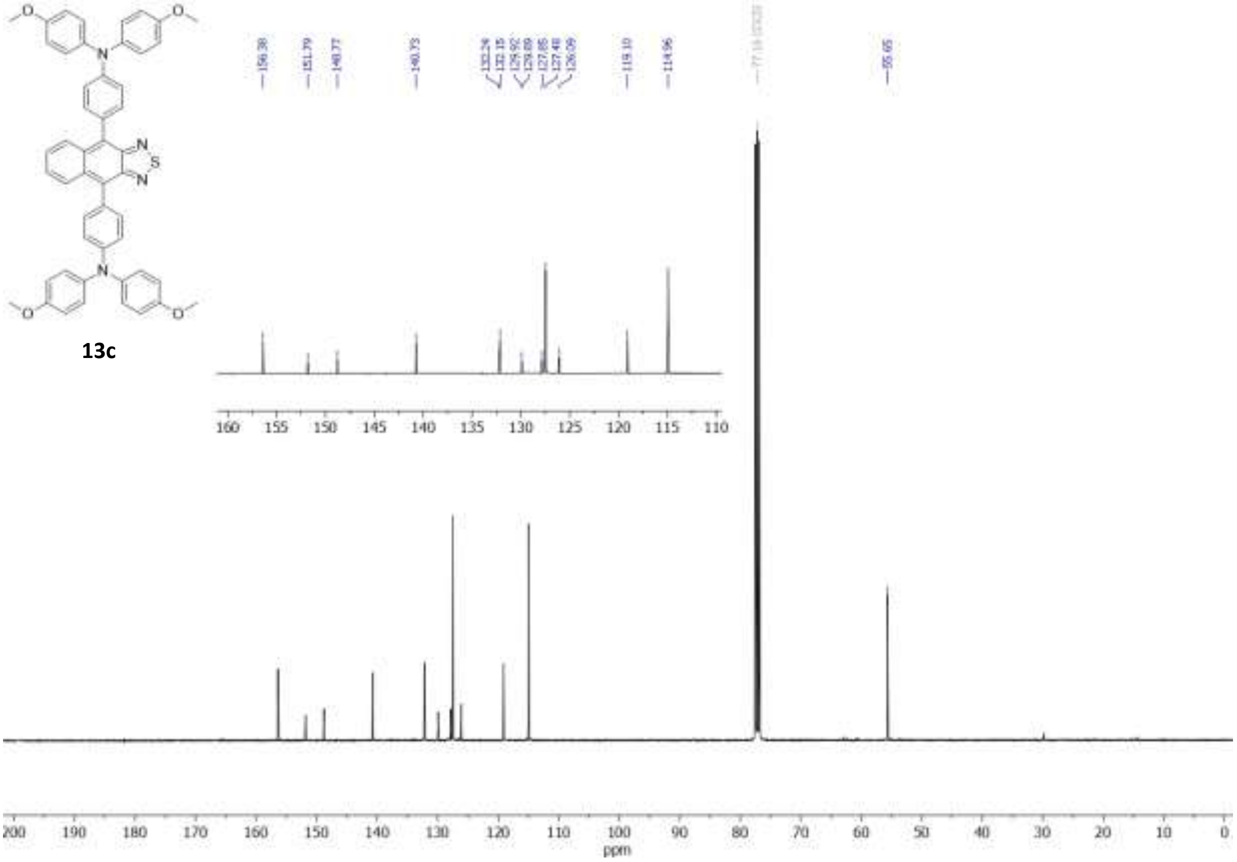

13c

Figure S16 *^{13}C {^{1}H} NMR spectrum of* ***13c*** *(101 MHz, $CDCl_3$)*

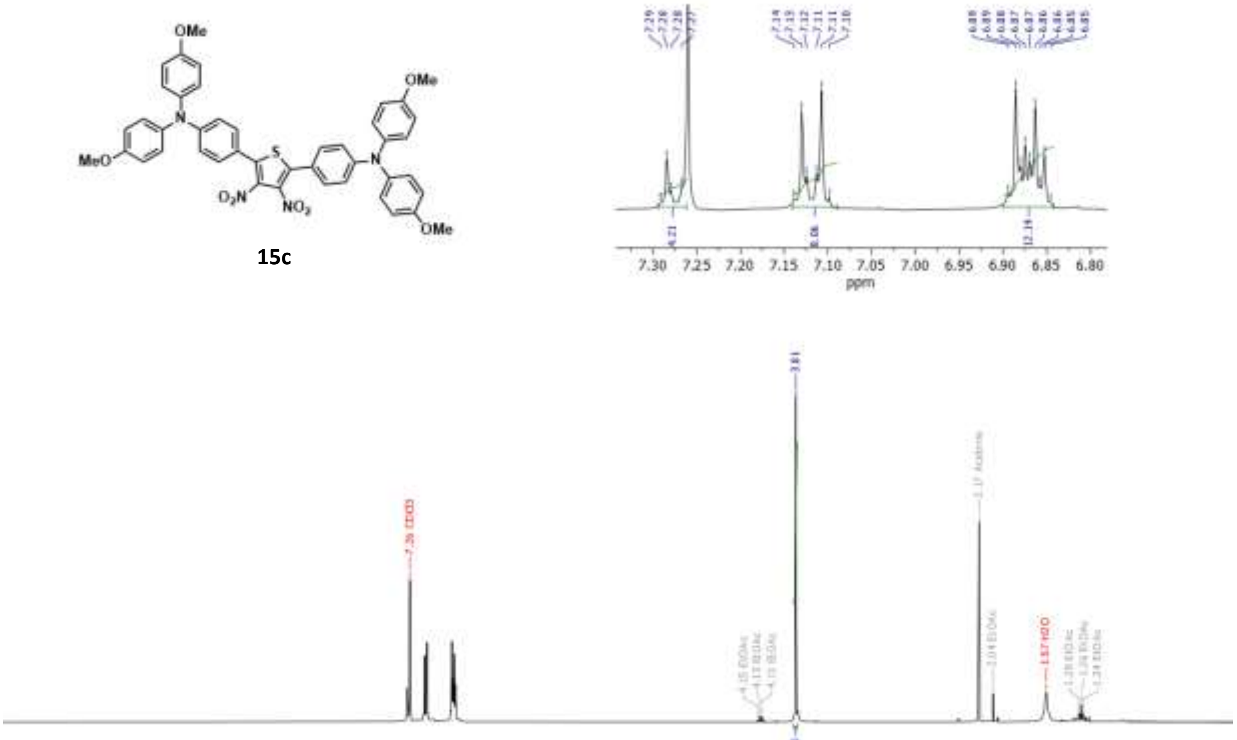

15c

Figure S17 *^{1}H NMR spectrum of* ***15c*** *(400 MHz, $CDCl_3$)*

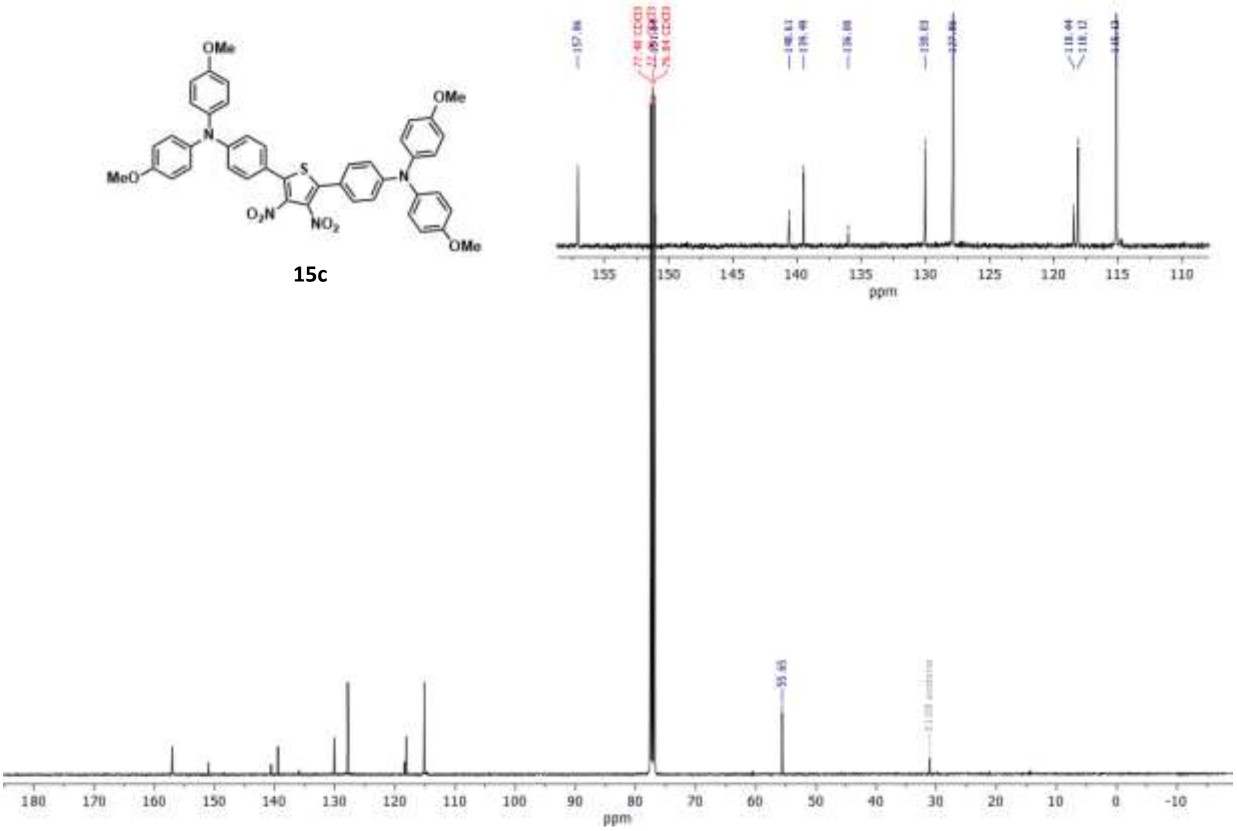

Figure S18 *^{13}C {^{1}H} NMR spectrum of* ***15c*** *(101 MHz, $CDCl_3$)*

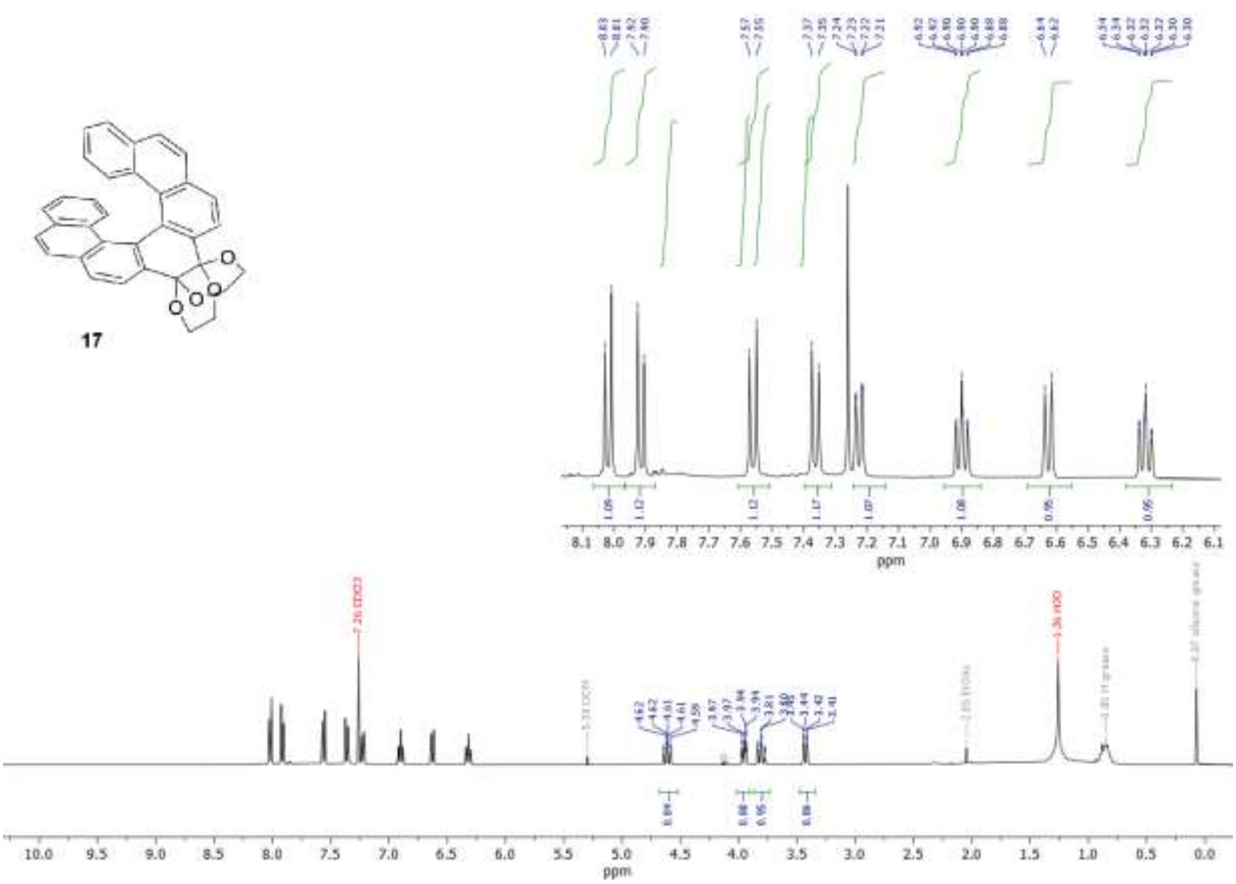

Figure S19 *^{1}H NMR spectrum of* ***17*** *(400 MHz, $CDCl_3$)*

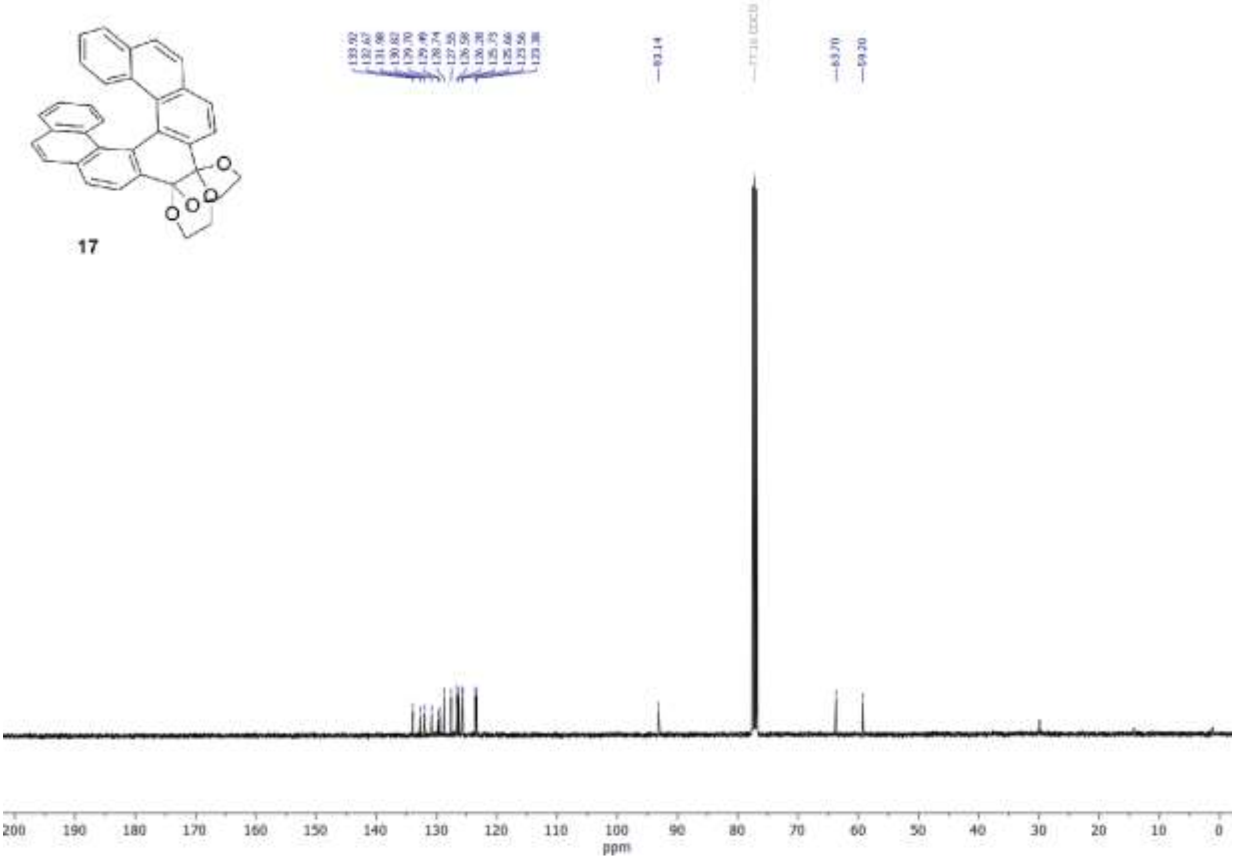

Figure S20 $^{13}C\{^1H\}$ *NMR spectrum of* ***17*** *(101 MHz, CDCl$_3$)*

4. DFT Calculation Details of **3**, **4–6**

Optimizations of structures and calculations of HOMO/LUMO energies were performed with the package Gaussian09[10]. B3LYP[11,12] was used as a functional in combination with 6-31G(d) basis set for optimization and energy calculations, with the exceptions of the adducts (*S*,*M*)-**3** and (*R*,*M*)-**3**, which were optimized at 6-311G(d,p)++ level. The pairs of enantiomers (*S*,*M*)/(*R*,*P*)-**3** and (*R*,*M*)/(*S*,*P*)-**3** are identical in energy, only one of the pair was calculated. The vibrational analysis showed that all structures correspond to the local minima in the potential energy surface. Cartesian coordinates all optimized structures are following.

4.1 Cartesian coordinates of optimized structures of **3**-**6**

4a

```
Total energy=                                        -1493.44599462
 Number of imaginary frequencies=                      0
Zero-point correction=                               0.451502 (Hartree/Particle)
 Thermal correction to Energy=                        0.476722
 Thermal correction to Enthalpy=                      0.477667
 Thermal correction to Gibbs Free Energy=             0.397757
 Sum of electronic and zero-point Energies=              -1492.994492
 Sum of electronic and thermal Energies=                 -1492.969272
 Sum of electronic and thermal Enthalpies=               -1492.968328
 Sum of electronic and thermal Free Energies=            -1493.048238

0 1
 C                  3.33214800    1.94688900   -1.22160300
 C                  3.35279200    3.21368600   -0.55565900
 C                  2.22915300    3.69842700    0.03726400
 C                  1.03771300    2.90819700    0.12744200
 C                  1.03401300    1.56072500   -0.33760300
 C                  2.15722600    1.13384300   -1.16670100
 C                 -0.14131600    3.47726500    0.67590700
 C                 -1.31195900    2.76696300    0.69343900
 C                 -1.31084300    1.39530800    0.34061600
 C                 -0.10678200    0.73976900   -0.00396600
 C                 -2.57686400    0.67526400    0.24905800
 C                 -2.57685000   -0.67527300   -0.24901700
 C                 -1.31081600   -1.39529100   -0.34059700
 C                 -0.10676900   -0.73973700    0.00400500
 C                 -1.31190300   -2.76693000   -0.69347700
 C                 -0.14123900   -3.47719900   -0.67599900
 C                  1.03778200   -2.90812500   -0.12752400
 C                  1.03405000   -1.56068100    0.33760100
 C                  2.22924800   -3.69832400   -0.03741600
 C                  3.35288100   -3.21358700    0.55552000
 C                  3.33220300   -1.94684100    1.22155800
 C                  2.15725300   -1.13382900    1.16673100
 N                 -3.70949400    1.31193800    0.52339000
 C                 -4.86979500    0.66118100    0.27863100
 C                 -4.86978200   -0.66122600   -0.27861400
```

N	-3.70947000	-1.31195500	-0.52335800
C	-6.10988000	1.29844900	0.55678000
C	-7.29213500	0.65268900	0.28188800
C	-7.29211400	-0.65279300	-0.28189400
C	-6.10985300	-1.29854600	-0.55678100
C	4.43896500	-1.53630300	2.00218800
C	2.11102200	-0.00671500	2.02414500
C	4.38323000	-0.39330900	2.77225700
C	3.19320000	0.35773300	2.80325000
C	4.43891600	1.53632100	-2.00220600
C	2.11103300	0.00663800	-2.02399700
C	4.38321700	0.39325300	-2.77217000
C	3.19321800	-0.35784200	-2.80307600
H	4.26139000	3.80933800	-0.59768000
H	2.21301700	4.69790200	0.46486000
H	-0.11373800	4.50951000	1.01597800
H	-2.25146400	3.21600300	0.99396700
H	-2.25139800	-3.21597900	-0.99401900
H	-0.11363800	-4.50942500	-1.01612500
H	2.21313600	-4.69777200	-0.46507800
H	4.26149800	-3.80921400	0.59748700
H	-8.23953500	1.14095500	0.49297000
H	-8.23950800	-1.14107700	-0.49298500
H	5.32903500	-2.16116700	2.00676800
H	1.19895300	0.57043000	2.09907200
H	5.23572200	-0.09396200	3.37582200
H	3.11574600	1.22528200	3.45281900
H	5.32896500	2.16121500	-2.00684900
H	1.19898700	-0.57055500	-2.09884900
H	5.23571500	0.09388100	-3.37571200
H	3.11579400	-1.22545900	-3.45255600
H	-6.08407500	2.29751100	0.98101400
H	-6.08403400	-2.29760500	-0.98101300

4b

```
Total energy=                                    -2990.45327484
 Number of imaginary frrequencies=                  0
Zero-point correction=                           0.969111 (Hartree/Particle)
 Thermal correction to Energy=                    1.025419
 Thermal correction to Enthalpy=                  1.026363
 Thermal correction to Gibbs Free Energy=         0.871085
 Sum of electronic and zero-point Energies=          -2989.484164
 Sum of electronic and thermal Energies=             -2989.427856
 Sum of electronic and thermal Enthalpies=           -2989.426912
 Sum of electronic and thermal Free Energies=        -2989.582190
```

0 1

C	-1.45054700	-2.81472800	-0.01809600
C	-0.72150900	-1.57527700	0.00667600
C	0.72155700	-1.57525900	-0.00667900
C	1.45062500	-2.81469400	0.01808800
C	2.93194800	-2.87138600	0.02811000
C	-2.93186800	-2.87144600	-0.02811800

C	3.60913800	-3.80520600	-0.77478600
C	4.99459700	-3.92558300	-0.74650800
C	5.76408700	-3.09822400	0.08672700
C	5.09836800	-2.15557400	0.88872400
C	3.71265600	-2.04987200	0.86118700
C	-3.60904600	-3.80528300	0.77476900
C	-4.99450300	-3.92567800	0.74648900
C	-5.76400400	-3.09831900	-0.08673600
C	-5.09829800	-2.15564900	-0.88872100
C	-3.71258800	-2.04993000	-0.86118300
N	-7.17807200	-3.20961400	-0.11757300
C	-7.87610600	-3.09622800	-1.35180400
C	-7.90304000	-3.44131200	1.08416700
N	7.17815600	-3.20950000	0.11756500
C	7.87618500	-3.09613000	1.35180000
C	7.90312800	-3.44118400	-1.08417500
C	-9.06787700	-2.35770600	-1.43050300
C	-9.75418200	-2.25729900	-2.63920000
C	-9.25887600	-2.87225500	-3.79103600
C	-8.06933700	-3.60011700	-3.71772400
C	-7.38572600	-3.72181200	-2.50945500
C	-8.96288600	-4.36199700	1.11042800
C	-9.67769300	-4.57795600	2.28711800
C	-9.34006500	-3.89734000	3.45883800
C	-8.28104600	-2.98711700	3.43769200
C	-7.57221100	-2.75081100	2.26139900
C	9.06795800	-2.35761300	1.43051200
C	9.75426000	-2.25722100	2.63921200
C	9.25894700	-2.87218800	3.79104000
C	8.06940700	-3.60004500	3.71771500
C	7.38580000	-3.72172500	2.50944300
C	8.96297200	-4.36187100	-1.11044600
C	9.67778100	-4.57781600	-2.28713700
C	9.34015900	-3.89718300	-3.45884800
C	8.28114300	-2.98695800	-3.43769300
C	7.57230400	-2.75066600	-2.26139900
C	-0.70508500	-3.98212100	-0.01503600
C	0.70519000	-3.98210500	0.01501600
N	-1.39652000	-0.40726500	0.07920400
C	-0.71284000	0.72935200	0.07253200
C	0.71282300	0.72937100	-0.07253100
N	1.39653500	-0.40722700	-0.07920600
C	-1.39906200	1.99028900	0.32756400
C	-0.65734500	3.19439500	0.34063400
C	0.65726300	3.19441400	-0.34062800
C	1.39901200	1.99032700	-0.32756100
C	1.23654200	4.33194200	-1.01712500
C	2.64764200	4.33187700	-1.22101700
C	3.40420800	3.15401000	-0.98647700
C	2.78051500	1.98526000	-0.63907200
C	0.47939900	5.45380900	-1.56457700
C	1.17898700	6.62315200	-1.99803300
C	2.61009400	6.64078500	-1.98847600
C	3.31050100	5.51877000	-1.67301000

C	-2.78056500	1.98518400	0.63907500
C	-3.40428900	3.15391700	0.98648300
C	-2.64775400	4.33180300	1.22102600
C	-1.23665400	4.33190600	1.01713400
C	-3.31064400	5.51867700	1.67302300
C	-2.61026700	6.64071000	1.98849200
C	-1.17916000	6.62311500	1.99804700
C	-0.47954100	5.45379200	1.56458700
C	-0.45825600	7.72711000	2.51219500
C	0.91646400	5.41031100	1.80382500
C	1.59580500	6.48939700	2.33761400
C	0.91122700	7.67385600	2.66851300
C	-0.91660400	5.41029100	-1.80381600
C	0.45805300	7.72712900	-2.51217800
C	-1.59597400	6.48936000	-2.33760300
C	-0.91142800	7.67383800	-2.66849700
H	3.03979000	-4.44502700	-1.44349500
H	5.48686400	-4.65902100	-1.37685800
H	5.67520600	-1.50678500	1.54003400
H	3.22671900	-1.31600900	1.49336000
H	-3.03968900	-4.44510300	1.44347200
H	-5.48676100	-4.65912900	1.37683200
H	-5.67514500	-1.50685900	-1.54002100
H	-3.22665900	-1.31605100	-1.49334500
H	-9.44971800	-1.86609100	-0.54136200
H	-10.67510400	-1.68146300	-2.68170900
H	-9.79271100	-2.78543400	-4.73309600
H	-7.67482200	-4.09078000	-4.60374800
H	-6.46901700	-4.30033000	-2.45458900
H	-9.22036200	-4.90335000	0.20563300
H	-10.49498200	-5.29455200	2.28897800
H	-9.89476900	-4.07364700	4.37601800
H	-8.01081400	-2.44393200	4.33957000
H	-6.75889700	-2.03216500	2.24693300
H	9.44980400	-1.86598900	0.54137700
H	10.67518300	-1.68138800	2.68173000
H	9.79277900	-2.78537800	4.73310200
H	7.67488700	-4.09071600	4.60373300
H	6.46908900	-4.30023900	2.45456800
H	9.22044400	-4.90323700	-0.20565800
H	10.49506900	-5.29441400	-2.28900500
H	9.89486500	-4.07347800	-4.37602900
H	8.01091400	-2.44375900	-4.33956400
H	6.75899200	-2.03201900	-2.24692500
H	-1.22251700	-4.93673400	-0.04566200
H	1.22264400	-4.93670600	0.04563400
H	4.47754700	3.18035700	-1.15736700
H	3.31746500	1.04573200	-0.57308300
H	3.12200500	7.54549300	-2.30722900
H	4.39481900	5.50016200	-1.75059200
H	-3.31748900	1.04564200	0.57308400
H	-4.47762900	3.18023400	1.15737400
H	-4.39496200	5.50003900	1.75060500
H	-3.12220300	7.54540300	2.30724800

H	-1.01332200	8.61283700	2.81248500
H	1.46547400	4.50229600	1.59346500
H	2.66555400	6.41398600	2.51234600
H	1.45239900	8.52412900	3.07452000
H	-1.46559100	4.50226100	-1.59345900
H	1.01309600	8.61287200	-2.81246500
H	-2.66572100	6.41392100	-2.51233600
H	-1.45262200	8.52409800	-3.07450200

4c

```
Total energy=                                     -3448.62223999
 Number of imaginary frequencies=                    0
Zero-point correction=                            1.097666 (Hartree/Particle)
 Thermal correction to Energy=                    1.164690
 Thermal correction to Enthalpy=                  1.165634
 Thermal correction to Gibbs Free Energy=         0.984286
 Sum of electronic and zero-point Energies=          -3447.524574
 Sum of electronic and thermal Energies=             -3447.457550
 Sum of electronic and thermal Enthalpies=           -3447.456606
 Sum of electronic and thermal Free Energies=        -3447.637954
```

0 1

C	1.45189700	-2.40817500	0.00497400
C	0.72190900	-1.16895100	-0.01342000
C	-0.72181600	-1.16896800	0.01225900
C	-1.45178200	-2.40820200	-0.00677300
C	-2.93238000	-2.46571500	-0.00311100
C	2.93250000	-2.46562200	0.00142700
C	-3.60257400	-3.40412600	0.80016700
C	-4.98727100	-3.52703800	0.78459900
C	-5.76888600	-2.69912800	-0.03971000
C	-5.10835600	-1.75075100	-0.84231000
C	-3.72345200	-1.64177600	-0.82391200
C	3.60285100	-3.40339500	-0.80245700
C	4.98755800	-3.52624500	-0.78675900
C	5.76897900	-2.69892300	0.03831200
C	5.10828900	-1.75119700	0.84153900
C	3.72338500	-1.64226400	0.82299100
N	7.17729700	-2.81392700	0.05882300
C	7.90505800	-2.58497600	1.26208200
C	7.88994000	-3.17614900	-1.12088200
N	-7.17718700	-2.81419100	-0.05997800
C	-7.90546500	-2.58363000	-1.26261200
C	-7.88944600	-3.17775700	1.11957700
C	9.06641900	-1.80519900	1.25826100
C	9.80216800	-1.59845300	2.42703300
C	9.36618000	-2.15701200	3.63325700
C	8.19713200	-2.93190600	3.64800200
C	7.48458500	-3.15234700	2.47823400
C	8.88359600	-4.15975800	-1.08376500
C	9.60578100	-4.50105100	-2.22931300
C	9.32230300	-3.87037700	-3.44549600
C	8.32029100	-2.88989800	-3.49372700

C	7.62307300	-2.54016500	-2.34641900
C	-9.06708000	-1.80424300	-1.25714800
C	-9.80338500	-1.59597200	-2.42530200
C	-9.36771500	-2.15256700	-3.63254100
C	-8.19842700	-2.92706800	-3.64892900
C	-7.48532400	-3.14905000	-2.47979500
C	-8.88259900	-4.16183800	1.08178600
C	-9.60443000	-4.50445600	2.22716100
C	-9.32107700	-3.87464500	3.44382300
C	-8.31955600	-2.89369400	3.49272100
C	-7.62269500	-2.54265800	2.34559100
C	0.70507200	-3.57496800	0.00820500
C	-0.70491800	-3.57497600	-0.01070200
N	1.39574500	-0.00076800	-0.09151400
C	0.71183800	1.13572500	-0.07835800
C	-0.71176100	1.13568100	0.07831000
N	-1.39565700	-0.00082600	0.09094000
C	1.39604300	2.39692600	-0.33857200
C	0.65498300	3.60137800	-0.34457100
C	-0.65493400	3.60122400	0.34558900
C	-1.39597300	2.39676100	0.33910100
C	-1.23013400	4.73830100	1.02675900
C	-2.63937700	4.73677000	1.24232800
C	-3.39686300	3.55836100	1.01367000
C	-2.77500500	2.39053100	0.66116200
C	-0.46997500	5.86078700	1.56873400
C	-1.16725500	7.02865300	2.00955400
C	-2.59834300	7.04486100	2.01195900
C	-3.29968700	5.92263800	1.70063900
C	2.77508400	2.39085300	-0.66059800
C	3.39692800	3.55883800	-1.01261500
C	2.63942100	4.73732300	-1.24081700
C	1.23017400	4.73874200	-1.02526900
C	3.29971400	5.92338600	-1.69864900
C	2.59835200	7.04571900	-2.00953200
C	1.16726400	7.02948700	-2.00714400
C	0.46999900	5.86143400	-1.56679300
C	0.44316900	8.13385000	-2.51597800
C	-0.92807100	5.81956900	-1.79395300
C	-1.61040400	6.89880900	-2.32267800
C	-0.92733900	8.08198000	-2.66046000
C	0.92809500	5.81884700	1.79587900
C	-0.44317300	8.13282400	2.51882400
C	1.61041700	6.89788600	2.32502900
C	0.92733700	8.08091500	2.66328100
H	-3.02722100	-4.04595400	1.46097900
H	-5.47014500	-4.26409500	1.41656100
H	-5.68935500	-1.09952600	-1.48609200
H	-3.24486100	-0.90309200	-1.45538900
H	3.02762000	-4.04476600	-1.46382200
H	5.47058700	-4.26280200	-1.41918800
H	5.68917900	-1.10046900	1.48592600
H	3.24464400	-0.90408800	1.45495000
H	9.40436700	-1.35978900	0.32833900

H	10.69891200	-0.99195100	2.38336000
H	7.87469700	-3.36609000	4.58865500
H	6.58954600	-3.76518200	2.49885000
H	9.10090000	-4.66022200	-0.14587800
H	10.37136500	-5.26467600	-2.16072900
H	8.11621700	-2.40317400	-4.44174300
H	6.85914500	-1.77101200	-2.39184700
H	-9.40479500	-1.36034300	-0.32642200
H	-10.70031700	-0.98984000	-2.38033200
H	-7.87625800	-3.35975200	-4.59036500
H	-6.59011400	-3.76158800	-2.50171000
H	-9.09979200	-4.66161800	0.14350600
H	-10.36963100	-5.26841400	2.15806600
H	-8.11557700	-2.40766500	4.44111400
H	-6.85912900	-1.77317400	2.39152700
H	1.22271500	-4.52896700	0.03451400
H	-1.22253200	-4.52897400	-0.03756000
H	-4.46824000	3.58390700	1.19280600
H	-3.30993900	1.45008100	0.59882200
H	-3.10835500	7.94821700	2.33544700
H	-4.38270000	5.90265200	1.78701100
H	3.31002800	1.45038500	-0.59862700
H	4.46830900	3.58448100	-1.19171200
H	4.38272800	5.90345500	-1.78501400
H	3.10835100	7.94921300	-2.33265700
H	0.99629900	9.01829900	-2.82129500
H	-1.47600800	4.91320900	-1.57677700
H	-2.68108500	6.82471700	-2.48810000
H	-1.47076200	8.93210200	-3.06207700
H	1.47603900	4.91257700	1.57834700
H	-0.99631300	9.01714300	2.82449600
H	2.68109900	6.82374300	2.49042000
H	1.47075000	8.93088500	3.06523200
O	9.95746600	-4.13126400	-4.62797000
O	10.00017400	-2.01257200	4.83621400
O	-10.00222200	-2.00649600	-4.83503200
O	-9.95591200	-4.13685200	4.62617300
C	10.97317200	-5.12033100	-4.63575200
H	10.58359600	-6.10510800	-4.34654300
H	11.34060100	-5.16823300	-5.66192200
H	11.80353000	-4.85545200	-3.96809400
C	11.18340500	-1.23286200	4.87761700
H	10.99617200	-0.19337100	4.57814800
H	11.52102700	-1.25076600	5.91494500
H	11.96946800	-1.65237000	4.23607000
C	-11.18584200	-1.22729100	-4.87472400
H	-10.99897500	-0.18815600	-4.57378900
H	-11.52392300	-1.24379300	-5.91192600
H	-11.97140300	-1.64813600	-4.23343900
C	-10.97107500	-5.12649000	4.63331700
H	-10.58101400	-6.11080000	4.34317600
H	-11.33825400	-5.17547600	5.65952500
H	-11.80172200	-4.86149300	3.96606700

5a

```
Total energy=                                       -1647.08325556
 Number of imaginary frequencies=                     0
Zero-point correction=                               0.498071 (Hartree/Particle)
 Thermal correction to Energy=                        0.526037
 Thermal correction to Enthalpy=                      0.526981
 Thermal correction to Gibbs Free Energy=             0.440886
 Sum of electronic and zero-point Energies=              -1646.585185
 Sum of electronic and thermal Energies=                 -1646.557218
 Sum of electronic and thermal Enthalpies=               -1646.556274
 Sum of electronic and thermal Free Energies=            -1646.642370


0 1
 C                  -3.99559800   -0.66803700   -0.27353800
 C                  -3.99560700    0.66802500    0.27353300
 C                  -5.21425000    1.30352900    0.54310800
 C                  -6.43297600    0.66810700    0.28108900
 C                  -6.43297900   -0.66812700   -0.28109100
 C                  -5.21424400   -1.30355400   -0.54311200
 N                  -2.82347200   -1.32255400   -0.50863800
 C                  -1.69978300   -0.68395000   -0.24175200
 C                  -1.69979300    0.68393300    0.24176600
 N                  -2.82348100    1.32254700    0.50865000
 C                  -0.42998200   -1.40436900   -0.31330100
 C                   0.77084400   -0.74069600    0.02286300
 C                   0.77083500    0.74070100   -0.02284300
 C                  -0.43000100    1.40436300    0.31330900
 C                  -0.42931900   -2.78414100   -0.62941800
 C                   0.74134500   -3.49432800   -0.58416500
 C                   1.91602300   -2.91078600   -0.04352600
 C                   1.90927600   -1.55036000    0.38473600
 C                   3.10788300   -3.69698400    0.07359900
 C                   4.22863700   -3.19474900    0.65706600
 C                   4.20497400   -1.90929700    1.28676300
 C                   3.02994500   -1.09896900    1.20501200
 C                   1.90925000    1.55037500   -0.38473700
 C                   1.91597400    2.91081400    0.04348700
 C                   0.74129200    3.49434900    0.58412100
 C                  -0.42936000    2.78414200    0.62939700
 C                   3.02992700    1.09897700   -1.20499800
 C                   4.20493700    1.90932700   -1.28678400
 C                   4.22857700    3.19480200   -0.65713200
 C                   3.10781800    3.69703200   -0.07367000
 C                  -7.68993100    1.29814800    0.54613700
 C                  -7.68994000   -1.29817000   -0.54613100
 C                   5.30870800   -1.47628700    2.05920900
 C                   2.98028200    0.05237700    2.02895800
 C                   2.98028900   -0.05241000   -2.02888900
```

C	5.30867800	1.47630900	-2.05921600
C	4.05986900	-0.43989300	-2.80074100
C	4.05985500	0.43985100	2.80082600
C	5.24993600	0.31142700	-2.79577000
C	5.24994100	-0.31143900	2.79581500
C	-8.86978700	-0.65764900	-0.27683200
C	-8.86978100	0.65762300	0.27684000
H	-5.18967400	2.30828400	0.95559700
H	-5.18966500	-2.30829400	-0.95558600
H	-1.36713800	-3.24081100	-0.92377100
H	0.77102400	-4.53518200	-0.89664900
H	3.09442200	-4.70789300	-0.32627100
H	5.13748300	-3.78821700	0.71943100
H	0.77095400	4.53521100	0.89657900
H	-1.36718600	3.24080400	0.92374100
H	5.13741000	3.78828600	-0.71952200
H	3.09434000	4.70795400	0.32616800
H	-7.68728100	2.30053400	0.96705500
H	-7.68729300	-2.30055900	-0.96704500
H	6.19895300	-2.10038500	2.08500600
H	2.06771800	0.63098800	2.08381200
H	2.06774000	-0.63104900	-2.08370700
H	6.19890900	2.10042500	-2.08504200
H	3.98013500	-1.32624700	-3.42408900
H	3.98010000	1.32617300	3.42421700
H	6.10023300	-0.00547000	-3.39342900
H	6.10023200	0.00545200	3.39348600
H	-9.81668000	-1.14882200	-0.48354000
H	-9.81667200	1.14879000	0.48355400

5b

Total energy= -3144.15810723
Number of imaginary frequencies= 0
Zero-point correction= 1.014049 (Hartree/Particle)
Thermal correction to Energy= 1.073325
Thermal correction to Enthalpy= 1.074269
Thermal correction to Gibbs Free Energy= 0.911634
Sum of electronic and zero-point Energies= -3143.144058
Sum of electronic and thermal Energies= -3143.084782
Sum of electronic and thermal Enthalpies= -3143.083838
Sum of electronic and thermal Free Energies= -3143.246474

0 1

C	1.44609800	-2.77938200	0.05242100
C	0.72235600	-1.56440000	0.01356900
C	-0.72230800	-1.56442400	-0.01360100
C	-1.44601300	-2.77942900	-0.05244400
C	-2.93470500	-2.74705500	-0.07322200
C	2.93478900	-2.74697500	0.07320200
C	-3.68737500	-3.32457300	0.96169800
C	-5.07886300	-3.29459900	0.95243500
C	-5.77011400	-2.66769600	-0.09599800
C	-5.02559800	-2.07805100	-1.13126100

C	-3.63606400	-2.12263100	-1.11716900
C	3.68747200	-3.32451400	-0.96169700
C	5.07896000	-3.29452000	-0.95242700
C	5.77019600	-2.66757800	0.09599200
C	5.02566600	-2.07793000	1.13124400
C	3.63613300	-2.12253100	1.11714500
N	7.18927900	-2.62789300	0.10973300
C	7.89479500	-2.82184600	1.32918400
C	7.90766700	-2.39364900	-1.09492100
N	-7.18919800	-2.62802000	-0.10972100
C	-7.89473600	-2.82201000	-1.32915300
C	-7.90756100	-2.39374500	1.09494100
C	9.00436800	-2.02187600	1.64660000
C	9.69735500	-2.22235900	2.83857400
C	9.28934700	-3.20606000	3.74154000
C	8.18129400	-3.99731500	3.43251500
C	7.49312600	-3.81718500	2.23458800
C	9.07099000	-3.12475700	-1.38505400
C	9.77756100	-2.88345800	-2.56140500
C	9.33081400	-1.92777700	-3.47598100
C	8.16911200	-1.20611300	-3.19466600
C	7.46588200	-1.42655900	-2.01234700
C	-9.00432400	-2.02205800	-1.64656500
C	-9.69733600	-2.22257700	-2.83851700
C	-9.28934200	-3.20629900	-3.74146700
C	-8.18127800	-3.99753900	-3.43244500
C	-7.49308300	-3.81737200	-2.23453800
C	-9.07087800	-3.12484500	1.38511800
C	-9.77742100	-2.88351800	2.56148000
C	-9.33064800	-1.92782000	3.47602600
C	-8.16894900	-1.20616800	3.19467000
C	-7.46574900	-1.42664100	2.01233800
C	0.72492300	-3.99694300	0.04439000
C	-0.72479700	-3.99696900	-0.04442200
N	1.40379900	-0.38582900	-0.03102900
C	0.72100400	0.74162000	-0.04112000
C	-0.72103200	0.74159600	0.04109500
N	-1.40379000	-0.38587600	0.03100100
C	1.41584000	2.00787700	-0.25871500
C	0.67387900	3.20931700	-0.30922000
C	-0.67398800	3.20929400	0.30919300
C	-1.41591100	2.00783100	0.25868800
C	-1.28142300	4.34621000	0.95898100
C	-2.70118800	4.34934100	1.09487500
C	-3.44808300	3.17451400	0.82100000
C	-2.81036700	2.00503300	0.50162400
C	-0.54877600	5.46718400	1.54108900
C	-1.26584900	6.63789000	1.93997600
C	-2.69501500	6.65855100	1.86192000
C	-3.38198000	5.53828600	1.51380200
C	2.81029800	2.00512500	-0.50164600
C	3.44797700	3.17462900	-0.82101500
C	2.70104500	4.34943200	-1.09488900
C	1.28127900	4.34625500	-0.95900300

C	3.38180100	5.53840100	-1.51380400
C	2.69480200	6.65864600	-1.86191900
C	1.26563800	6.63793900	-1.93998600
C	0.54860000	5.46720600	-1.54111200
C	0.56768700	7.74079400	-2.48677000
C	-0.83415100	5.42073200	-1.84642100
C	-1.48962600	6.49904900	-2.41027300
C	-0.79265100	7.68488500	-2.70723900
C	0.83398000	5.42075300	1.84638300
C	-0.56793000	7.74076400	2.48676100
C	1.48942400	6.49908900	2.41023500
C	0.79241300	7.68489900	2.70721700
H	-3.17385600	-3.80700400	1.78800000
H	-5.63607400	-3.75358300	1.76195300
H	-5.54319300	-1.58456900	-1.94677400
H	-3.08174000	-1.65467200	-1.92377300
H	3.17396300	-3.80697800	-1.78798700
H	5.63618000	-3.75352200	-1.76192900
H	5.54325000	-1.58443000	1.94675300
H	3.08179800	-1.65456800	1.92374000
H	9.31745600	-1.24728000	0.95472700
H	10.55330800	-1.59396300	3.06780400
H	7.85590900	-4.77180000	4.12124800
H	6.64143100	-4.44423300	1.99334500
H	9.41513400	-3.87804600	-0.68443800
H	10.67544800	-3.45847200	-2.76938800
H	7.81205100	-0.45461300	-3.89310400
H	6.57119100	-0.85364200	-1.79324000
H	-9.31740300	-1.24744600	-0.95470500
H	-10.55329900	-1.59419300	-3.06774500
H	-7.85590400	-4.77204200	-4.12116400
H	-6.64138000	-4.44441000	-1.99329700
H	-9.41504200	-3.87814800	0.68452600
H	-10.67530500	-3.45852500	2.76949700
H	-7.81186700	-0.45465700	3.89308400
H	-6.57105900	-0.85373300	1.79319800
H	-4.52810300	3.20395500	0.93616600
H	-3.34286500	1.06628000	0.40336200
H	-3.21926100	7.56425000	2.15458800
H	-4.46818500	5.52254800	1.53860700
H	3.34282600	1.06638900	-0.40338200
H	4.52799700	3.20410500	-0.93617500
H	4.46800600	5.52269800	-1.53860200
H	3.21902100	7.56436400	-2.15457900
H	1.13399600	8.62754400	-2.75941200
H	-1.39043200	4.51198400	-1.66229000
H	-2.54921600	6.42205300	-2.63500100
H	-1.31558100	8.53403600	-3.13705200
H	1.39029000	4.51202600	1.66223700
H	-1.13426500	8.62749400	2.75941400
H	2.54901900	6.42212700	2.63495100
H	1.31531900	8.53406400	3.13703100
C	1.39301900	-5.26188600	0.13877200
C	-1.39283900	-5.26194100	-0.13883000

C	-0.70582000	-6.44432800	-0.08265200
H	-1.24010200	-7.38668200	-0.16006900
C	0.70604600	-6.44430000	0.08261200
H	1.24036300	-7.38663100	0.16005300
H	2.46895100	-5.26777100	0.26224200
H	-2.46876900	-5.26787300	-0.26231100
H	-9.82737400	-3.35419000	-4.67266000
H	-9.87974600	-1.74833600	4.39513400
H	9.82735900	-3.35392300	4.67274900
H	9.87993500	-1.74831400	-4.39507900

5c

Total energy= -3602.25392772
Number of imaginary frequencies= 0
Zero-point correction= 1.143906 (Hartree/Particle)
Thermal correction to Energy= 1.213849
Thermal correction to Enthalpy= 1.214794
Thermal correction to Gibbs Free Energy= 1.026648
Sum of electronic and zero-point Energies= -3601.110022
Sum of electronic and thermal Energies= -3601.040078
Sum of electronic and thermal Enthalpies= -3601.039134
Sum of electronic and thermal Free Energies= -3601.227280

0 1

C	-1.44762800	-2.50426100	-0.04376600
C	-0.72264500	-1.28955600	-0.00778500
C	0.72268600	-1.28954500	0.00756500
C	1.44768500	-2.50424100	0.04338700
C	2.93584000	-2.47221500	0.05730000
C	-2.93578000	-2.47225300	-0.05766600
C	3.68665000	-3.05542400	-0.97588600
C	5.07777300	-3.02511500	-0.97315100
C	5.77687000	-2.39388600	0.07003000
C	5.03214000	-1.79897900	1.10464500
C	3.64303000	-1.84190300	1.09388500
C	-3.68658500	-3.05565800	0.97541500
C	-5.07770800	-3.02535000	0.97270400
C	-5.77682400	-2.39392300	-0.07034800
C	-5.03209800	-1.79882300	-1.10485900
C	-3.64298900	-1.84174700	-1.09412000
N	-7.19053800	-2.35581900	-0.08058600
C	-7.90337700	-2.42764200	-1.31207900
C	-7.91933000	-2.24827300	1.13858400
N	7.19059300	-2.35579700	0.08028800
C	7.90341900	-2.42814500	1.31174900
C	7.91934600	-2.24771900	-1.13885500
C	-8.96400400	-1.55559900	-1.57928100
C	-9.68139400	-1.63499500	-2.77492500
C	-9.32628400	-2.58445600	-3.73909300
C	-8.25819800	-3.45672000	-3.48316200
C	-7.56565100	-3.38708700	-2.28318000
C	-9.03890100	-3.05202700	1.37829100
C	-9.77332200	-2.93488500	2.56042700

C	-9.37725600	-2.01712900	3.53909200
C	-8.25003600	-1.21424800	3.31109200
C	-7.53952800	-1.31979100	2.12431800
C	8.96422400	-1.55640200	1.57923200
C	9.68160600	-1.63632600	2.77484500
C	9.32631900	-2.58602500	3.73872000
C	8.25802900	-3.45796200	3.48252100
C	7.56549200	-3.38781100	2.28256300
C	9.03892100	-3.05135400	-1.37895900
C	9.77328200	-2.93369800	-2.56108000
C	9.37715400	-2.01553300	-3.53933700
C	8.24992700	-1.21277500	-3.31094700
C	7.53947700	-1.31883400	-2.12418400
C	-0.72502500	-3.72136700	-0.04116100
C	0.72510700	-3.72135800	0.04061000
N	-1.40321200	-0.11093300	0.04626500
C	-0.72010800	1.01631900	0.05031900
C	0.72011600	1.01633700	-0.05021800
N	1.40323700	-0.11090500	-0.04631800
C	-1.41156900	2.28247400	0.28008600
C	-0.66912100	3.48400700	0.31979300
C	0.66909500	3.48406500	-0.31932800
C	1.41155800	2.28253600	-0.27980000
C	1.26647800	4.62046500	-0.97956000
C	2.68376300	4.62272400	-1.13916700
C	3.43470200	3.44804800	-0.87593800
C	2.80199300	2.27914300	-0.54470500
C	0.52497400	5.74132700	-1.55071600
C	1.23589300	6.91092700	-1.96379700
C	2.66617800	6.93076600	-1.91017100
C	3.35800200	5.81061400	-1.57153700
C	-2.80200300	2.27902400	0.54499400
C	-3.43472600	3.44787100	0.87640600
C	-2.68380100	4.62251700	1.13981200
C	-1.26651700	4.62029900	0.98019900
C	-3.35805000	5.81033400	1.57236700
C	-2.66623700	6.93044000	1.91117300
C	-1.23595100	6.91060800	1.96479200
C	-0.52502300	5.74108100	1.55152500
C	-0.52950100	8.01296400	2.50164200
C	0.86290600	5.69541200	1.83273800
C	1.52715200	6.77316500	2.38729300
C	0.83445400	7.95781300	2.69867700
C	-0.86295100	5.69568800	-1.83194700
C	0.52943400	8.01336000	-2.50047800
C	-1.52720600	6.77352100	-2.38633700
C	-0.83451900	7.95822500	-2.69752900
H	3.17113400	-3.54261700	-1.79831800
H	5.62919100	-3.48964200	-1.78316900
H	5.54995200	-1.30079100	1.91684700
H	3.09266600	-1.36869600	1.90030200
H	-3.17106400	-3.54299900	1.79775700
H	-5.62910500	-3.49003200	1.78264600
H	-5.54990900	-1.30047600	-1.91696200

H	-3.09263900	-1.36838700	-1.90045700
H	-9.23710500	-0.80908200	-0.84072500
H	-10.49964900	-0.94536400	-2.94472000
H	-7.99885100	-4.19368200	-4.23609800
H	-6.74937300	-4.07511900	-2.08999100
H	-9.34516900	-3.77396300	0.62844000
H	-10.63733300	-3.57176800	2.70834800
H	-7.95832400	-0.50109700	4.07505400
H	-6.67672600	-0.68462000	1.95355600
H	9.23747300	-0.80970200	0.84091600
H	10.49997000	-0.94688100	2.94486900
H	7.99850900	-4.19509300	4.23523300
H	6.74906000	-4.07560800	2.08918400
H	9.34523500	-3.77360300	-0.62943000
H	10.63729300	-3.57051000	-2.70931400
H	7.95816300	-0.49930900	-4.07459500
H	6.67667000	-0.68375300	-1.95311800
H	4.51268700	3.47691100	-1.00912700
H	3.33551600	1.34018200	-0.45376300
H	3.18599100	7.83554400	-2.21348500
H	4.44365100	5.79399100	-1.61472900
H	-3.33551500	1.34007000	0.45391400
H	-4.51271100	3.47670100	1.00960400
H	-4.44369900	5.79369200	1.61556000
H	-3.18605900	7.83516500	2.21462900
H	-1.09180900	8.89873800	2.78562700
H	1.41658100	4.78755200	1.63702300
H	2.59056900	6.69657800	2.59340300
H	1.36400600	8.80662600	3.12106500
H	-1.41662000	4.78779100	-1.63638100
H	1.09173400	8.89918400	-2.78432000
H	-2.59062100	6.69695500	-2.59246600
H	-1.36407900	8.80710000	-3.11978600
C	-1.39326900	-4.98633900	-0.13401800
C	1.39338300	-4.98632700	0.13327900
C	0.70624700	-6.16884600	0.07989900
H	1.24094400	-7.11114900	0.15599000
C	-0.70610300	-6.16885000	-0.08083600
H	-1.24077400	-7.11115300	-0.15710100
H	-2.46962200	-4.99162000	-0.25379400
H	2.46974100	-4.99159800	0.25301200
O	10.01293600	-1.82266700	-4.73470600
O	9.94954900	-2.74714400	4.94519100
O	-9.94951000	-2.74502500	-4.94563900
O	-10.01309500	-1.82478500	4.73451300
C	11.15611100	-2.61184200	-5.01729100
H	10.91520800	-3.68268000	-5.04105900
H	11.50169900	-2.30105200	-6.00438100
H	11.95732900	-2.44311700	-4.28580600
C	11.03135000	-1.88600800	5.25801600
H	10.71910300	-0.83382500	5.28347500
H	11.37549700	-2.18109300	6.25040500
H	11.85765300	-1.99530800	4.54333900
C	-11.15626300	-2.61410800	5.01671700

```
 H                -10.91533500   -3.68495000    5.04003500
 H                -11.50189300   -2.30374800    6.00392800
 H                -11.95745700   -2.44508600    4.28527600
 C                -11.03122400   -1.88364100   -5.25810100
 H                -10.71884300   -0.83148900   -5.28320400
 H                -11.37542900   -2.17831000   -6.25059300
 H                -11.85750300   -1.99309700   -4.54342200
```

6a

```
Total energy=                                        -1814.18587770
 Number of imaginary frequencies=                      0
Zero-point correction=                              0.417590 (Hartree/Particle)
 Thermal correction to Energy=                       0.442530
 Thermal correction to Enthalpy=                     0.443474
 Thermal correction to Gibbs Free Energy=            0.363942
 Sum of electronic and zero-point Energies=             -1813.768288
 Sum of electronic and thermal Energies=                -1813.743348
 Sum of electronic and thermal Enthalpies=              -1813.742404
 Sum of electronic and thermal Free Energies=           -1813.821935

0 1
 C                  3.38326700   -1.94751400   -1.22268500
 C                  3.40943600   -3.21030400   -0.54866700
 C                  2.28996300   -3.69387000    0.05271000
 C                  1.09698600   -2.90590200    0.14359300
 C                  1.08747800   -1.56131400   -0.33222800
 C                  2.20677000   -1.13696100   -1.16871100
 C                 -0.07571600   -3.47033400    0.70736600
 C                 -1.24682200   -2.75943500    0.73067400
 C                 -1.25025500   -1.39175700    0.36589400
 C                 -0.05098200   -0.74110800    0.00329000
 C                 -2.52469000   -0.67818800    0.27148900
 C                 -2.52472300    0.67814900   -0.27171200
 C                 -1.25033800    1.39172000   -0.36617000
 C                 -0.05105100    0.74113700   -0.00345400
 C                 -1.24689100    2.75939900   -0.73094000
 C                 -0.07576400    3.47034300   -0.70753000
 C                  1.09680400    2.90590200   -0.14368700
 C                  1.08738400    1.56139300    0.33213700
 C                  2.28986000    3.69389200   -0.05278900
 C                  3.40919200    3.21036400    0.54882600
 C                  3.38299800    1.94761200    1.22291800
 C                  2.20653300    1.13702800    1.16883400
 N                 -3.63940800   -1.31878600    0.56874200
 C                 -4.80041600   -0.66112800    0.30149600
 C                 -4.80045500    0.66105600   -0.30154200
 N                 -3.63947300    1.31875200   -0.56883900
 C                 -6.08088200   -1.14169200    0.52567300
 S                 -7.25207900   -0.00006900    0.00006900
 C                 -6.08094900    1.14158800   -0.52561200
 C                  2.15459600   -0.01544100   -2.03270600
 C                  3.23340900    0.34710000   -2.81774500
 C                  4.42495700   -0.40111500   -2.78672400
```

C	4.48603600	-1.53947400	-2.00989500
C	4.48573900	1.53951200	2.01015400
C	4.42458500	0.40113100	2.78692500
C	3.23298900	-0.34704700	2.81790300
C	2.15424300	0.01549600	2.03280100
H	4.31922000	-3.80411000	-0.59106400
H	2.27857900	-4.69024900	0.48760000
H	-0.04486700	-4.49939600	1.05664700
H	-2.18287500	-3.20695200	1.04393600
H	-2.18291500	3.20694800	-1.04424400
H	-0.04498100	4.49941300	-1.05677300
H	2.27864900	4.69017600	-0.48789500
H	4.31901000	3.80412000	0.59136700
H	-6.37083900	-2.09016900	0.95420000
H	-6.37096500	2.09005900	-0.95411400
H	1.24084300	0.55907400	-2.10817900
H	3.15168800	1.21083300	-3.47184400
H	5.27460400	-0.10363500	-3.39519100
H	5.37745600	-2.16240500	-2.01409600
H	5.37720000	2.16238800	2.01432100
H	5.27420600	0.10349600	3.39535100
H	3.15123300	-1.21070300	3.47210100
H	1.24045400	-0.55898000	2.10814900

6b

Total energy= -3311.27617238
Number of imaginary frerquencies= 0
Zero-point correction= 0.934189 (Hartree/Particle)
Thermal correction to Energy= 0.990438
Thermal correction to Enthalpy= 0.991383
Thermal correction to Gibbs Free Energy= 0.835769
Sum of electronic and zero-point Energies= -3310.341983
Sum of electronic and thermal Energies= -3310.285734
Sum of electronic and thermal Enthalpies= -3310.284790
Sum of electronic and thermal Free Energies= -3310.440403

0 1

C	1.55206100	7.28641700	-1.75129600
C	2.98260800	7.23286000	-1.74496100
C	3.62682700	6.06824700	-1.46835600
C	2.90656500	4.90151300	-1.05356300
C	1.49671100	4.96384500	-0.84717000
C	0.79550000	6.13967500	-1.35557700
C	3.60293000	3.68191800	-0.85502900
C	2.92187900	2.53680400	-0.53697700
C	1.54362000	2.59954900	-0.21997400
C	0.86426700	3.83798300	-0.20312300
C	0.79715000	1.36158500	-0.00416900
C	-0.64910900	1.43476100	0.11249600
C	-1.25978700	2.72826800	0.41373500
C	-0.45187600	3.88549000	0.47741800
C	-2.63766700	2.79172400	0.73201500
C	-3.19351100	3.97876800	1.12983800

C	-2.37137600	5.10086100	1.40691400
C	-0.96241000	5.02621300	1.19885200
C	-2.96419400	6.30683400	1.90337100
C	-2.19998100	7.37441300	2.25575800
C	-0.77194200	7.27416000	2.25969400
C	-0.14108500	6.08297100	1.78337800
N	1.43471200	0.20453900	-0.03727700
C	0.67702900	-0.92087200	-0.01929900
C	-0.77340400	-0.84381000	-0.02735700
N	-1.40662500	0.35285200	0.06858800
C	1.17168100	-2.23322000	-0.05062600
S	-0.17757400	-3.33152200	-0.10513900
C	-1.40427700	-2.09553500	-0.08359900
C	-0.60076400	6.17303600	-1.59326400
C	-1.22592000	7.30160700	-2.08931900
C	-0.48374500	8.46075300	-2.38191700
C	0.88657400	8.44113200	-2.22682700
C	0.01161200	8.31537100	2.81134900
C	1.37557800	8.17742700	2.96266900
C	1.99044200	6.96848900	2.58794400
C	1.24986800	5.95032900	2.01746900
H	3.53797200	8.12084000	-2.03445800
H	4.70824300	5.99892400	-1.54860700
H	4.67525500	3.65873100	-1.02860500
H	3.41155500	1.57092000	-0.50462200
H	-3.22724000	1.88779600	0.63738200
H	-4.26251900	4.05818100	1.30711300
H	-4.04703100	6.34780800	1.98386600
H	-2.65815500	8.29465100	2.60786500
H	-1.19373500	5.28727000	-1.41180900
H	-2.29749300	7.28528900	-2.26430100
H	-0.98208500	9.34934200	-2.75782900
H	1.48417700	9.30746200	-2.49829600
H	-0.49090900	9.21980900	3.14422900
H	1.96466900	8.97943600	3.39739600
H	3.05344200	6.82538500	2.75738900
H	1.74540800	5.02103100	1.77295300
C	2.54330800	-2.72408600	-0.05628500
C	2.86520700	-4.00586400	-0.54894800
C	3.60474500	-1.94125700	0.44618000
C	4.16751300	-4.48323000	-0.54313800
H	2.08586100	-4.62934500	-0.97807600
C	4.90809100	-2.41743100	0.45918400
H	3.39043900	-0.95729800	0.84320400
C	5.21698800	-3.69672000	-0.03576600
H	4.38262800	-5.46652200	-0.94673000
H	5.69963500	-1.79919600	0.86874800
C	-2.82071300	-2.43942700	-0.11072600
C	-3.27581300	-3.71983600	0.26702900
C	-3.79418900	-1.50853700	-0.53252800
C	-4.61941800	-4.06121100	0.21530100
H	-2.56486200	-4.46514700	0.61205900
C	-5.14172400	-1.83933300	-0.56404300
H	-3.48025900	-0.51425800	-0.82286100

C	-5.58184500	-3.12313800	-0.19729700
H	-4.93312300	-5.05835100	0.50392600
H	-5.86656000	-1.09863200	-0.88413000
N	6.54728100	-4.17941700	-0.02555900
N	-6.95571200	-3.46321400	-0.24152800
C	6.81042200	-5.55033900	0.25099500
C	7.75378300	-6.26040300	-0.50846700
C	6.13499700	-6.21121700	1.28951900
C	8.01800700	-7.59913000	-0.22685600
H	8.27530400	-5.75715900	-1.31564000
C	6.39299100	-7.55514700	1.55124100
H	5.41105400	-5.66646900	1.88622300
C	7.33765300	-8.25691100	0.79969700
H	8.75088800	-8.13356200	-0.82453300
H	5.86150600	-8.05103600	2.35844300
H	7.54110200	-9.30199000	1.01154200
C	7.63100500	-3.29884000	-0.30087300
C	8.80217100	-3.35128100	0.47124200
C	7.54675700	-2.37006100	-1.35030100
C	9.86676700	-2.49732600	0.19078100
H	8.87046800	-4.06308000	1.28700200
C	8.60983000	-1.50809200	-1.61126000
H	6.64716200	-2.32985800	-1.95519600
C	9.77714400	-1.56791900	-0.84737200
H	10.76587900	-2.55094600	0.79796200
H	8.52874500	-0.79587300	-2.42744100
H	10.60607800	-0.89953700	-1.05866000
C	-7.52004700	-4.29797400	0.76353000
C	-8.41378400	-5.32321400	0.41566800
C	-7.19669300	-4.10533400	2.11618900
C	-8.97584500	-6.12993100	1.40286200
H	-8.66337000	-5.47990800	-0.62833000
C	-7.75002800	-4.92915600	3.09421200
H	-6.51310600	-3.30968200	2.39266300
C	-8.64543400	-5.94254600	2.74644500
H	-9.66565200	-6.91862600	1.11635100
H	-7.48957000	-4.76626500	4.13612400
H	-9.07994300	-6.57750900	3.51229000
C	-7.78229600	-2.97110900	-1.29022800
C	-9.07126000	-2.48934300	-1.01235200
C	-7.32364400	-2.96495500	-2.61719800
C	-9.88290600	-2.02044600	-2.04321500
H	-9.42913400	-2.48669600	0.01170200
C	-8.13616200	-2.47722700	-3.63853700
H	-6.33119700	-3.34224200	-2.83953400
C	-9.42089400	-2.00625100	-3.36073000
H	-10.87776900	-1.65134600	-1.81084100
H	-7.76576500	-2.47974100	-4.65967400
H	-10.05363900	-1.63364800	-4.16028600

6c

Total energy= -3769.37260195
Number of imaginary frequencies= 0

```
Zero-point correction=                           1.064179 (Hartree/Particle)
 Thermal correction to Energy=                    1.131058
 Thermal correction to Enthalpy=                  1.132002
 Thermal correction to Gibbs Free Energy=         0.951342
 Sum of electronic and zero-point Energies=          -3768.308423
 Sum of electronic and thermal Energies=             -3768.241544
 Sum of electronic and thermal Enthalpies=           -3768.240600
 Sum of electronic and thermal Free Energies=        -3768.421260

0 1
 C                 -1.43919300    7.90340600    1.51575600
 C                 -2.87029000    7.87129800    1.53175600
 C                 -3.53525700    6.70917900    1.29694900
 C                 -2.83844100    5.52066400    0.90441800
 C                 -1.43112200    5.55586700    0.67580900
 C                 -0.70552200    6.73482800    1.14114500
 C                 -3.55560700    4.30666600    0.75062000
 C                 -2.89610600    3.14291400    0.45579100
 C                 -1.52179000    3.17550000    0.11785200
 C                 -0.82445500    4.40246200    0.05555800
 C                 -0.79761500    1.92021000   -0.07377100
 C                  0.64687600    1.96724200   -0.21283100
 C                  1.27279600    3.24204000   -0.55993200
 C                  0.48249100    4.41006500   -0.64395000
 C                  2.64657500    3.27451500   -0.90000200
 C                  3.21532100    4.44097100   -1.33843200
 C                  2.40721800    5.56853300   -1.63401200
 C                  1.00059100    5.52233500   -1.40363700
 C                  3.01149100    6.75106500   -2.17144000
 C                  2.25933900    7.82111700   -2.54185800
 C                  0.83000900    7.74349900   -2.52315700
 C                  0.18769900    6.57571700   -2.00583800
 N                 -1.45256200    0.77470500    0.00060500
 C                 -0.71359100   -0.36301400    0.00292900
 C                  0.73890800   -0.30838800   -0.01027300
 N                  1.38836000    0.87511400   -0.14885800
 C                 -1.22886900   -1.66591700    0.07560000
 S                  0.10591000   -2.78309300    0.14331700
 C                  1.35229200   -1.56729900    0.07355600
 C                  0.69449200    6.75388800    1.35773500
 C                  1.34348300    7.88608100    1.81325300
 C                  0.62284600    9.06391100    2.08426000
 C                 -0.74973300    9.06062700    1.94935300
 C                  0.05473700    8.78153200   -3.09226400
 C                 -1.31307300    8.66069700   -3.22190100
 C                 -1.94094700    7.47185600   -2.80660300
 C                 -1.20813800    6.45829800   -2.21821300
 H                 -3.40822700    8.77517800    1.80455600
 H                 -4.61627300    6.65813400    1.39482800
 H                 -4.62548200    4.30453400    0.94031400
 H                 -3.40002900    2.18383800    0.45800700
 H                  3.22280600    2.36385100   -0.78879200
 H                  4.28271900    4.49800000   -1.53345600
 H                  4.09361300    6.77232400   -2.26827400
```

H	2.72669600	8.72412700	-2.92518100
H	1.27171200	5.85476500	1.19210000
H	2.41721300	7.85832300	1.97301400
H	1.13964800	9.95499200	2.42808900
H	-1.33055400	9.94303100	2.20518000
H	0.56633200	9.66880500	-3.45618800
H	-1.89581600	9.45980200	-3.67041200
H	-3.00815300	7.34047500	-2.95830900
H	-1.71411100	5.54362200	-1.94194800
C	-2.60646700	-2.13590900	0.11157200
C	-2.93951300	-3.40774100	0.62291300
C	-3.66783300	-1.34378400	-0.37715200
C	-4.24772800	-3.86640800	0.64798900
H	-2.16162200	-4.03997400	1.04206000
C	-4.97810500	-1.79874800	-0.35753700
H	-3.44785800	-0.36727900	-0.78928500
C	-5.29916900	-3.06949200	0.15627800
H	-4.46665100	-4.84236100	1.06659000
H	-5.76681500	-1.17103900	-0.75759300
C	2.76245400	-1.93259700	0.09879100
C	3.19531500	-3.23216700	-0.23752100
C	3.75660300	-1.00589900	0.48030200
C	4.53271400	-3.59515400	-0.18528200
H	2.46992800	-3.97767700	-0.55107200
C	5.09809200	-1.35769800	0.51508800
H	3.46282600	0.00322500	0.73915600
C	5.51778100	-2.66156700	0.18944200
H	4.82361900	-4.60757100	-0.44179900
H	5.83527900	-0.61687200	0.80421300
N	-6.63146500	-3.53021600	0.17971600
N	6.88013500	-3.02126200	0.23775000
C	-6.92035400	-4.91740800	0.02430300
C	-7.81806900	-5.56138900	0.88195500
C	-6.32246500	-5.66571400	-1.00523200
C	-8.12632200	-6.91380900	0.72127800
H	-8.28693800	-4.99712100	1.68137900
C	-6.60665200	-7.01496600	-1.15740100
H	-5.62890000	-5.17964200	-1.68336200
C	-7.51495700	-7.65143600	-0.29842100
H	-8.82952300	-7.37781900	1.40247600
H	-6.14707700	-7.59706400	-1.94933300
C	-7.71082000	-2.61782100	0.36444000
C	-8.84767500	-2.68214900	-0.44764900
C	-7.66557200	-1.64506000	1.37881800
C	-9.92181800	-1.81059000	-0.25659100
H	-8.89653600	-3.42721200	-1.23490900
C	-8.71936400	-0.76136400	1.56045000
H	-6.79375400	-1.58575200	2.02188400
C	-9.86015900	-0.83846000	0.74774200
H	-10.78740600	-1.89260700	-0.90310600
H	-8.68886100	-0.00849000	2.34115500
C	7.40519400	-4.00654100	-0.64874500
C	8.22593200	-5.03233700	-0.16912500
C	7.12830700	-3.95821000	-2.02654400

C	8.76971900	-5.98544300	-1.03255000
H	8.44857200	-5.08273900	0.89164600
C	7.64687400	-4.91456300	-2.88757800
H	6.49908100	-3.16472600	-2.41613200
C	8.47675000	-5.93493300	-2.39971700
H	9.40452800	-6.76332300	-0.62539900
H	7.43531500	-4.88218900	-3.95125600
C	7.75944900	-2.40877300	1.17835800
C	9.00576800	-1.91873200	0.77569000
C	7.40358200	-2.30339800	2.53452600
C	9.88745700	-1.34547600	1.69431000
H	9.29356900	-1.99232400	-0.26794200
C	8.26480700	-1.71522900	3.44931200
H	6.44270300	-2.68457000	2.86414900
C	9.51749100	-1.23494000	3.03912400
H	10.84639100	-0.98071800	1.34628800
H	7.99475800	-1.63041200	4.49673000
O	10.29424900	-0.68578500	4.02059000
O	-10.84556100	0.06978000	1.01779100
O	-7.73334900	-8.97861400	-0.54180500
O	8.94528600	-6.81808800	-3.33192700
C	9.78656000	-7.87071300	-2.89038400
H	9.27465600	-8.52659100	-2.17423500
H	10.04417100	-8.44550200	-3.78116200
H	10.70681300	-7.48867200	-2.42949800
C	11.56810700	-0.17815000	3.65950000
H	11.48836100	0.63913000	2.93094200
H	12.01226600	0.20487900	4.57942200
H	12.21588100	-0.96232300	3.24633300
C	-8.63957000	-9.67208700	0.29987100
H	-8.66254200	-10.70055800	-0.06339800
H	-8.30728900	-9.66724000	1.34609900
H	-9.65078100	-9.24816000	0.24468900
C	-12.02040600	0.03603300	0.22468000
H	-12.66533600	0.82902700	0.60612600
H	-11.80175700	0.22788400	-0.83382400
H	-12.54212000	-0.92591900	0.31250900

(*M,S*)-3

```
Total energy=                                   -2032.18298493
 Number of imaginary frequencies=                  0
Zero-point correction=                          0.620843 (Hartree/Particle)
 Thermal correction to Energy=                  0.656522
 Thermal correction to Enthalpy=                0.657467
 Thermal correction to Gibbs Free Energy=       0.554494
 Sum of electronic and zero-point Energies=         -2031.562142
 Sum of electronic and thermal Energies=            -2031.526463
 Sum of electronic and thermal Enthalpies=          -2031.525518
 Sum of electronic and thermal Free Energies=       -2031.628491
```

0 1

C	-3.75635100	-0.65909900	0.34735200

C	-3.75625800	0.65913200	-0.34726900
C	-2.75486400	0.95572600	-1.25633500
N	-1.66848100	0.11040800	-1.56634800
C	-0.75014000	-0.18493200	-0.72269200
C	-0.75014700	0.18456000	0.72291800
N	-1.66861400	-0.11052000	1.56652500
C	-2.75502500	-0.95581700	1.25643200
C	-2.80048000	-2.15893000	2.01605300
C	-3.78672600	-3.08165300	1.80545500
C	-4.79649600	-2.85517600	0.83394400
C	-4.78583000	-1.62833600	0.09700400
C	-5.80881700	-3.81464700	0.57861700
C	-6.76938700	-3.59210000	-0.37887000
C	-6.75075500	-2.39258600	-1.12426200
C	-5.78941400	-1.43649400	-0.89144400
C	-4.78566700	1.62845900	-0.09700700
C	-4.79622700	2.85526700	-0.83400000
C	-3.78643100	3.08160700	-1.80551700
C	-2.80025400	2.15879800	-2.01604100
C	-5.78928400	1.43673600	0.89143400
C	-5.80847000	3.81483200	-0.57872300
C	-6.76906800	3.59240400	0.37876400
C	-6.75054600	2.39291700	1.12420600
C	0.51949900	-0.72122400	-1.25947300
C	1.72634500	-0.25396500	-0.70109900
C	1.72633100	0.25357300	0.70127100
C	0.51951300	0.72083700	1.25966500
C	2.87651700	0.25191000	1.56819400
C	2.87748500	1.15575000	2.67103900
C	1.68325900	1.80577500	3.05905400
C	0.50260600	1.49586700	2.43390000
C	4.01403300	-0.65516600	1.45073200
C	5.19976900	-0.38565900	2.19575700
C	5.22193200	0.70109400	3.12751900
C	4.08833600	1.39539400	3.39877900
C	0.50256900	-1.49621000	-2.43372300
C	1.68321000	-1.80596800	-3.05898800
C	2.87741500	-1.15586900	-2.67103500
C	2.87649800	-0.25218200	-1.56805200
C	4.08817400	-1.39525500	-3.39898900
C	5.22171900	-0.70083300	-3.12780600
C	5.19956800	0.38578600	-2.19587400
C	4.01390900	0.65499000	-1.45059200
C	3.96069600	-1.87457300	0.73597300
C	5.04644700	-2.72461800	0.67194700
C	6.24722200	-2.39848500	1.32525200
C	6.31169400	-1.25113700	2.08515700
C	6.31139600	1.25139900	-2.08538500
C	6.24677700	2.39877700	-1.32539700
C	5.04596900	2.72470200	-0.67192500
C	3.96041100	1.87441500	-0.73587500
H	-2.02799200	-2.32082100	2.75810200
H	-3.80630500	-4.00099000	2.38138300
H	-5.80652200	-4.73634100	1.15153000

H	-7.53594500	-4.33491700	-0.56861400
H	-7.50090100	-2.22429100	-1.88898900
H	-5.78813100	-0.52537300	-1.47537300
H	-3.80594100	4.00090500	-2.38151000
H	-2.02774500	2.32058000	-2.75809300
H	-5.78807700	0.52563700	1.47539700
H	-5.80609500	4.73650200	-1.15167200
H	-7.53556400	4.33529300	0.56847100
H	-7.50071400	2.22471700	1.88893200
H	1.70423800	2.48764400	3.90214400
H	-0.44927900	1.85381900	2.80439900
H	6.13939000	0.90357900	3.66985600
H	4.07393700	2.14993700	4.17808000
H	-0.44931100	-1.85417400	-2.80422700
H	1.70416300	-2.48775100	-3.90214800
H	4.07374600	-2.14965600	-4.17842100
H	6.13911800	-0.90308300	-3.67032900
H	3.04159300	-2.16923500	0.25106700
H	4.96444100	-3.65574200	0.12280700
H	7.10201300	-3.06199700	1.25941800
H	7.21163700	-1.01181300	2.64214400
H	7.21127100	1.01241000	-2.64260700
H	7.10146300	3.06244900	-1.25959200
H	4.96387400	3.65585200	-0.12286800
H	3.04132500	2.16887300	-0.25078900

(*M*,*R*)-3

```
Total energy=                                    -2032.20042260
 Number of imaginary frequencies=                   0
Zero-point correction=                           0.621096 (Hartree/Particle)
 Thermal correction to Energy=                    0.656860
 Thermal correction to Enthalpy=                  0.657804
 Thermal correction to Gibbs Free Energy=         0.554311
 Sum of electronic and zero-point Energies=          -2031.579327
 Sum of electronic and thermal Energies=             -2031.543562
 Sum of electronic and thermal Enthalpies=           -2031.542618
 Sum of electronic and thermal Free Energies=        -2031.646112
```

0 1

C	3.63047200	-0.64442400	0.37743000
C	3.63044000	0.64439900	-0.37741200
C	2.65603400	0.90560400	-1.32842400
N	1.56678400	0.05324400	-1.61017100
C	0.69329500	-0.22611000	-0.72067500
C	0.69329700	0.22595100	0.72074700
N	1.56682700	-0.05332700	1.61022600
C	2.65609300	-0.90567200	1.32845200
C	2.73159900	-2.06767300	2.14571600
C	3.71984700	-2.99252600	1.95563800
C	4.70128700	-2.80741200	0.94715400
C	4.66174100	-1.61867900	0.15089400
C	5.71531500	-3.77099200	0.71444600
C	6.65119200	-3.58863100	-0.27529100

C	6.60546100	-2.42666300	-1.07678700
C	5.64162900	-1.46757300	-0.86809600
C	4.66168400	1.61868700	-0.15090800
C	4.70118700	2.80741000	-0.94718400
C	3.71973200	2.99247700	-1.95566300
C	2.73151100	2.06759200	-2.14571200
C	5.64158800	1.46762200	0.86807300
C	5.71518700	3.77102300	-0.71449800
C	6.65107800	3.58870400	0.27523400
C	6.60539100	2.42674400	1.07674500
C	-0.52594600	-0.99281700	-1.04350500
C	-1.70642100	-0.66025200	-0.35557000
C	-1.70640900	0.66016100	0.35567500
C	-0.52590600	0.99272800	1.04355900
C	-2.80338500	1.58072400	0.40251400
C	-2.78294000	2.57579500	1.42738000
C	-1.62825400	2.76743300	2.21913200
C	-0.49074800	2.03480000	1.98506600
C	-3.92082600	1.60416800	-0.53830400
C	-5.06267800	2.40688500	-0.24425700
C	-5.05900400	3.25659500	0.90836800
C	-3.94546100	3.37793800	1.67223900
C	-0.49083600	-2.03484100	-1.98507200
C	-1.62837900	-2.76740400	-2.21917600
C	-2.78305200	-2.57574400	-1.42741100
C	-2.80343500	-1.58076500	-0.40246100
C	-3.94563500	-3.37778200	-1.67233400
C	-5.05917800	-3.25641400	-0.90846100
C	-5.06277700	-2.40682100	0.24425100
C	-3.92089400	-1.60417900	0.53833500
C	-3.89033500	0.95968500	-1.79671100
C	-4.95958700	1.01969900	-2.66867400
C	-6.11875800	1.73604300	-2.32784900
C	-6.15739600	2.42912800	-1.13741500
C	-6.15747300	-2.42906700	1.13741400
C	-6.11878800	-1.73612800	2.32793600
C	-4.95965800	-1.01975600	2.66872200
C	-3.89043500	-0.95969800	1.79674200
H	1.97777500	-2.19884300	2.91259900
H	3.76200600	-3.88238800	2.57495400
H	5.73429800	-4.66348300	1.33156000
H	7.41922100	-4.33431800	-0.44695400
H	7.33662900	-2.28986400	-1.86579200
H	5.62004900	-0.58548400	-1.49470900
H	3.76186300	3.88232600	-2.57499900
H	1.97767600	2.19872500	-2.91259200
H	5.62003800	0.58553900	1.49469700
H	5.73413900	4.66350800	-1.33162100
H	7.41908600	4.33441700	0.44688000
H	7.33657100	2.28997900	1.86574600
H	-1.64114900	3.53594200	2.98441100
H	0.43079400	2.22268700	2.52177900
H	-5.94250500	3.84994100	1.11896500
H	-3.91473300	4.08140500	2.49746100

H	0.43068900	-2.22272100	-2.52181600
H	-1.64133200	-3.53585500	-2.98451200
H	-3.91495500	-4.08120600	-2.49759500
H	-5.94272300	-3.84968400	-1.11909200
H	-3.00192300	0.43015900	-2.10566600
H	-4.89509200	0.51808700	-3.62757000
H	-6.96080400	1.76913100	-3.00988700
H	-7.02279600	3.03137300	-0.88103300
H	-7.02307500	-3.03091700	0.88078000
H	-6.96063100	-1.76943600	3.01020600
H	-4.89510600	-0.51820200	3.62764300
H	-3.00208300	-0.43006300	2.10568700

4.2 Frontier MOs of compounds **4**-**6**

**MOs**	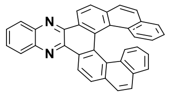 **4a**	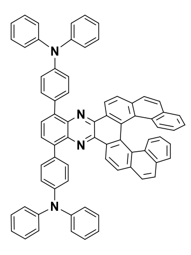 **4b**	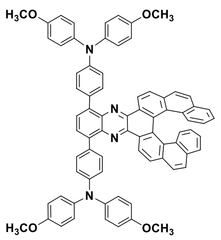 **4c**
**LUMO+1 (eV)**	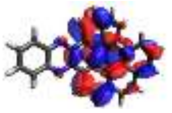 -1.51	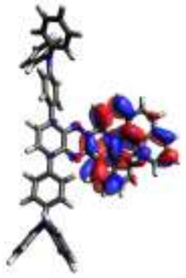 -1.50	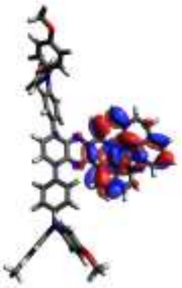 -1.40
**LUMO (eV)**	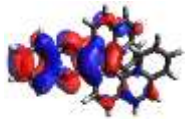 -2.26	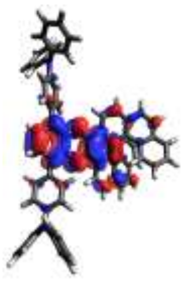 -2.25	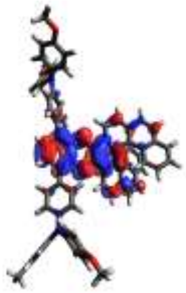 -2.12
**HOMO (eV)**	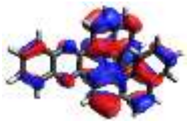 -5.47 (E_g = 3.21 eV)	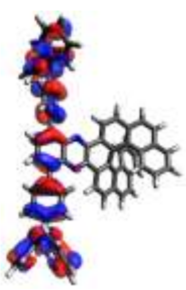 -4.80 (E_g = 2.55 eV)	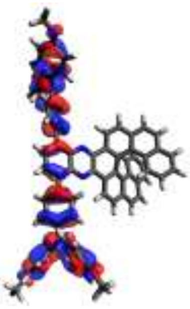 -4.47 (E_g = 2.35 eV)
**HOMO–1 (eV)**	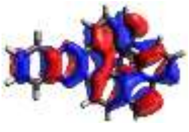 -5.65	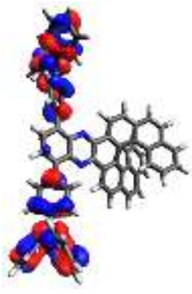 -4.99	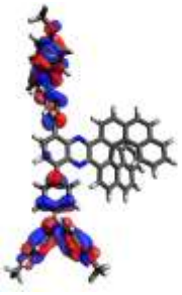 -4.64

MOs	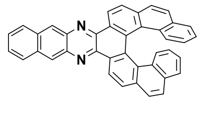<br>**5a**	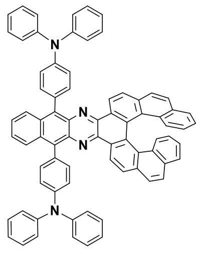<br>**5b**	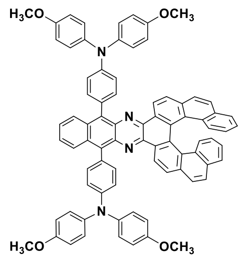<br><br>**5c**
**LUMO+1 (eV)**	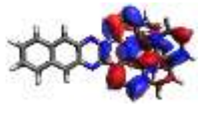<br>-1.53	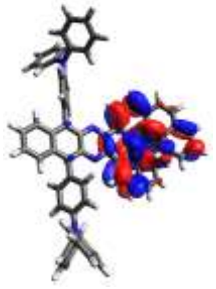<br>-1.50	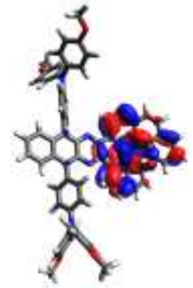<br>-1.40
**LUMO (eV)**	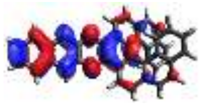<br>-2.52	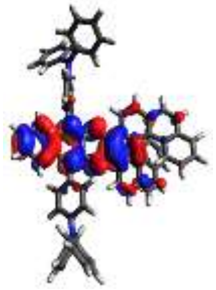<br>-2.48	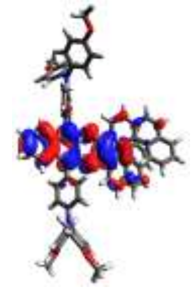<br>-2.34
**HOMO (eV)**	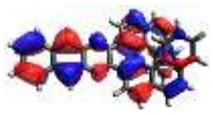<br>-5.32 (E_g = 2.80 eV)	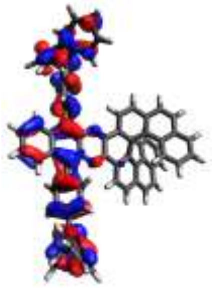<br>-4.82 (E_g = 2.34 eV)	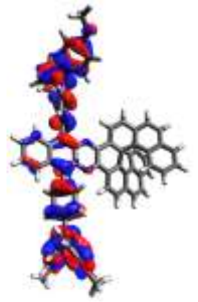<br>-4.50 (E_g = 2.16 eV)
**HOMO–1 (eV)**	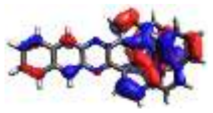<br>-5.62	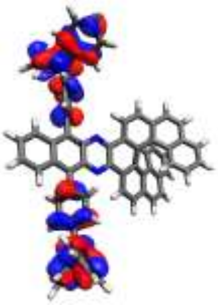<br>-4.97	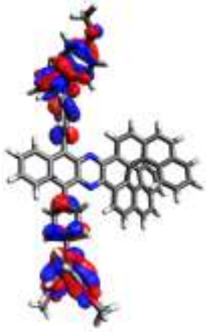<br>-4.61

MOs	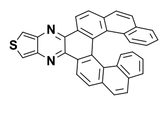<br>**6a**	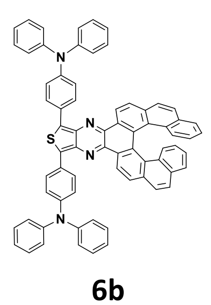<br>**6b**	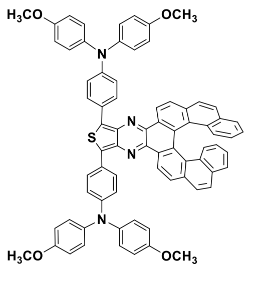<br>**6c**
**LUMO+1 (eV)**	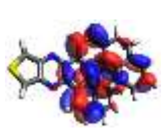<br>-1.52	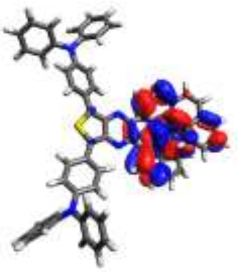<br>-1.51	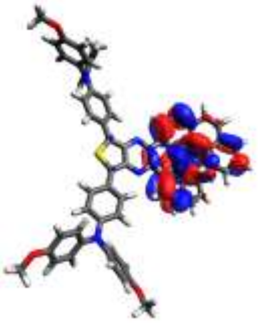<br>-1.40
**LUMO (eV)**	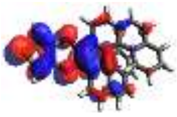<br>-2.52	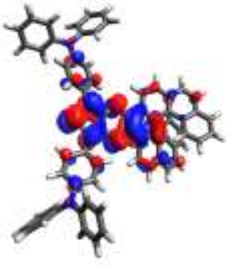<br>-2.54	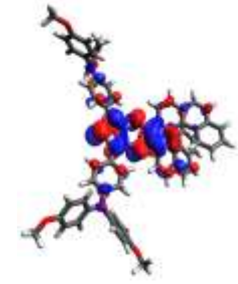<br>-2.38
**HOMO (eV)**	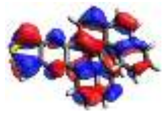<br>-5.39 (E_g = 2.87 eV)	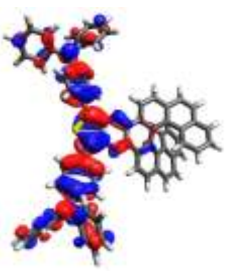<br>-4.53 (E_g = 1.99 eV)	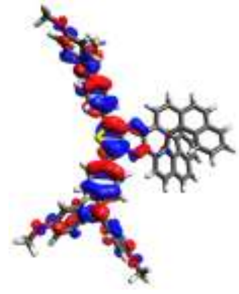<br>-4.24 (E_g = 1.86 eV)
**HOMO–1 (eV)**	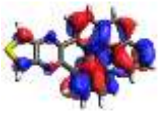<br>-5.64	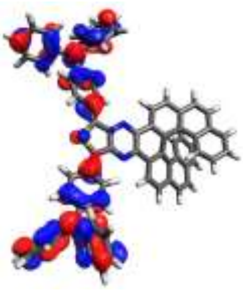<br>-5.08	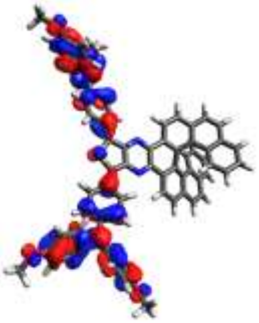<br>-4.73

5. Chiral-HPLC Analysis

Chromatogram

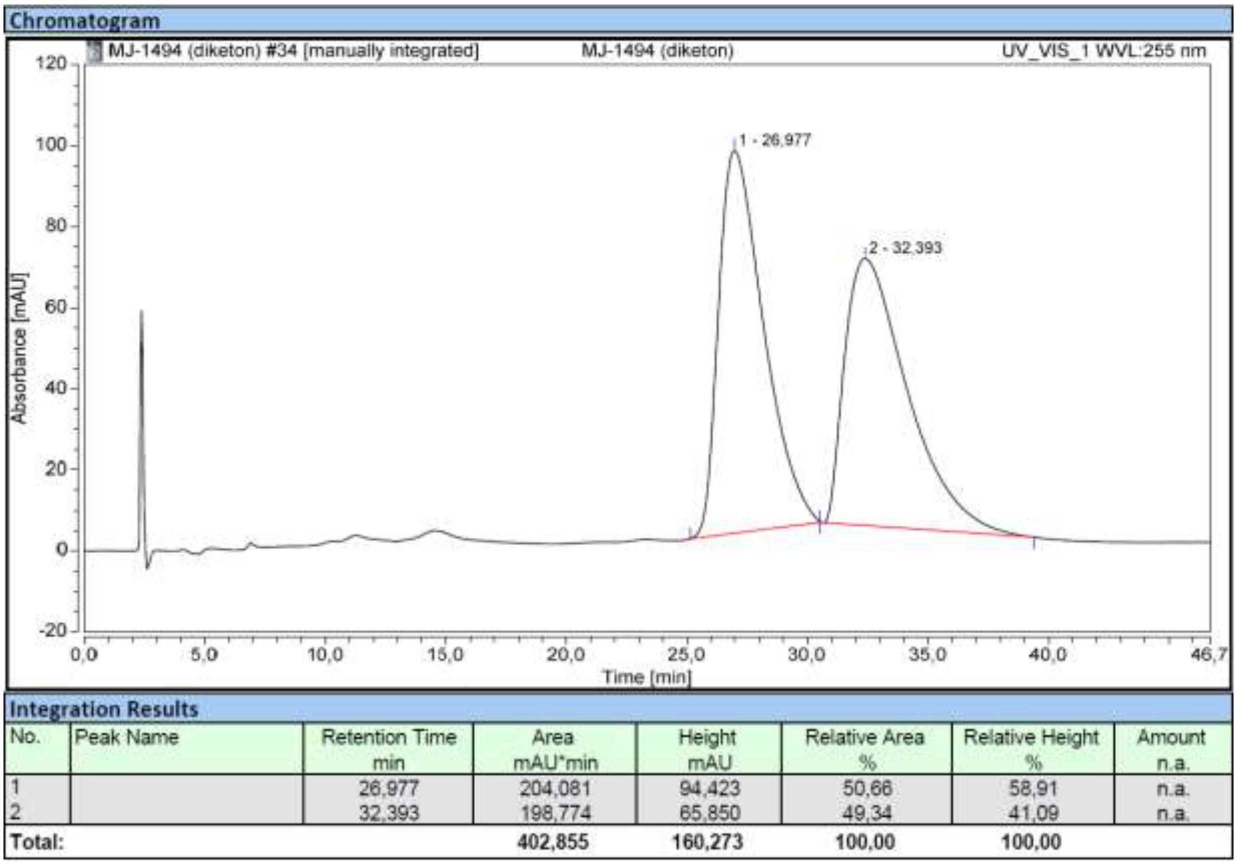

Integration Results

No.	Peak Name	Retention Time min	Area mAU*min	Height mAU	Relative Area %	Relative Height %	Amount n.a.
1		26,977	204,081	94,423	50,66	58,91	n.a.
2		32,393	198,774	65,850	49,34	41,09	n.a.
**Total:**			**402,855**	**160,273**	**100,00**	**100,00**	

Figure S21 HPLC chromatogram of (*rac*)-**1**. Stationary phase: Chiralpak® IB. Mobile phase: Heptane/DCM/IPA (89.5:10: 0.5).

Chromatogram

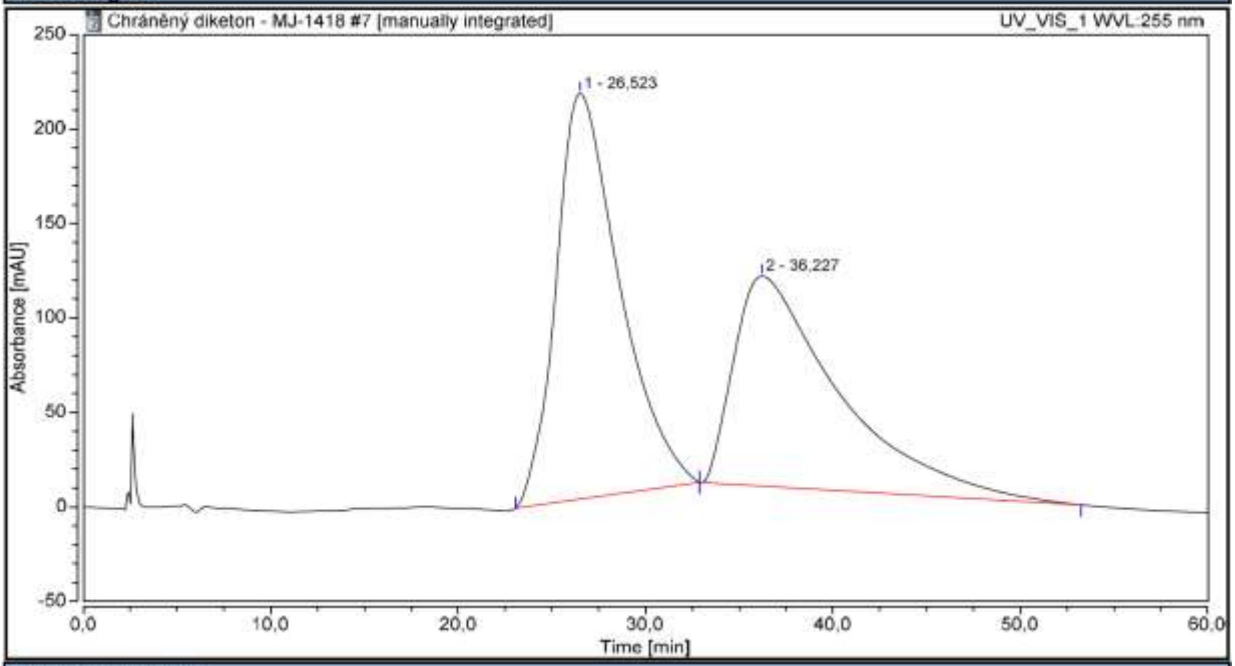

Integration Results

No.	Peak Name	Retention Time min	Area mAU*min	Height mAU	Relative Area %	Relative Height %	Amount n.a.
1		26,523	842,232	214,724	54,00	65,90	n.a.
2		36,227	717,354	111,111	46,00	34,10	n.a.
**Total:**			**1559,586**	**325,835**	**100,00**	**100,00**	

Figure S22 HPLC chromatogram of (*rac*)-**17**. Stationary phase: Chiralpak® IB. Mobile phase: Heptane/MTBE (90:10).

Chromatogram

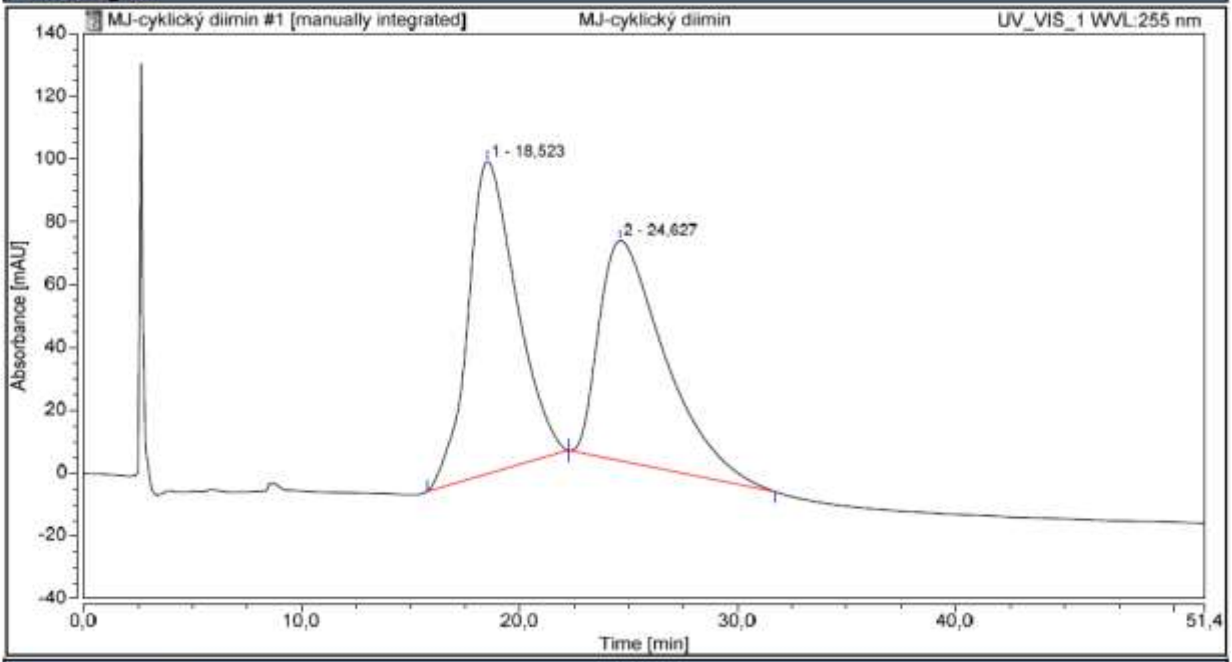

Integration Results

No.	Peak Name	Retention Time min	Area mAU*min	Height mAU	Relative Area %	Relative Height %	Amount n.a.
1		18,523	255,906	99,521	50,93	58,66	n.a.
2		24,627	246,577	70,140	49,07	41,34	n.a.
**Total:**			**502,483**	**169,661**	**100,00**	**100,00**	

Figure S23 HPLC chromatogram of (*S*,*P*)/(*R*,*M*)-**3**. Stationary phase: Chiralpak® IB. Mobile phase: Heptane/IPA (99.5:0.5).

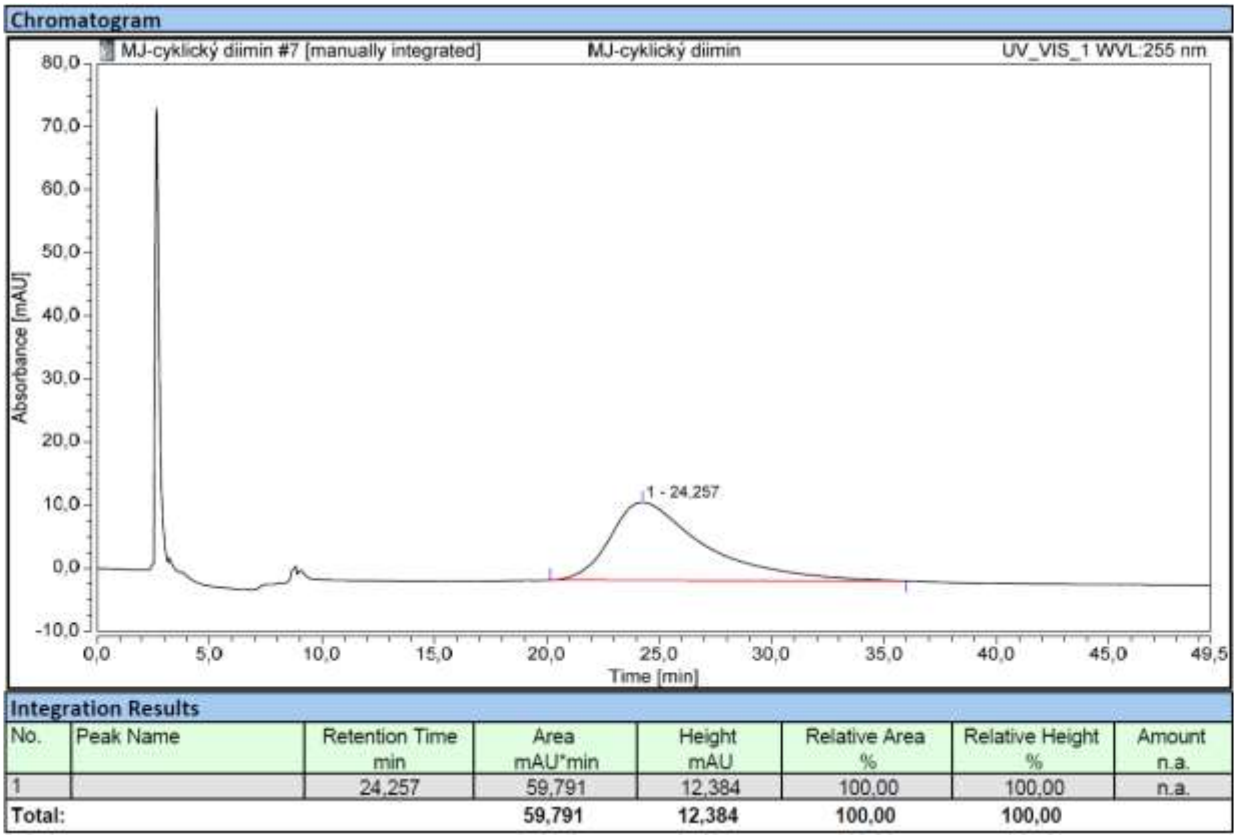

Integration Results

No.	Peak Name	Retention Time min	Area mAU*min	Height mAU	Relative Area %	Relative Height %	Amount n.a.
1		24,257	59,791	12,384	100,00	100,00	n.a.
**Total:**			**59,791**	**12,384**	**100,00**	**100,00**	

Figure S24 HPLC chromatogram of (*S*,*P*)-**3** after **Step 1**. Stationary phase: Chiralpak® IB. Mobile phase: Heptane/IPA (99.5:0.5).

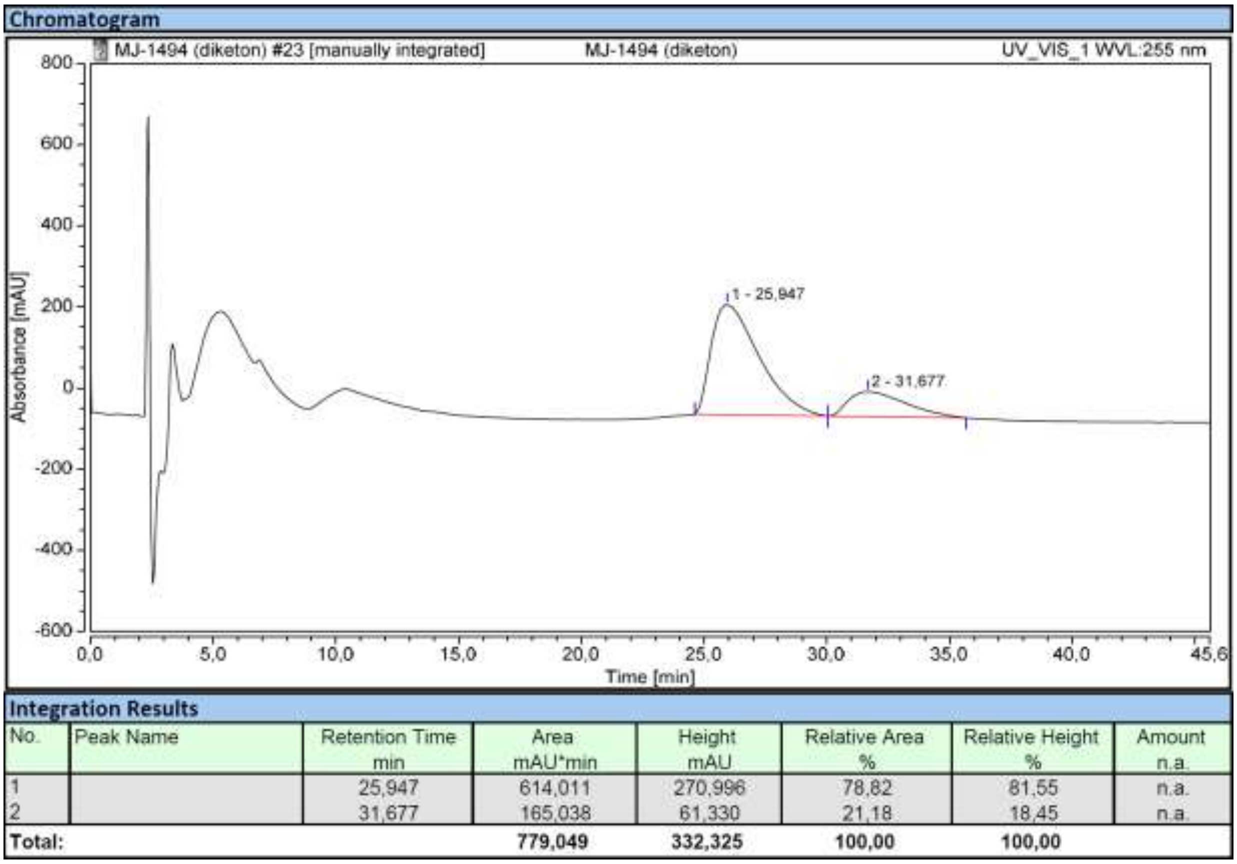

Integration Results

No.	Peak Name	Retention Time min	Area mAU*min	Height mAU	Relative Area %	Relative Height %	Amount n.a.
1		25,947	614,011	270,996	78,82	81,55	n.a.
2		31,677	165,038	61,330	21,18	18,45	n.a.
**Total:**			**779,049**	**332,325**	**100,00**	**100,00**	

Figure S25 HPLC chromatogram of enantioenriched (*M*)-**1** after **Step 1**. Stationary phase: Chiralpak® IB. Mobile phase: Heptane/DCM/IPA (89.5:10: 0.5).

Chromatogram

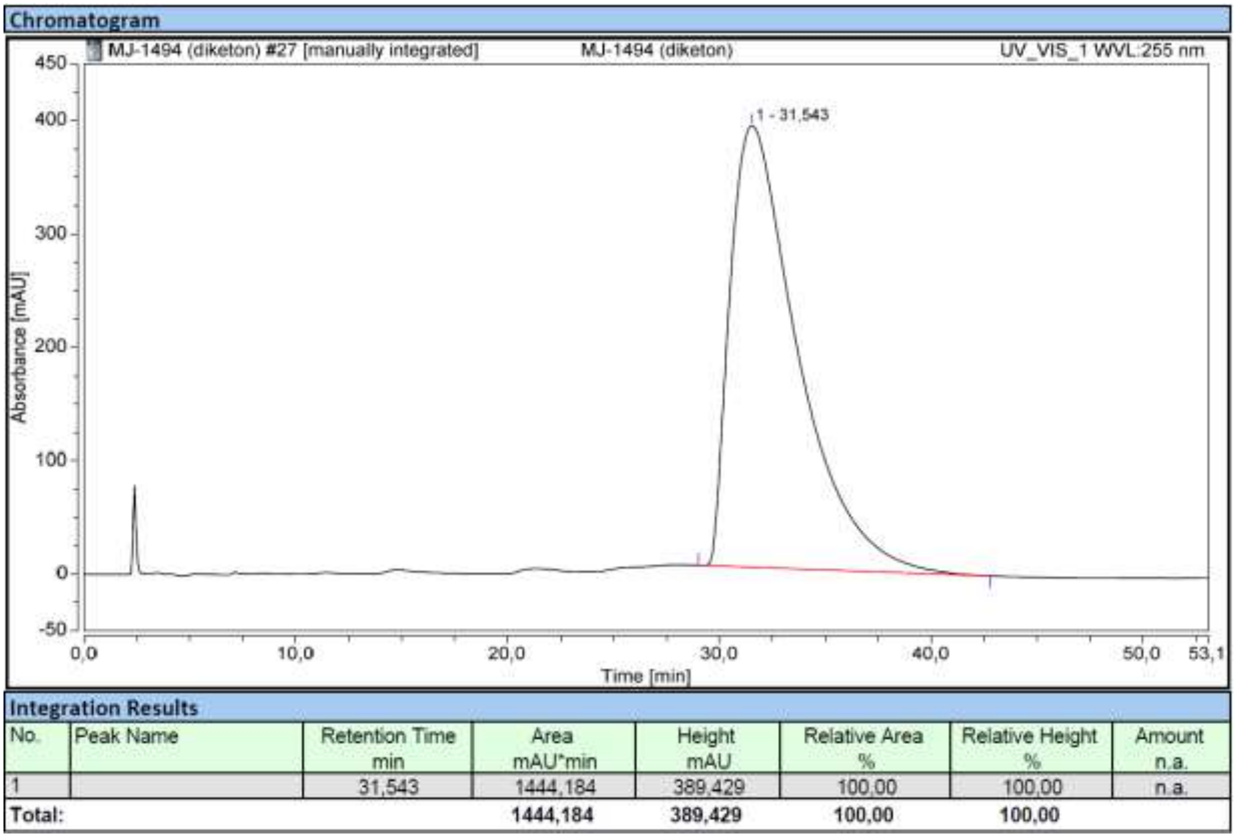

Integration Results

No.	Peak Name	Retention Time<br>min	Area<br>mAU*min	Height<br>mAU	Relative Area<br>%	Relative Height<br>%	Amount<br>n.a.
1		31,543	1444,184	389,429	100,00	100,00	n.a.
**Total:**			**1444,184**	**389,429**	**100,00**	**100,00**	

Figure S26 HPLC chromatogram of (*P*)-**1** after **Step 2**. Stationary phase: Chiralpak® IB. Mobile phase: Heptane/DCM/IPA (89.5:10: 0.5).

Chromatogram

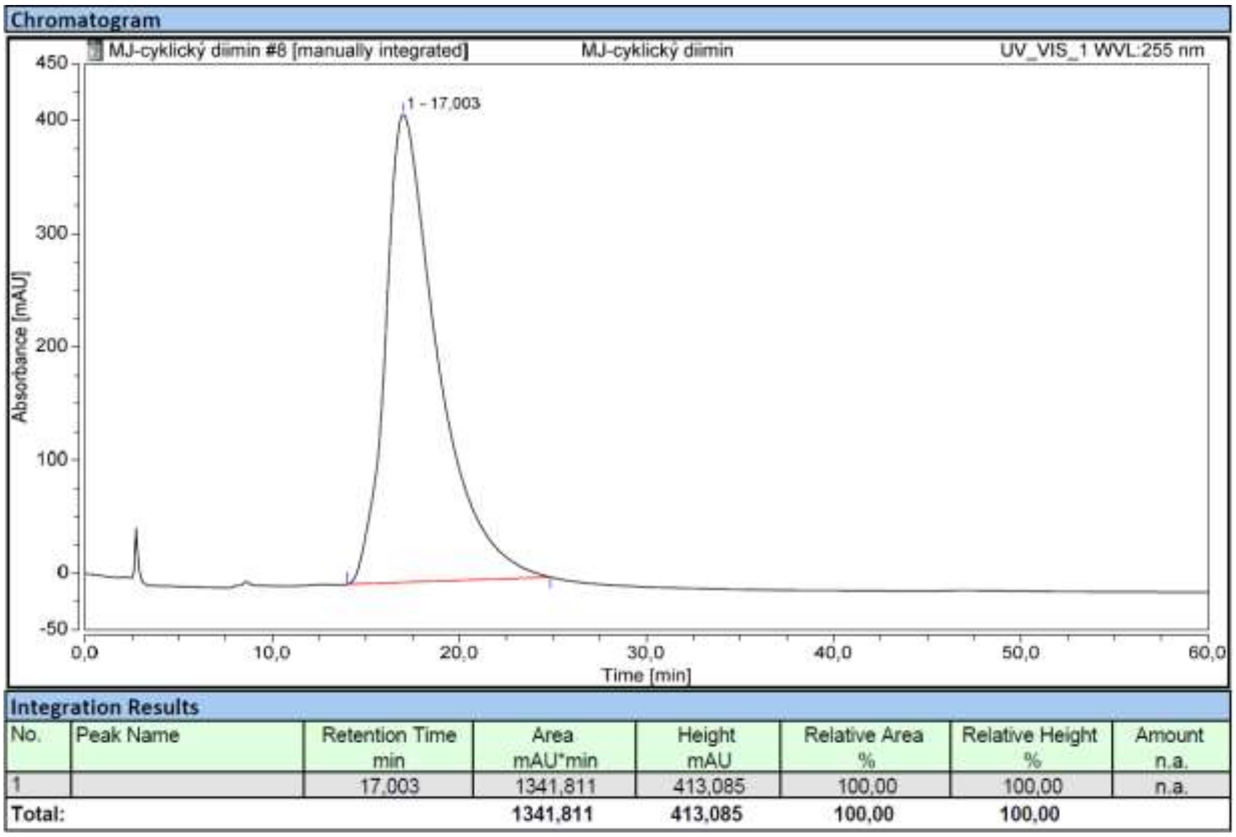

Integration Results

No.	Peak Name	Retention Time<br>min	Area<br>mAU*min	Height<br>mAU	Relative Area<br>%	Relative Height<br>%	Amount<br>n.a.
1		17,003	1341,811	413,085	100,00	100,00	n.a.
**Total:**			**1341,811**	**413,085**	**100,00**	**100,00**	

Figure S27 HPLC chromatogram of (*R*,*M*)-**3** after **Step 3**. Stationary phase: Chiralpak® IB. Mobile phase: Heptane/IPA (99.5:0.5).

Chromatogram

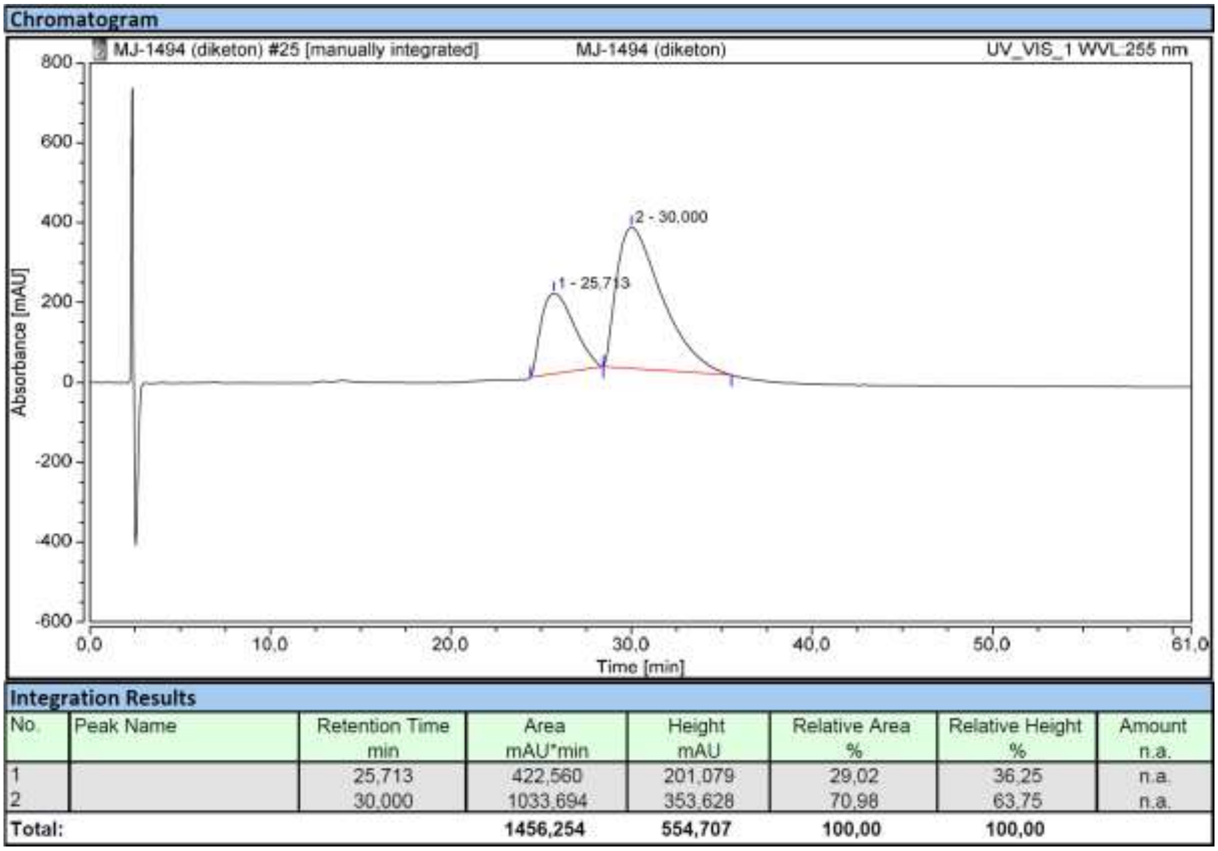

Integration Results

No.	Peak Name	Retention Time min	Area mAU*min	Height mAU	Relative Area %	Relative Height %	Amount n.a.
1		25,713	422,560	201,079	29,02	36,25	n.a.
2		30,000	1033,694	353,628	70,98	63,75	n.a.
**Total:**			**1456,254**	**554,707**	**100,00**	**100,00**	

Figure S28 HPLC chromatogram of enantioenriched (*P*)-**1** after **Step 3**. Stationary phase: Chiralpak® IB. Mobile phase: Heptane/DCM/IPA (89.5:10: 0.5).

Chromatogram

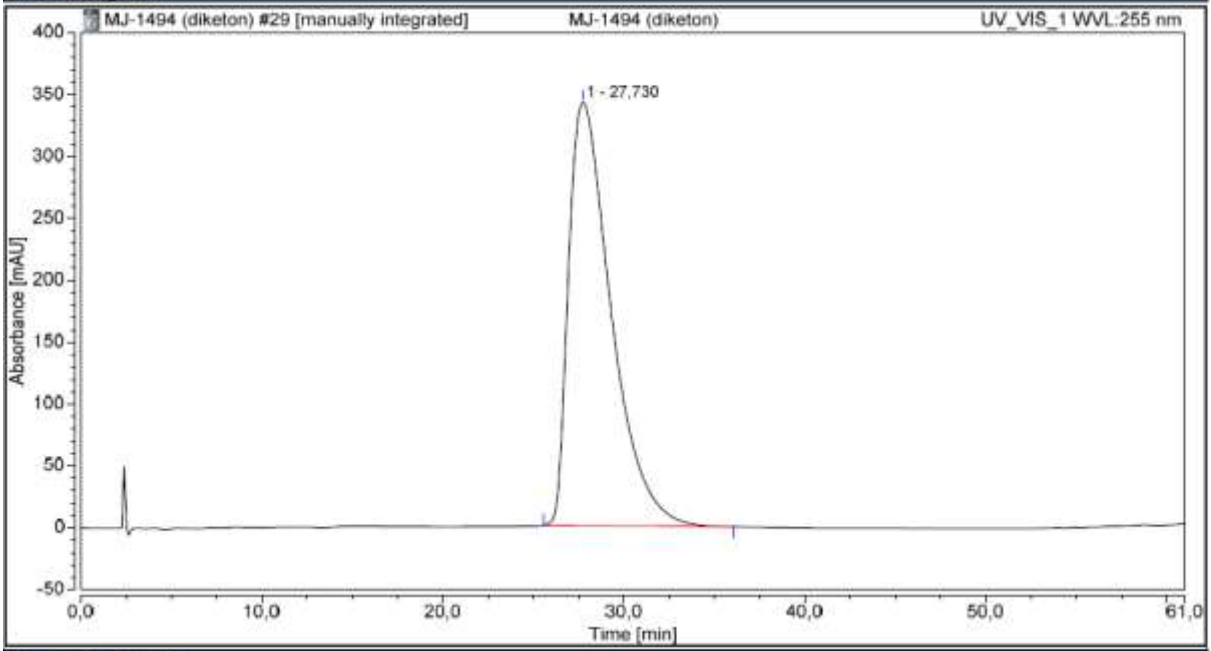

Integration Results

No.	Peak Name	Retention Time min	Area mAU*min	Height mAU	Relative Area %	Relative Height %	Amount n.a.
1		27,730	931,421	342,105	100,00	100,00	n.a.
**Total:**			**931,421**	**342,105**	**100,00**	**100,00**	

Figure 29 HPLC chromatogram of (*M*)-**1** after **Step 4** . Stationary phase: Chiralpak® IB. Mobile phase: Heptane/DCM/IPA (89.5:10:0.5).

6. X-ray crystallographic data

Diffraction data were collected on a Bruker D8 VENTURE Kappa Duo PHOTON 100 CMOS with the monochromated Mo/Cu-Kα radiation. The structures were solved by direct methods (SHELXT[8]) and refined by full-matrix least-squares on F^2 values (SHELXL). All heavy atoms were refined anisotropically. Hydrogen atoms were usually localized from the expected geometry and difference electron density maps. The hydrogen atoms were fixed into idealized positions (riding model) and assigned temperature factors Hiso(H) = 1.2 Ueq(pivot atom). ORTEP-3[9] was used for structure presentation.

The crystallographic data for the structures reported in this paper have been deposited with the Cambridge Crystallographic Data Centre as supplementary publication. Copies of the data can be obtained free of charge on application to CCDC, e-mail: deposit@ccdc.cam.ac.uk.

X-ray data of (*P*)-(**1**): $C_{30}H_{16}O_2{\cdot}CHCl_3$, M=527.80 g.mol^{-1}, orthorhombic system, space group *P* $2_12_12_1$, a=13.8514(5), b=15.0639(6), c=33.8305(13) Å, Z=12, V=7058.9(5) Å^3, Dc=1.49 g.cm^{-3}, μ(Cu Kα)=3.761 mm^{-1}, T=120 K, crystal dimensions of 0.07 x 0.10 x 0.44 mm, dark red prism. The independent part of the lattice cell is formed by three molecules of **1** and three solvent molecules (Figure S21). It was inevitable to model a disorder of all three solvent molecules. The structure model converged to the final R=0.0302 and R_w=0.0750 using 13808 independent reflections for 1021 refined parameters (θ_{max}=72.39°). The Flack parameter converged to 0.002(4) for *P* isomer. CCDC registration number 2264112.

X-ray data of (*S*,*P*)/(*R*,*M*)-(**3**): $C_{50}H_{28}N_2$, M=656.74 g.mol^{-1}, triclinic system, space group *P-1*, a=10.3127(3), b=11.8284(3), c=13.8257(4) Å, α=87.745(1), β=76.743(1), γ=85.738(1) °,Z=2, V=1636.58(8) Å^3, Dc=1.333 g.cm^{-3}, μ(Cu Kα)=0.594 mm^{-1}, T=120 K, crystal dimensions of 0.06 x 0.08 x 0.18 mm, orange prism. The independent part of the lattice cell is formed by one molecule of **3** (Figure S22). The structure converged to the final R=0.0396 and R_w=0.1060 using 6505 independent reflections for 469 refined parameters (θ_{max}=73.33°). CCDC registration number 2264111.

X-ray data of (*rac*)-**5c**: $C_{80}H_{56}N_4O_4$, M=1137.29 g.mol^{-1}, triclinic system, space group *P-1*, a=11.7030(9), b=14.0339(10), c=18.5894(12) Å, α=108.435(2), β=93.395(2), γ=93.018(2) °, Z=2, V=2883.1(4) Å^3, Dc=1.310 g.cm^{-3}, μ(Mo Kα)=0.081 mm^{-1}, T=150 K, crystal dimensions of 0.16 x 0.28 x 0.28 mm, dark red prism. The independent part of the lattice cell is formed by one molecule of **5c** (Figure S23). It was inevitable to model a disorder of two phenyl methoxy groups. The structure converged to the final R=0.0601 and R_w=0.1596 using 11629 independent reflections for 937 refined parameters (θ_{max}=27.53°). CCDC registration number 2264113.

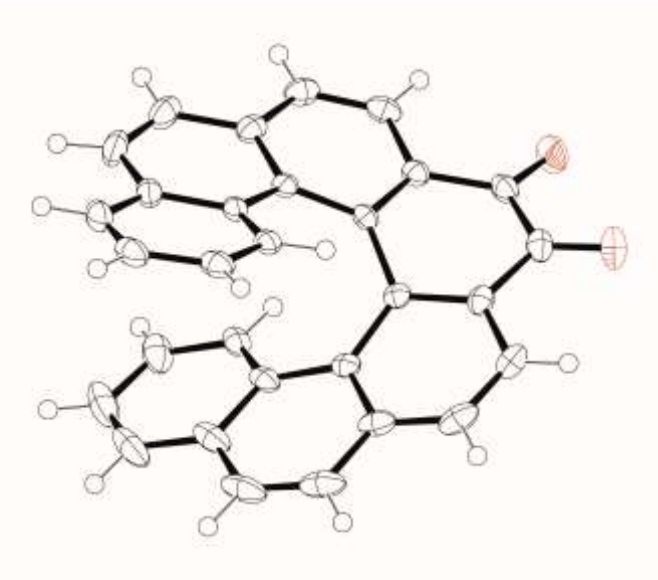

Figure S30. ORTEP projection of one molecule found in the crystal structure of **1**. The rest of the molecules forming an independent part of the unit cell was remove for clarity. Thermal ellipsoids are shown with 50% probability.

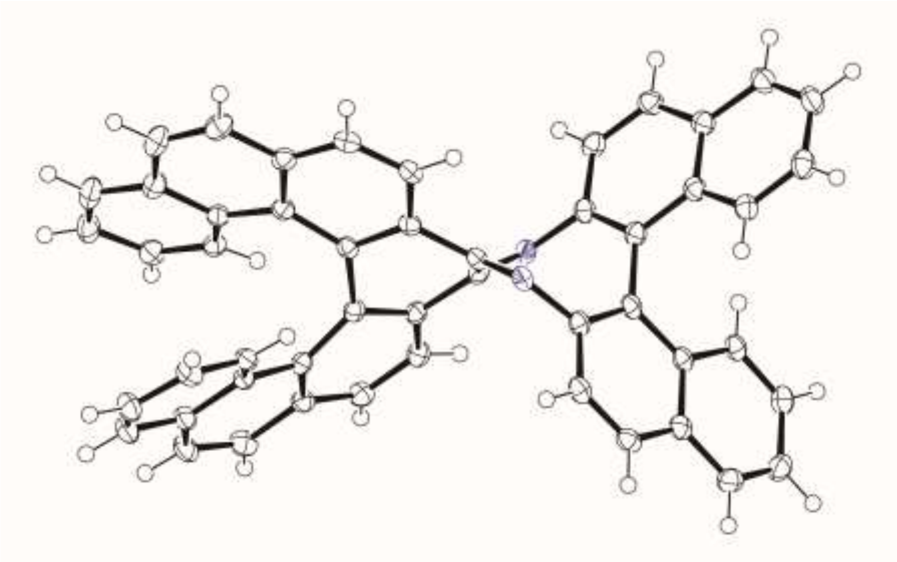

Figure S31. ORTEP projection of the crystal structure of **3**. Thermal ellipsoids are shown with 50% probability.

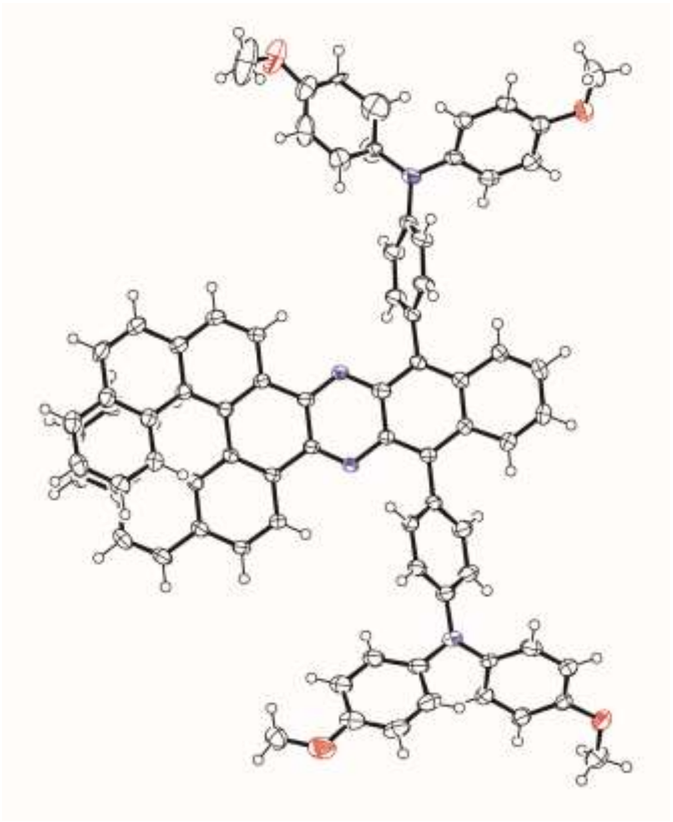

Figure S32. ORTEP projection of the crystal structure of **5c**. The disordered parts of the molecule were removed for clarity. Thermal ellipsoids are shown with 50% probability.

7. UV/Vis spectra, Fluorescence, Circular Dichroism and g_{abs} spectra

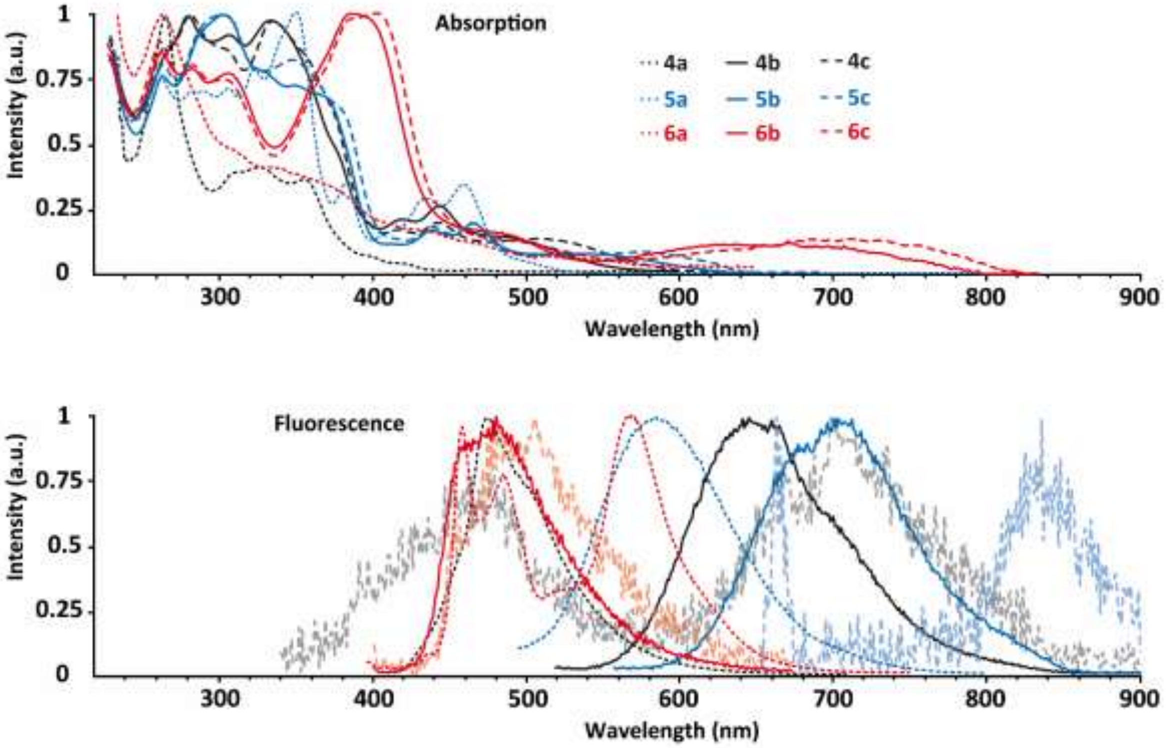

Figure S33 *Normalized absorption (5 × 10^{-6} M, CH_2Cl_2) and fluorescence (5 × 10^{-6} M, CH_2Cl_2) spectra of series of compounds **4a**-**c**, **5a**-**c** and **6a**-**c**.*

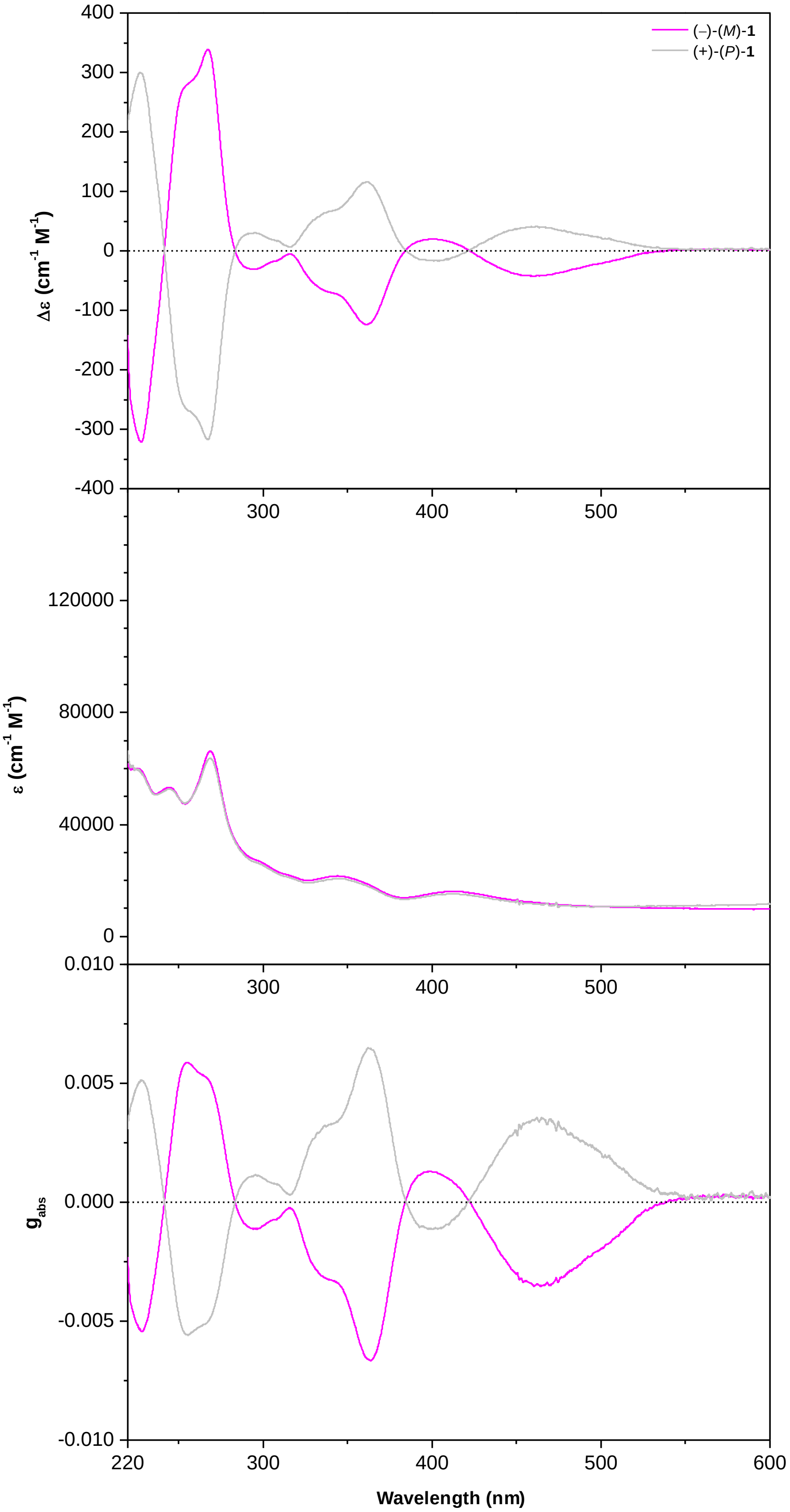

Figure S34 *CD spectra, UV/Vis spectra, and* g_{abs} *of* (*M*)-**1** and (*P*)-**1**.

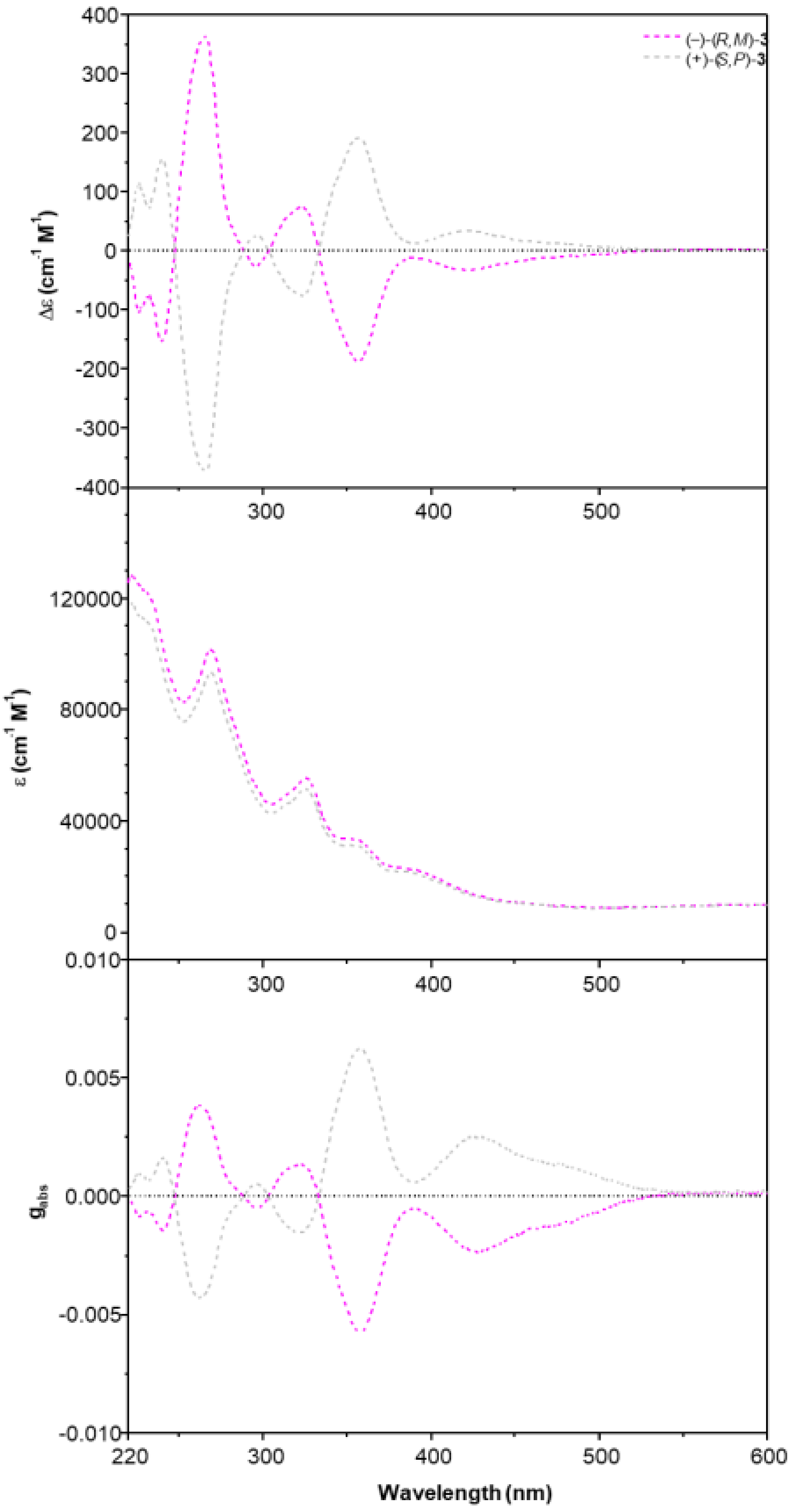

Figure S35 *CD spectra, UV/Vis spectra, and* g_{abs} *of* (*R*,*M*)-**3** and (*S*,*P*)-**3**.

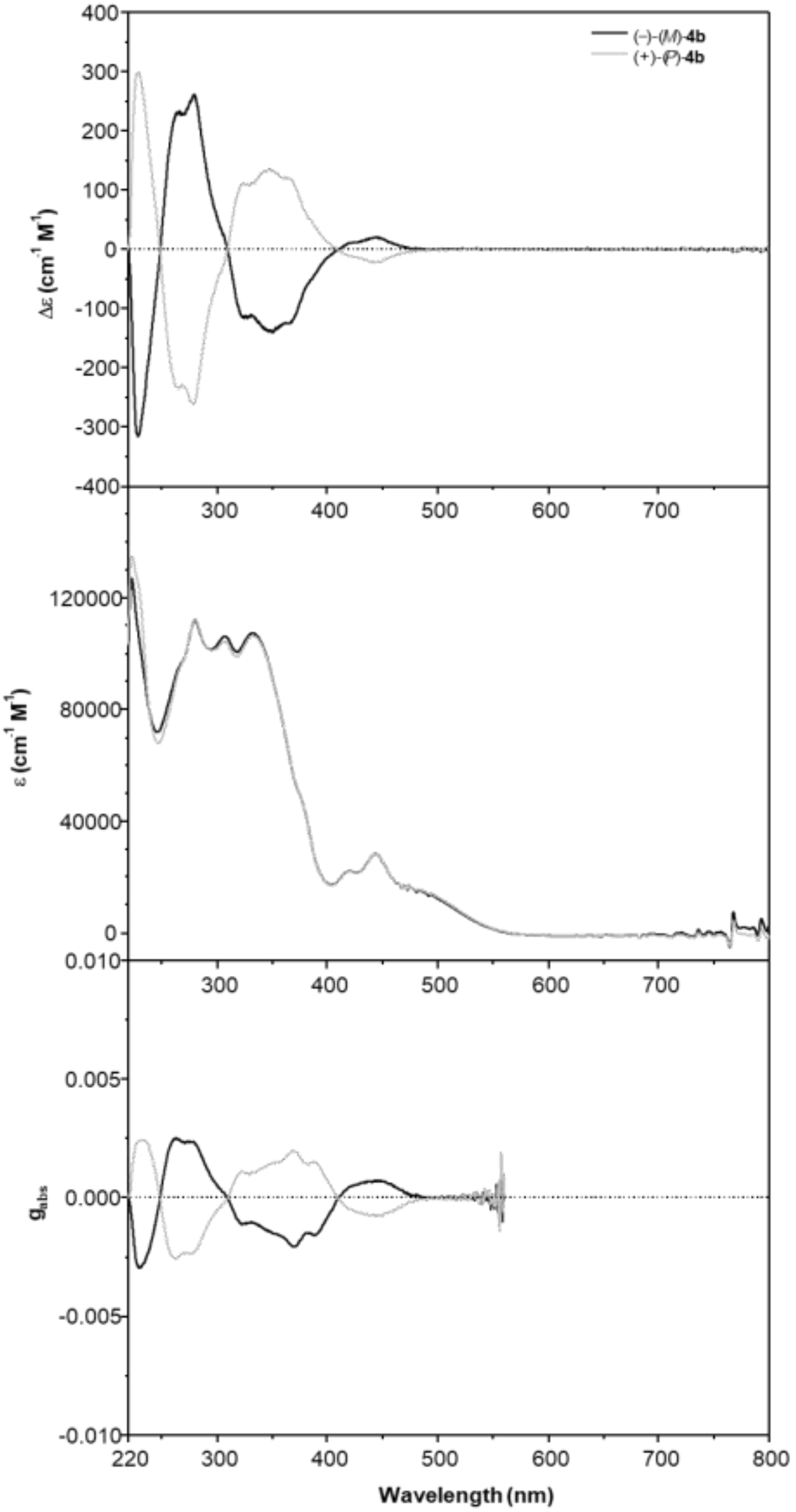

Figure S36 *CD spectra, UV/Vis spectra, and g_{abs} of* (*M*)-**4b** and (*P*)-**4b**.

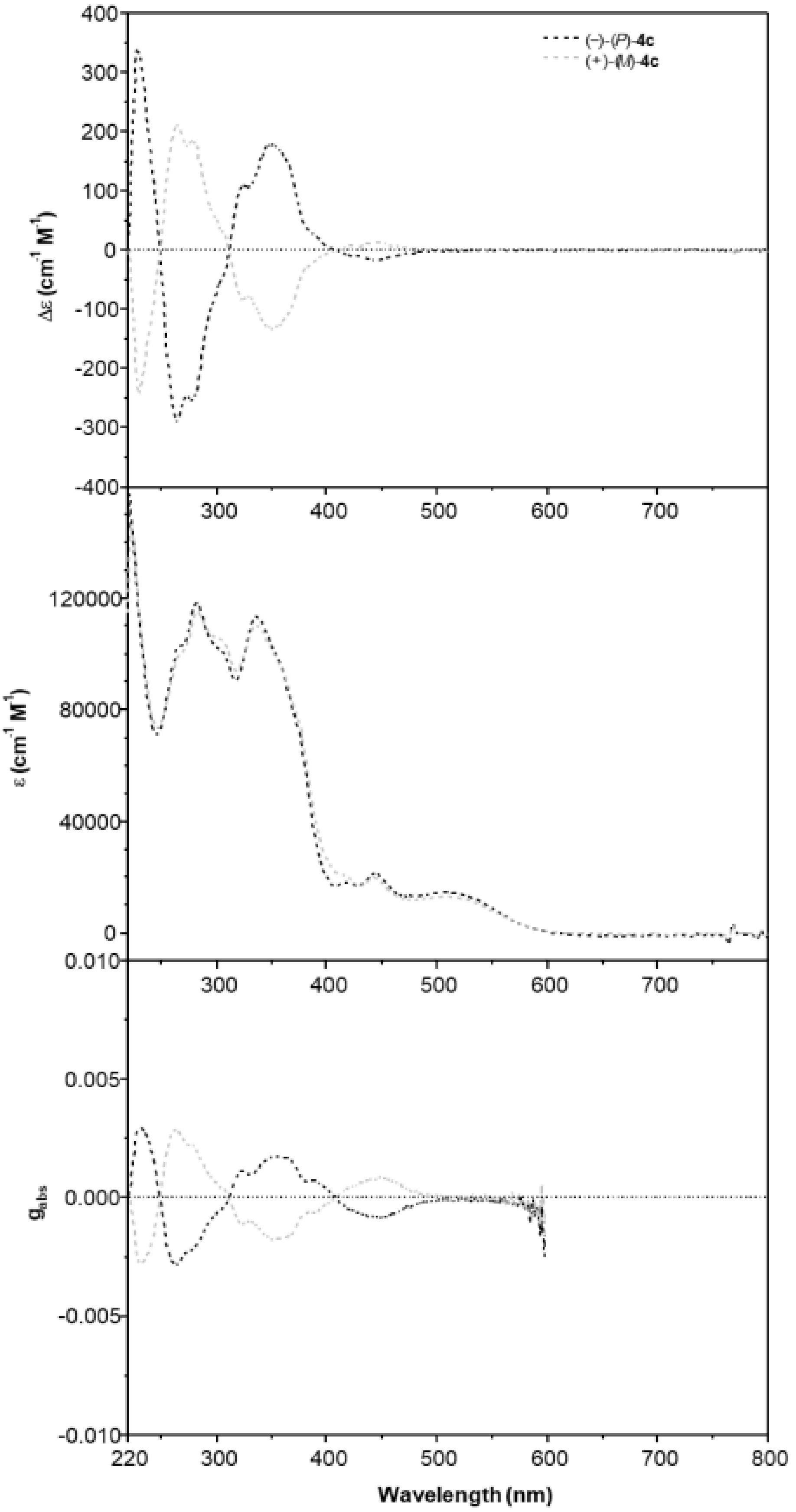

Figure S37 *CD spectra, UV/Vis spectra, and g_{abs} of* (*M*)-**4c** and (*P*)-**4c**.

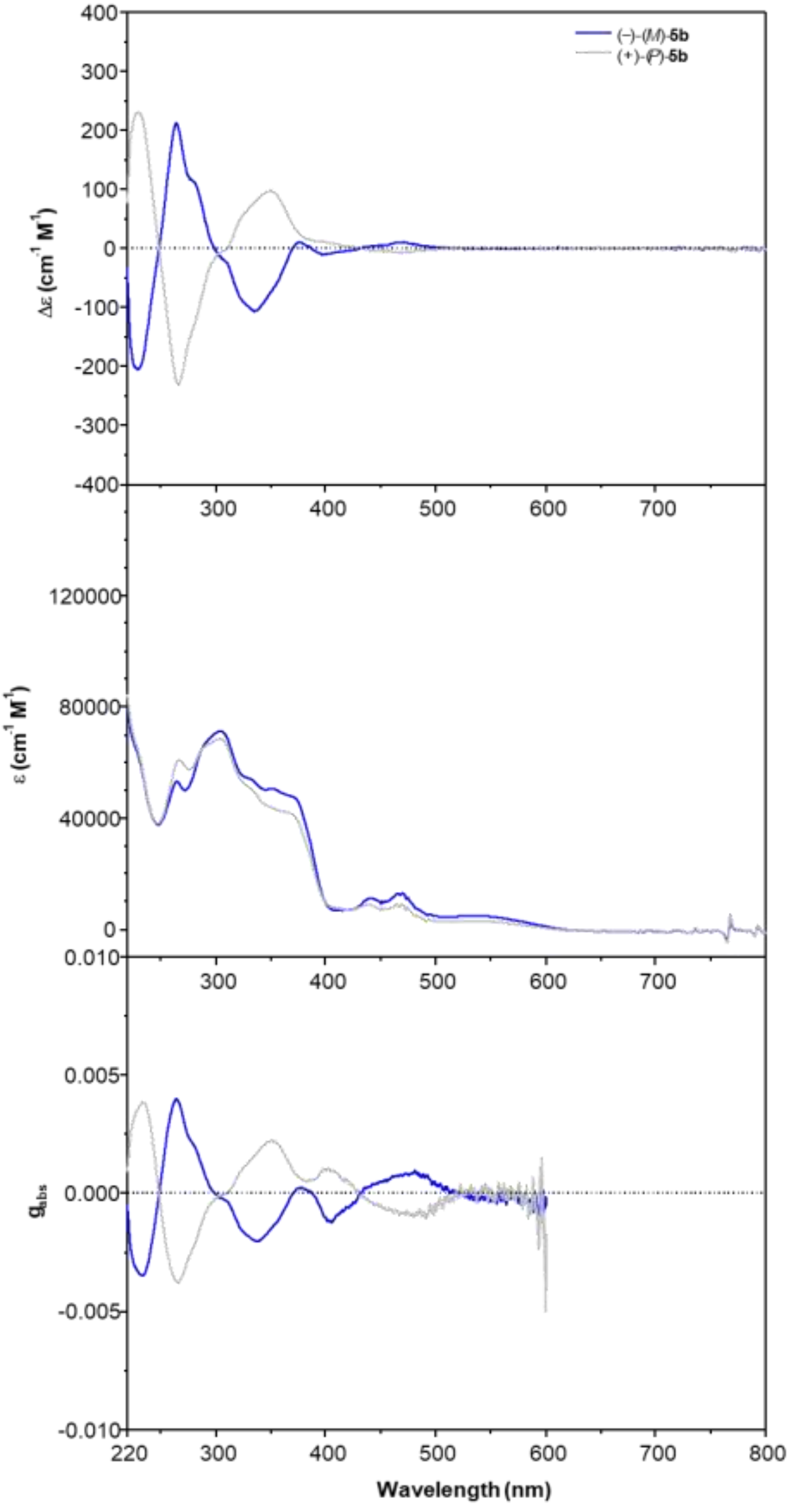

***Figure S38** CD spectra, UV/Vis spectra, and g_{abs} of* (*M*)-**5b** and (*P*)-**5b**.

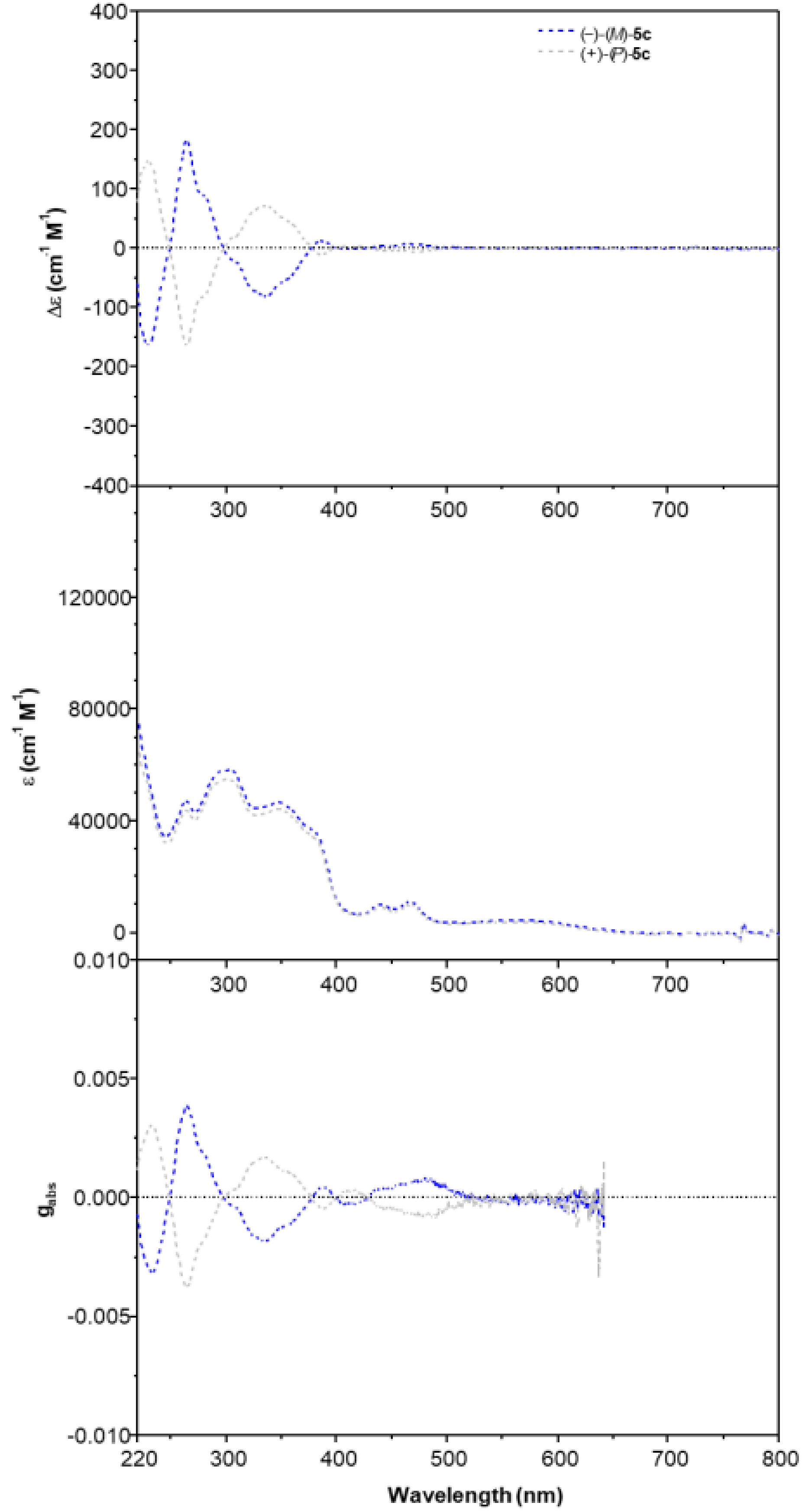

Figure S39 CD spectra, UV/Vis spectra, and g_{abs} of (*M*)-***5c*** *and* (*P*)-***5c***.

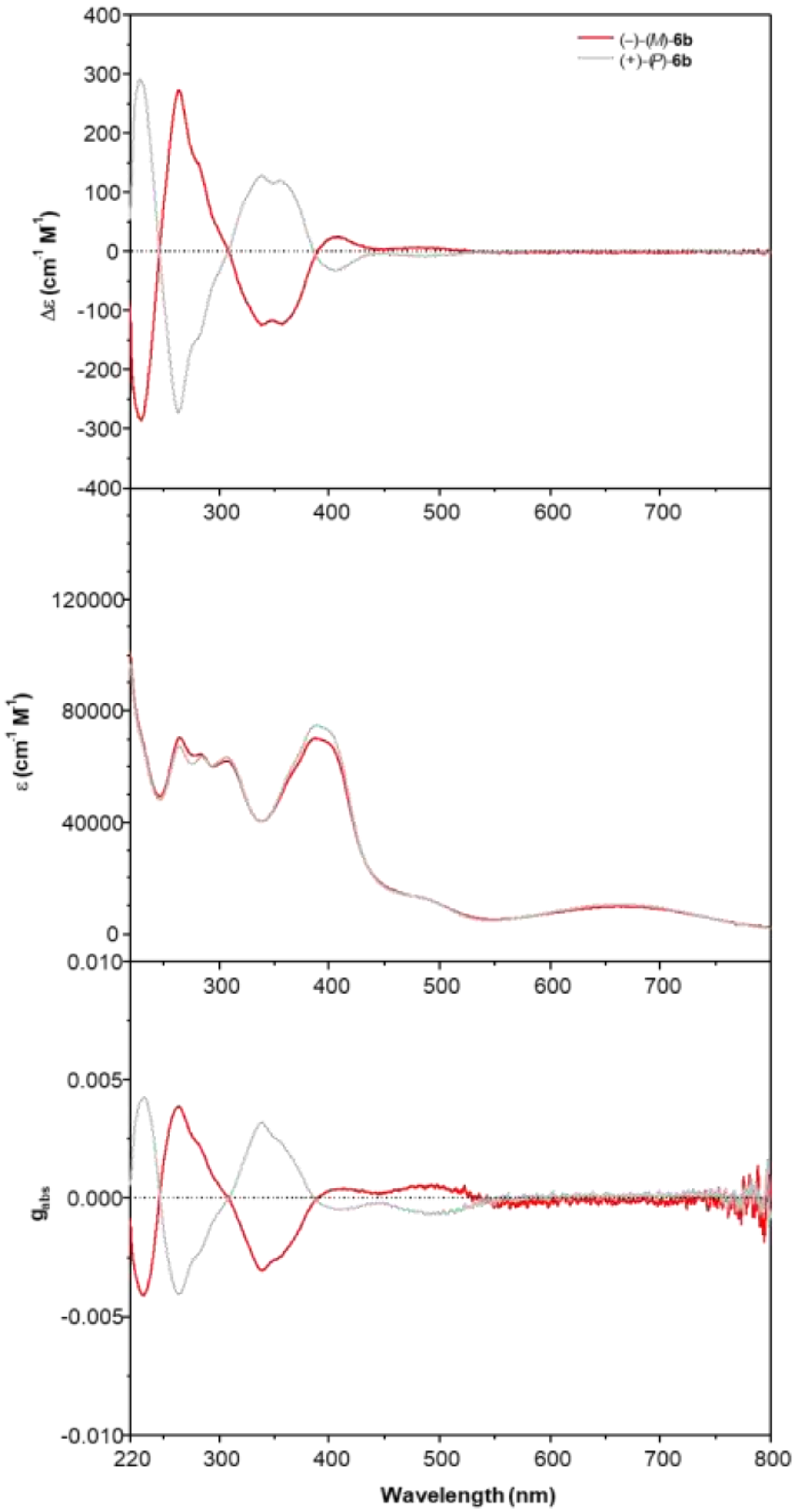

Figure S40 CD spectra, UV/Vis spectra, and g_{abs} of (*M*)-***6b*** *and* (*P*)-***6b***.

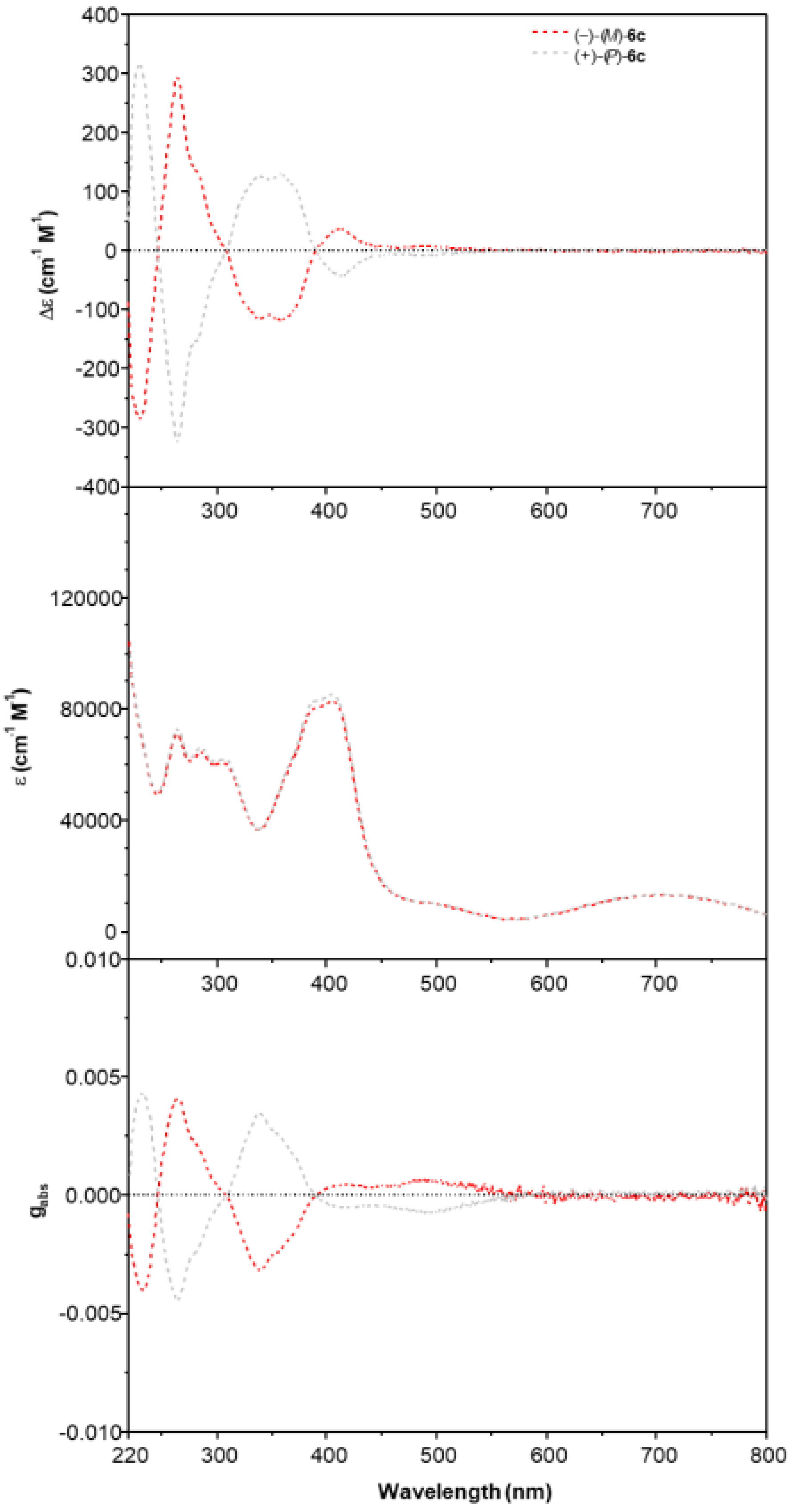

Figure S41 CD spectra, UV/Vis spectra, and g_{abs} of (*M*)-***6c*** *and* (*P*)-***6c***.

8. References

(1) Funchien, P.; Chasing, P.; Sudyoadsuk, T.; Promarak, V. A Highly Efficient near Infrared Organic Solid Fluorophore Based on Naphthothiadiazole Derivatives with Aggregation-Induced Emission Enhancement for a Non-Doped Electroluminescent Device. *Chem Commun* **2020**, *56* (46), 6305–6308. https://doi.org/10.1039/D0CC01648A.

(2) Li, Q.; Li, J.; Yang, R.; Deng, L.; Gao, Z.; Liu, D. Novel Red-Emitting Thieno-[3,4-b]-Pyrazine Derivatives Suitable for Vacuum Evaporation and Solution Method to Fabricate Non-Doped OLEDs. *Dyes Pigm* **2012**, *92* (1), 674–680. https://doi.org/10.1016/j.dyepig.2011.05.029.

(3) Jakubec, M.; Hansen-Troøyen, S.; Císařová, I.; Sýkora, J.; Storch, J. Photochemical Oxidation Specific to Distorted Aromatic Amines Providing Ortho-Diketones. *Org Lett* **2020**, *22* (10), 3905–3910. https://doi.org/10.1021/acs.orglett.0c01190.

(4) Seo, T.; Toyoshima, N.; Kubota, K.; Ito, H. Tackling Solubility Issues in Organic Synthesis: Solid-State Cross-Coupling of Insoluble Aryl Halides. *J Am Chem Soc* **2021**, *143* (16), 6165–6175. https://doi.org/10.1021/jacs.1c00906.

(5) Liu, Y.; Wang, Y.; Song, X.; Wang, X.; Zhu, H.; Zhang, J.; Bai, J.; Redshaw, C.; Ni, X.-L.; Feng, X.; Wang, D.; Tang, B. Z. Tunable Fluorescence Emission for Multi-Color Light-Emitting Diodes and Voice-Activated Intelligent Lighting Applications. *J Mater Chem C* **2022**, *10* (22), 8783–8790. https://doi.org/10.1039/D2TC00936F.

(6) Liu, T.; Zhu, L.; Zhong, C.; Xie, G.; Gong, S.; Fang, J.; Ma, D.; Yang, C. Naphthothiadiazole-Based Near-Infrared Emitter with a Photoluminescence Quantum Yield of 60% in Neat Film and External Quantum Efficiencies of up to 3.9% in Nondoped OLEDs. *Adv Funct Mater* **2017**, *27* (12), 1606384. https://doi.org/10.1002/adfm.201606384.

(7) Yzeiri, X.; Calamante, M.; Dessì, A.; Franchi, D.; Pucci, A.; Ventura, F.; Reginato, G.; Zani, L.; Mordini, A. Synthesis and Spectroscopic Characterization of Thienopyrazine-Based Fluorophores for Application in Luminescent Solar Concentrators (LSCs). *Molecules* **2021**, *26* (18), 5428. https://doi.org/10.3390/molecules26185428.

(8) Sheldrick, G. M. Crystal Structure Refinement with SHELXL. *Acta Crystallogr C Struct Chem* **2015**, *71* (1), 3–8. https://doi.org/10.1107/S2053229614024218.

(9) Farrugia, L. J. ORTEP -3 for Windows - a Version of ORTEP -III with a Graphical User Interface (GUI). *J Appl Crystallogr* **1997**, *30* (5), 565–565. https://doi.org/10.1107/S0021889897003117.

(10) Frisch, M. J.; Trucks, G. W.; Schlegel, H. B.; Scuseria, G. E.; Robb, M. A.; Cheeseman, J. R.; Scalmani, G.; Barone, V.; Mennucci, B.; Petersson, G. A.; Nakatsuji, H.; Caricato, M.; Li, X.; Hratchian, H. P.; Izmaylov, A. F.; Bloino, J.; Zheng, G.; Sonnenberg, J. L.; Hada, M.; et al. Gaussian 09. *Gaussian 09, Revision B.01*. Gaussian Inc.: Wallingford CT 2009.

(11) Becke, A. D. Density-Functional Thermochemistry. III. The Role of Exact Exchange. *J Chem Phys* **1993**, *98* (7), 5648–5652. https://doi.org/10.1063/1.464913.

(12) Perdew, J. P.; Wang, Y. Accurate and Simple Analytic Representation of the Electron-Gas Correlation Energy. *Phys Rev B* **1992**, *45* (23), 13244–13249. https://doi.org/10.1103/PhysRevB.45.13244.
