# Supplementary figures and images for: Resolution of 9,10-Diketo[7]helicene and Its Use in One-Step Preparation of Helicene-Based D–A–D Push–Pull Systems
Source: J Org Chem. 2024 May 28;89(11):7495–502. doi: 10.1021/acs.joc.4c00135 (PMC11165575; doi:10.1021/acs.joc.4c00135)

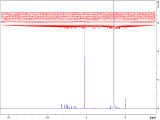

Supplement: Supplementary file 2 — jo4c00135_si_002.zip [file jo4c00135_si_002.zip › Fidy/1/21/pdata/1/thumb.png]

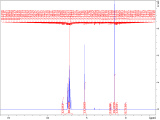

Supplement: Supplementary file 2 — jo4c00135_si_002.zip [file jo4c00135_si_002.zip › Fidy/13b/30/pdata/1/thumb.png]

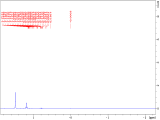

Supplement: Supplementary file 2 — jo4c00135_si_002.zip [file jo4c00135_si_002.zip › Fidy/13c/10/pdata/1/thumb.png]

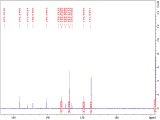

Supplement: Supplementary file 2 — jo4c00135_si_002.zip [file jo4c00135_si_002.zip › Fidy/13c/11/pdata/1/thumb.png]

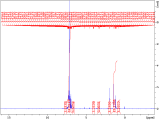

Supplement: Supplementary file 2 — jo4c00135_si_002.zip [file jo4c00135_si_002.zip › Fidy/15b/1/pdata/1/thumb.png]
